# A compact, wireless fluorimeter system for continuous, real-time monitoring of tissue perfusion via in vivo fluorescence signals

**DOI:** 10.1126/sciadv.aeg8187

**Published:** 2026-09-18

**Authors:** Tae Wan Park, Haohui Zhang, Chanho Park, Seth D. Goldstein, Lindsey Wade, Eric Rytkin, Alicia J. McLuckie, Yalcin Kulahci, Ramu Janarthanan, Tae Yeon Kim, Min-Seung Jo, Bedreddin Sazoglu, Zeynep Demir, Omer Dirican, Fatma Esra Demir, Woo-Youl Maeng, Seonggwang Yoo, Peter Saba, Kaiqing Zhang, Hak-Young Ahn, Jong Uk Kim, Jamin Lee, Anthony Banks, Seyong Oh, Kyeongha Kwon, Alan Nugent, Igor R. Efimov, Vijay S. Gorantla, Andrew Pelech, Yonggang Huang, John A. Rogers

**Affiliations:** ^1^ Querrey Simpson Institute for Bioelectronics, Northwestern University, Evanston, IL 60208, USA.; ^2^ Department of Civil and Environmental Engineering, Northwestern University, Evanston, IL 60208, USA.; ^3^ Department of Chemistry, Sejong University, 209 Neungdong-ro, Gwangjin-gu, Seoul 05006, Republic of Korea.; ^4^ Department of Surgery, Northwestern University Feinberg School of Medicine, Chicago, IL 60611, USA.; ^5^ Division of Pediatric Surgery, Ann & Robert H. Lurie Children’s Hospital, Chicago, IL 60611, USA.; ^6^ Department of Biomedical Engineering, Northwestern University, Evanston, IL 60208, USA.; ^7^ Center for Comparative Medicine, Northwestern University, Chicago, IL 60611, USA.; ^8^ Department of Surgery, Advocate Health, Wake Forest University School of Medicine, Winston-Salem, NC 27101, USA.; ^9^ Wake Forest Institute for Regenerative Medicine, Wake Forest School of Medicine, Winston-Salem, NC 27101, USA.; ^10^ School of Glocal Leaders, Glocal Honors College, Inje University, Gimhae 50834, Republic of Korea.; ^11^ Department of Digital Anti-aging Healthcare, Graduate School of Inje University, Gimhae 50834, Republic of Korea.; ^12^ School of Chemical Engineering, Sungkyunkwan University, Suwon 16419, Republic of Korea.; ^13^ Regenerative Neurorehabilitation Laboratory, Shirley Ryan Ability Lab, Chicago, IL 60611, USA.; ^14^ Division of Electrical Engineering, Hanyang University ERICA, Ansan 15588, Republic of Korea.; ^15^ School of Electrical Engineering, Korea Advanced Institute of Science and Technology, Daejeon, Republic of Korea.; ^16^ Graduate School of Artificial Intelligence Semiconductor, Korea Advanced Institute of Science and Technology, Daejeon, Republic of Korea.; ^17^ Department of Pediatrics, Northwestern University Feinberg School of Medicine, Chicago, IL 60611, USA.; ^18^ Pediatric Cardiology, Ann & Robert Lurie Children’s Hospital, Chicago, IL 60611, USA.; ^19^ Department of Materials Science and Engineering, Northwestern University, Evanston, IL 60208, USA.; ^20^ Department of Mechanical Engineering, Northwestern University, Evanston, IL 60208, USA.; ^21^ Department of Neurological Surgery, Northwestern University Feinberg School of Medicine, Chicago, IL 60611, USA.

## Abstract

In vivo fluorescence signals provide physiological and pathological insights for the interpretation of biological states associated with anatomical abnormalities or disease. Conventional methods for measuring fluorescence signals rely on bulky, costly instrumentation for imaging or for localized sensing. Here, we report a compact, wireless fluorimeter system for continuous, real-time measurements of the dynamics of fluorescence signals at locations of interest across the body. The approach represents a type of wearable that enables quantitative, interpretable fluorescence readouts and automatic data streaming to standard smart devices. The platform is compatible with injectable fluorescent species of medical relevance, such as indocyanine green, fluorescein, and methylene blue. Measurements from ischemic rodent and healthy porcine models, as well as those associated with limb amputation/replantation studies in rodents, demonstrate physiologically meaningful fluorescence profiles, with validation against ex situ blood analysis. These conceptual and technical advances offer the potential for convenient, continuous monitoring of fluorescence signals to support disease management and long-term patient care.

## INTRODUCTION

In vivo fluorescence sensing is a core bioanalytical technology that enables functional interrogation with physiological and pathological relevance (*1*, *2*). Fluorescent agents generate optical signals through interaction with the biological environment, enabling their use as optical biomarkers to capture spatial and temporal biological changes (*3*, *4*). Fluorescence signals can directly reflect dynamic biological processes, such as blood flow, metabolic activity, molecular clearance, or tissue permeability (*5*, *6*). Such information can provide critical insights into the assessment and interpretation of biological states before overt anatomical abnormalities or other clinically detectable disease-related signals become evident (*7*–*9*).

In vivo fluorescence sensing can be broadly classified into endogenous and exogenous approaches on the basis of the origin of the signal. Endogenous fluorescence originates from intrinsic metabolic cofactors and structural biomolecules within the body, such as reduced nicotinamide adenine dinucleotide, oxidized flavins, collagen, and lipofuscin (*10*–*12*). These responses are widely used in the context of human health to explore native physiological states, including tissue metabolism and structural composition (*13*, *14*). In contrast, exogenous fluorescence sensing relies on externally administered agents to access complementary biological information from target functions that do not exhibit intrinsic fluorescence (*15*, *16*). As examples, indocyanine green (ICG) and methylene blue (MB) are US Food and Drug Administration–approved fluorophores routinely used in clinical and biomedical applications to visualize anatomical features, such as blood and lymphatic vessels, the gastrointestinal tract, bile duct, and ureters (*3*, *16*–*19*).

A variety of strategies exist for detecting fluorescence in these cases, including imaging-based platforms and localized sensors. The former combine excitation and emission filtering with charge-coupled devices or complementary metal-oxide semiconductor cameras to visualize the spatial distribution of fluorescent agents across targeted tissues or whole organisms (*20*, *21*). These systems can be used in preclinical and intraoperative settings for visualizing vascular networks, lymphatic drainage, or tumor margins (*22*–*24*). Imaging can also be applied to assess tissue perfusion and vascular integrity during and after organ transplantation, confirming qualitative indicators of surgical outcomes (*25*–*27*). Despite their widespread use in clinical situations, imaging techniques remain constrained by practical and technical considerations, including the requirement for bulky and costly instrumentation and a limited capability for quantitative assessment of fluorescence intensity.

Nonimaging optical sensing (OS) approaches based on localized photodetectors (PDs), as in isolation or distributed arrays, can address these challenges. Such systems can perform in vivo sensing of neural activity in the deep brain (*28*–*30*), monitoring of doxorubicin pharmacokinetics in subcutaneous tissue (*31*), and tracking of pancreatic cancer cells (*32*). Multimodal optoelectronic arrays that integrate OS modules for fluorescent reporters with transparent electrodes can simultaneously record spatiotemporal electrical and optical parameters relevant to cardiac activation in vivo (*33*). Such methods are, however, inherently invasive or apply only to localized sensing within specific anatomical sites.

Here, we report a compact, wireless, skin-mounted fluorimeter system that supports continuous, real-time monitoring of in vivo fluorescence signals from the surface of the skin at locations of interest across the body. The system, as a type of wearable, captures fluorescence signal dynamics in underlying tissues and vessels via transcutaneous optical excitation and detection at tissue-penetrating wavelengths enabled by light-emitting diodes (LEDs), PDs, and optical filters tailored to specific fluorophores. A simple, clip-type form factor facilitates stable mounting onto multiple anatomical sites, enabling noninvasive, multisite monitoring of spatiotemporal variations in in vivo fluorescence signals. Wireless streaming of data follows from the use of a Bluetooth Low Energy (BLE) system-on-a-chip (SoC) platform that combines a central processing unit (CPU), an analog-to-digital converter (ADC), and a transimpedance amplifier (TIA). The platform is compatible with diverse fluorescent species of medical relevance, such as ICG, fluorescein, and MB, with simple modifications of the optical components. Measurements from ischemic rodent models demonstrate reliable sensing under simulated conditions with and without a fluorophore. Additional studies in this model focus on effects during limb amputation/replantation. Results from porcine models highlight the dynamic behavior of fluorescence signals following ICG administration, validating physiologically meaningful profiles in a large animal model that captures the complexity of the human circulatory system. This technology has the potential to improve patient care and quality of life by providing objective, accurate, and continuous real-time monitoring in various clinical settings.

## RESULTS

### Compact, wireless system for continuous fluorescence monitoring

**Fig. 1.**
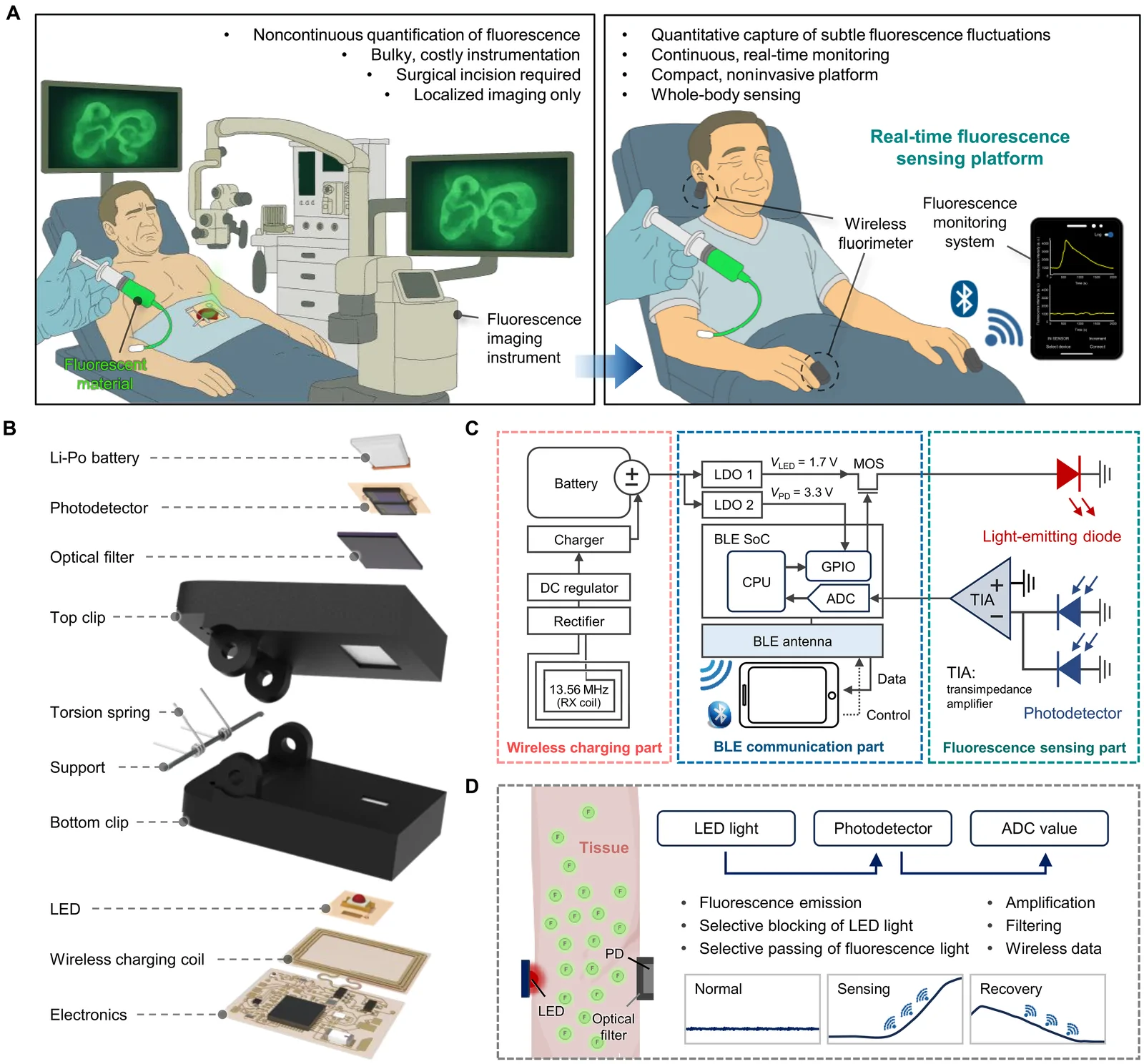
Compact wireless system for real-time fluorescence monitoring. (**A**) Schematic illustration of the clinical use case, showing wireless sensing of fluorescence signals emitted from a fluorescent material injected into the body. The compact system enables accurate, continuous, and noninvasive monitoring of in vivo fluorescence signals across multiple anatomical sites without bulky and costly instrumentation. The device wirelessly collects the signals, converts them into quantitative readouts, and automatically streams and stores the data on a user-friendly smart device in real time, thereby enabling long-term monitoring of fluorescence dynamics. (**B**) Exploded-view illustration of the wireless fluorimeter. (**C**) Block diagram of the electronics design, consisting of wireless charging, BLE communication, and OS modules. (**D**) Flow diagram for the strategy to detect fluorescence signals in tissue as electrical values. The fluorescence monitoring system enables the detection of subtle fluctuations in fluorescence intensity and wirelessly transmits the data to a smart device for real-time visualization.

The wireless, skin-mounted system introduced here detects subtle fluctuations in fluorescence intensity arising because of emission from underlying tissues and vessels via transcutaneous optical excitation and detection at tissue-penetrating optical wavelengths. Data transfer to a smart device for storage and calibrated processing allows for continuous, real-time display of time-dependent changes in fluorescence. Figure 1A portrays an envisioned use case for monitoring of fluorescence generated by a clinically approved chemical species introduced into the bloodstream using standard methods. The compact, wearable nature of this technology overcomes key disadvantages of conventional, imaging-based medical systems, which include high costs, large dimensions, and limited measurement times (table S1). An exploded-view schematic illustration in Fig. 1B highlights the device architecture and key components that include an LED, an optical filter, a PD, and wireless electronics. Top and bottom clips, along with support and torsion springs, provide for a simple mechanical means to interface with suitable anatomical sites, such as the earlobes or fingers, without the need for adhesives or additional mechanical fixtures (see Materials and Methods and fig. S1 for details). Openings in these clips define windows with dimensions that match the sizes of the LED (1.6 mm by 3.2 mm) and PD (4 mm by 5.09 mm) components.

The engineering approach incorporates three key parts: (i) an OS module to excite and detect fluorescence; (ii) a BLE SoC module for system control, wireless operation and data transfer; and (iii) a rechargeable battery with wireless charging circuitry (Fig. 1C, fig. S2, and note S1). The OS module consists of an LED and a PD oriented toward each other. A thin film–type optical filter that covers the PD prevents transmission across a band of wavelengths centered around the excitation wavelength (i.e., the wavelength of emission from the LED).

The BLE SoC provides a 3.0-V gate signal through a general-purpose input/output (GPIO) port to an N-channel metal oxide semiconductor field-effect transistor (MOSFET). In this configuration, the source of the MOSFET connects to the LED, and the drain connects to a low-dropout (LDO) regulator that regulates the battery voltage to 1.7 V. The result is a regulated bias that can be switched on or off depending on the general-purpose input/output powers the LED. The optical characteristics of the LED appear in figs. S3 and S4, selected for operation in the near-infrared (NIR) range to ensure deep tissue penetration. The optical power increases stepwise as the input power varies from 0 to 0.06 W (fig. S3). Furthermore, increasing the resistance of a resistor connected to the LED from 0 to 100 kΩ leads to a corresponding decrease in the optical emission power as an additional electrical parameter for tunable optical control (fig. S4).

Fluorescence generated by illumination of the targeted chemical species by the output of the LED generates a response in the PD. A TIA, consisting of an operational amplifier and a feedback loop (1.4-MΩ resistor and 4.7-nF capacitor), converts the PD output current into a voltage (*V*_PD_). The ADC within the BLE SoC captures the *V*_PD_ at a sampling rate of 100 Hz. Data communication between the BLE SoC and a smart device follows standard protocols for graphical display. The system operates at a low power consumption, drawing only 0.14 mA in standby mode and 4.58 mA in active mode at 3 V with a data rate of 400 bytes/s. Figure S5 presents detailed results during operation in standby and active modes. Figure 1D illustrates a flow diagram for detecting fluorescence with a user interface that controls the LED operation and performs data logging. The system wirelessly collects, continuously displays, and automatically stores the fluorescence in real time (fig. S6).

### Engineering designs and calibration procedures

**Fig. 2.**
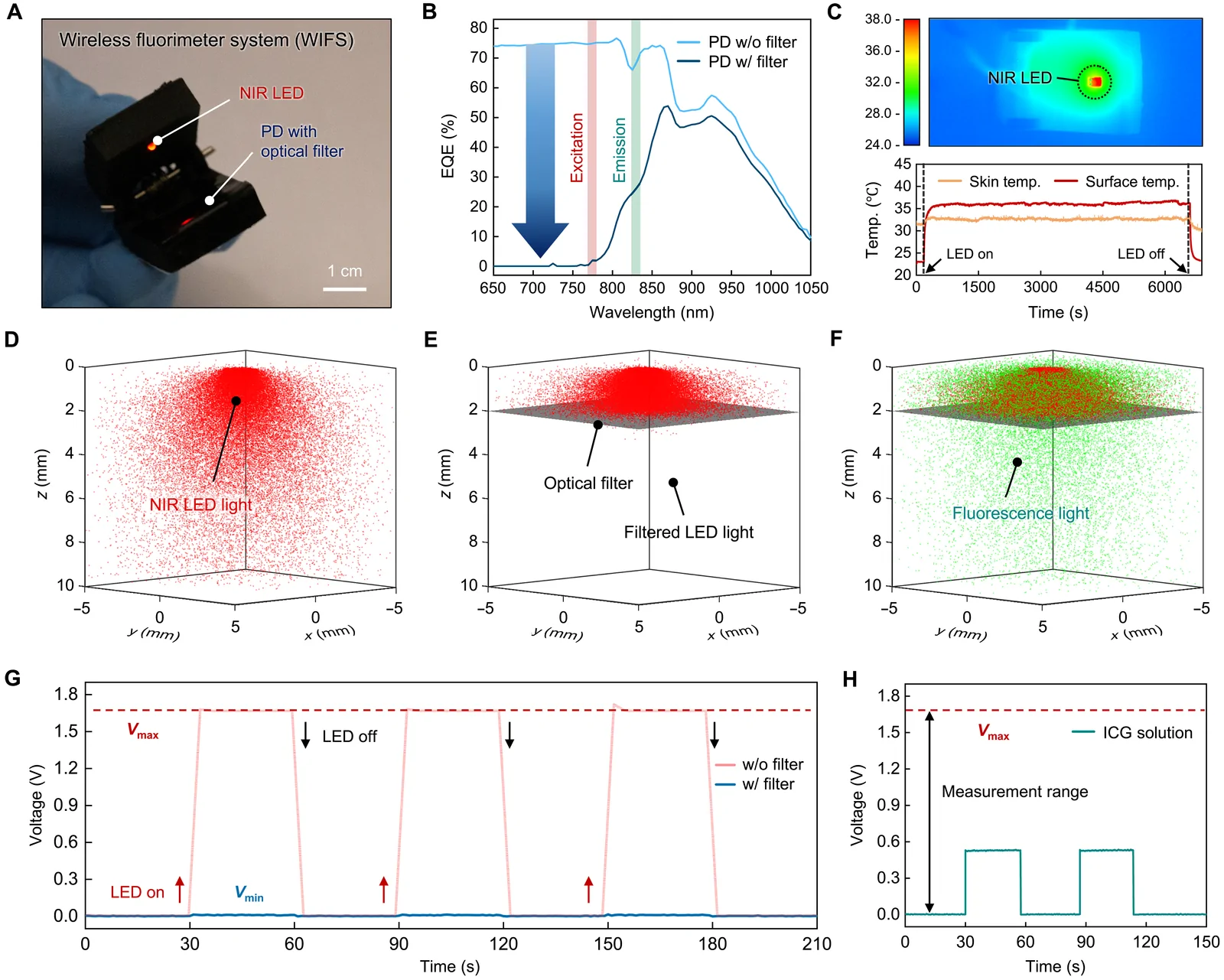
Engineering aspects for dynamic sensing of a fluorophore. (**A**) Image of the clip-type wireless fluorimeter while the NIR LED is active. (**B**) EQE spectra of a PD with (dark blue) and without (light blue) an optical filter. The red and green areas indicate the peak wavelength of the LED and emission wavelength of ICG, respectively. (**C**) Thermal images and time series temperature data measured across the device, at the location of the NIR LED, and at the interface between the device and skin during normal operation (107 min and 1.78 hours). Monte Carlo simulations for the cases of (**D)** LED-PD, (**E**) LED-optical filter-PD, and (**F**) LED-fluorescent material-optical filter-PD. The optical filter blocks LED emission with an efficiency of 99.48%. (**G**) Signal calibration and system validation using an optical filter without fluorescent material. (**H**) Fluorescence signal displayed between *V*_0_ and *V*_max_.

Figure 2 describes methods for dynamic sensing using a wireless fluorimeter system. The compact size (20 mm by 32.5 mm by 23 mm) and a lightweight construction (9.7 g) enable practical use across a wide range of anatomical sites with varying tissue thicknesses in ways that are difficult or impossible to replicate with conventional medical systems (fig. S7 and table S1). Initial validation studies use ICG as the fluorophore, a clinically approved green dye widely used in diverse surgical and clinical applications for more than half a century, including tumor lymphangiography, ophthalmic angiography, hepatic function assessment in transplant, cirrhosis, and septic shock (*3*, *19*, *25*, *27*). Therefore, validating the wireless system described here with ICG captures its potential applicability across a broad range of fluorescence-guided applications. Figure S8 (A and B) shows the chemical structure and the absorbance (peak at 779 nm) and emission (peak at 823 nm) profiles of ICG. An NIR LED with a peak emission wavelength of 770 nm and an IR PD with a maximum sensitivity at 830 nm represent good choices for this application. The optical filter efficiently blocks the LED light and selectively passes the fluorescence. Figure S8C illustrates the experimental strategy. Figure 2B shows the external quantum efficiency (EQE) spectra of the PD with (dark blue) and without (light blue) the filter. The red and green areas indicate wavelength ranges of emission from the NIR LED and fluorescence from ICG, respectively. The optical filter selectively passes the fluorescent light and rejects the LED light, with less than 0.6% transmittance for light below a wavelength of 800 nm and greater than 38% transmittance for light above a wavelength of 830 nm.

The thermal load imposed on the skin is an important consideration for safe operation. At a constant input current of 30 mA, a typical value for operation, the surface temperature of the NIR LED increases to a constant value of 37.1°C after 300 s (Fig. 2C and fig. S9). In addition, the temperature at the interface between the skin and the LED remains stable below 34°C after device mounting (Fig. 2C). This result meets the medical safety standards for skin contact in both animals and humans (*34*, *35*). Previous reports indicate that NIR illumination with wavelengths and power levels comparable to those used in this study does not induce applicable adverse effects, such as photothermal injury or internal tissue damage, even under repeated or prolonged exposure (*36*–*39*). Blood perfusion facilitates the replenishment of injected fluorophores. This process, together with the relatively low-intensity and broad spectral emission of LED light used here, avoids photobleaching effects that can be observed with laser-based excitation (*40*).

Optical simulations based on Monte Carlo methods reveal distributions of intensity for light from the LED and from fluorescence with and without the optical filter. Figure 2 (D to F) presents results that compare measurements using the following configurations: LED-PD, LED-optical filter-PD, and LED-ICG-optical filter-PD. The simulation uses the wavelength-dependent molar absorption coefficient of ICG, $\varepsilon_{a,\text{ICG}}\left(\lambda \right)$, as experimentally obtained from the absorption profile in fig. S8B, with the assumption that the optical absorption in the solution arises solely from ICG, given that water absorption is negligible compared to ICG over the wavelength range of 750 to 860 nm (*41*). The ICG concentration is 5 μg/ml, and the reduced scattering coefficient of the solution is zero. The modeling domain consists of 200 by 200 by 100 voxels, each with a size of 0.1 mm by 0.1 mm by 0.1 mm, corresponding to a total volume of 20 mm by 20 mm by 10 mm. A Lambertian source with a circular emission area of 1.5 mm in diameter, located at the center of the top surface of the solution and oriented downward, represents the LED with an emission spectrum in fig. S8C. To account for wavelength-dependent absorption, the model performs independent simulations at wavelengths from 750 to 820 nm in 5-nm increments. The total absorbed power in each voxel corresponds to the sum of the contributions from all wavelengths, weighted by the LED power at each wavelength. The total LED power remains constant throughout the simulation. For the fluorescence simulation, each voxel is an isotropic emitter. The quantum yield of ICG is 0.13 (*42*); accordingly, the total fluorescence power emitted by each voxel is 13% of the absorbed power in that voxel, with the spectral distribution determined by the intrinsic ICG emission spectrum in fig. S8C. This modeling also simulates fluorescence propagation independently at wavelengths from 780 to 860 nm in 5-nm intervals. At the bottom surface, photons within the sensor area (5.7 mm by 2.85 mm) contribute to the received power at each wavelength.

The total received LED and fluorescence power without the optical filter corresponds to the sum of the power over all wavelengths. With the filter applied, the power at each wavelength is first multiplied by the corresponding filter transmission efficiency and then summed to yield the total filtered LED and fluorescence power. Although the received power from the fluorescence is only 8.4% of that from the LED, the latter has a wavelength mostly below 800 nm. The optical filter largely eliminates this contribution with an efficiency of 99.48%, thereby allowing the fluorescence to dominate the detected signal. In particular, the fluorescence power received at the PD is nearly four times larger than the highly attenuated LED power transmitted through the optical filter, ensuring successful fluorescence sensing. The simulated intensity distributions at the bottom surface (fig. S10A), in a cross-sectional plane (fig. S10B), and at the bottom surface along X=0 (fig. S10C), provide an intuitive visualization of the power values received at the PD. Additional discussions address how photophysical factors affect the quantitative interpretation of the in vivo fluorescence signal (fig. S11 and note S2).

Figure 2G presents the process of signal calibration and validation by comparison of these simulation results to experimental measurements obtained using a wireless fluorimeter and an optical filter without the fluorophore. The PD yields maximum and minimum voltages (*V*_max_ and *V*_min_) of 1.67 and 0.01 V when operated without and with an optical filter, respectively. The LED light rejection efficiency calculated from experiments is 99.38%, in excellent agreement with the simulation result. Measurements on an ICG solution dissolved in deionized (DI) water yield distinguishable fluorescence across the full range from 0 to *V*_max_ (Fig. 2H). The measurements require precise optical alignment between the excitation source and the PD, emphasizing the importance of a device structure that maintains stable alignment (fig. S12). The sensitivity and response speed of the electronics can be adjusted through selection of resistor and capacitor values in the TIA (fig. S13); for experiments reported here, these values are 1.4 MΩ and 4.7 nF, respectively (*43*). This sensor detects ICG concentrations down to 30 ng/ml, defined as the limit of detection, determined using a 60-s integration time and calculated as the means and three standard deviations (3σ) of the blank (fig. S14).

### Sensing characteristics

**Fig. 3.**
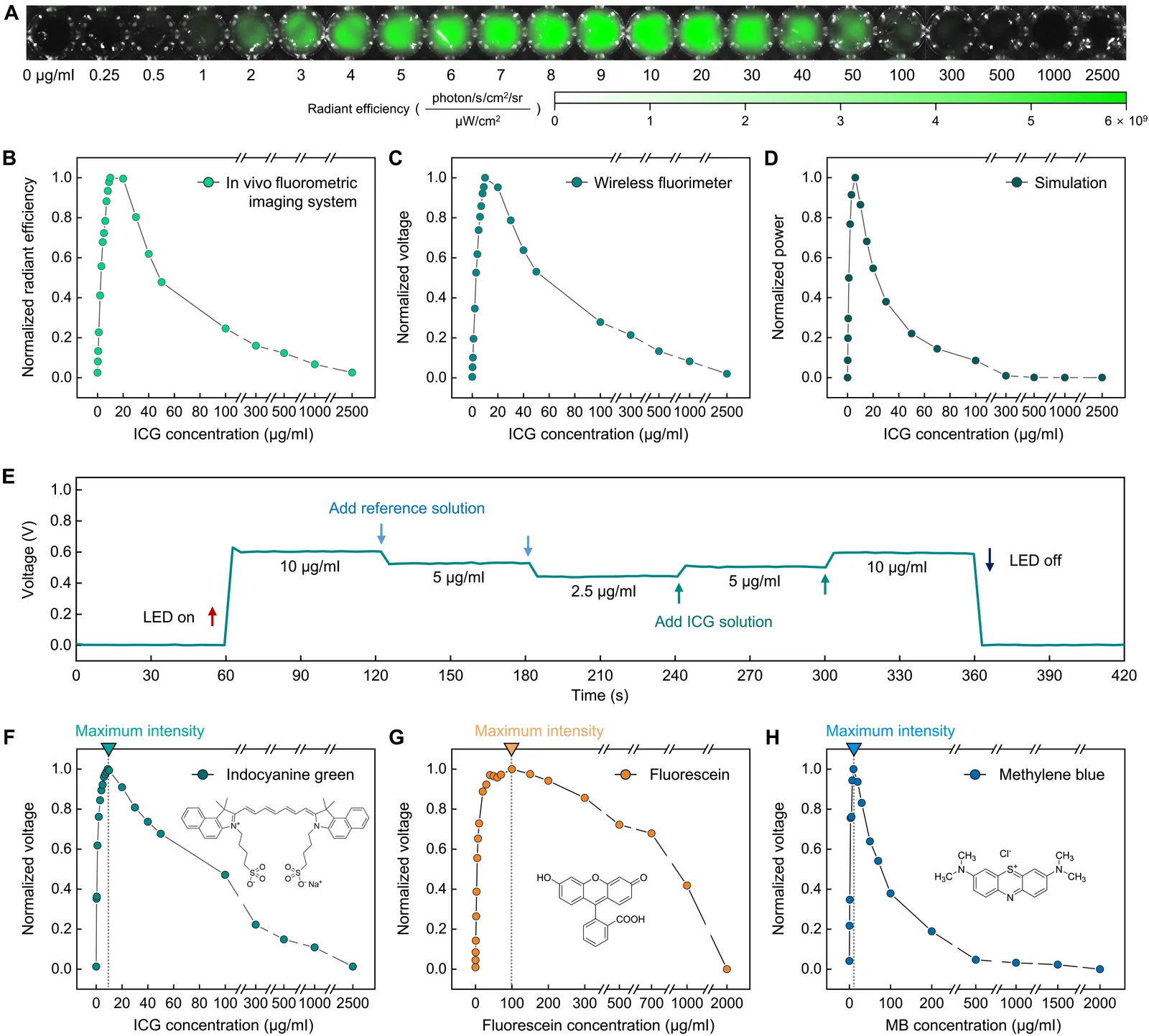
Fluorescence sensing characteristics. (**A**) Fluorescence signal as a function of concentration of ICG dissolved in porcine blood captured using an in vivo fluorometric imaging system. Experimental measurements derived from (**B**) fluorescence images and (**C**) wireless system with concentrations from 0 to 2500 μg/ml. (**D**) Simulation results for these experiments. (**E**) Sensing performance for subtle variations in ICG concentration. Results demonstrating applicability to various fluorophores, including (**F**) ICG, (**G**) fluorescein, and (**H**) methylene blue. Detection of diverse injectable fluorophores is possible with simple modifications of the OS module.

Experiments with different fluorescent species illustrate the performance of the device and highlight its versatility, as summarized in Fig. 3. The first results feature the response of the system to ICG dissolved in DI water at concentrations from 0 to 2500 μg/ml, as in fig. S15. Figure S15A presents a photograph of the ICG solutions, showing different colors depending on the concentration. The concentration dependence of ICG fluorescence, determined using an in vivo fluorometric imaging system (fig. S15, B and C), the wireless fluorimeter (fig. S15, D and E), an NIR biomolecular imager (fig. S15, F and G), and a microplate fluorescence reader (fig. S15, H and I), exhibits a nonmonotonic behavior resulting from reabsorption at high concentrations, reaching a maximum intensity at 10 μg/ml, in agreement with theoretical analysis (fig. S15J).

Figure 3A displays fluorescence images of ICG material dissolved in porcine blood captured by the imaging system, consistent with measurements with the wireless fluorimeter and with simulations (Fig. 3, B to D). In Fig. 3D, the simulation indicates that the received intensity increases with concentration because of enhanced fluorescence emission at low concentrations (≤10 μg/ml). At higher concentrations (>20 μg/ml), reabsorption of the emitted fluorescence by the ICG results in a decrease (*44*). The device can accurately read subtle changes in fluorescence associated with small variations in ICG concentration (Fig. 3E). The fluorescence at a given concentration remains stable over 12 hours (fig. S16).

The system is compatible with other fluorescent species of medical relevance, such as fluorescein and MB (Fig. 3, F to H). The former is widely used in ophthalmology and clinical angiography to monitor dynamic changes in microvascular perfusion and vascular permeability (*45*). An LED with a peak emission at 480 nm, an optical filter blocking wavelengths below 510 nm, and a PD with maximum sensitivity at 533 nm align with the absorption and emission properties of fluorescein (fig. S17). Systematic benchtop studies confirm that a device configured in this manner yields results that agree with those from a standard imaging system (fig. S18). Figure S19 presents the fluorescence sensing strategy for MB, a US Food and Drug Administration–approved fluorescence dye widely used in clinical diagnostics and surgery to visualize lymphatic drainage and sentinel lymph nodes (*3*, *46*). Here, a device with an LED with a peak emission at 675 nm, an optical filter blocking wavelengths below 679 nm, and a PD with maximum sensitivity at 705 nm allows for accurate measurements with MB sensing (fig. S20), in agreement with standard imaging. In vivo measurements of these fluorophores reinforce the versatility of the fluorescence sensing platform reported here (fig. S21 and note S3).

### In vivo demonstrations in rodent ischemia-reperfusion models

**Fig. 4.**
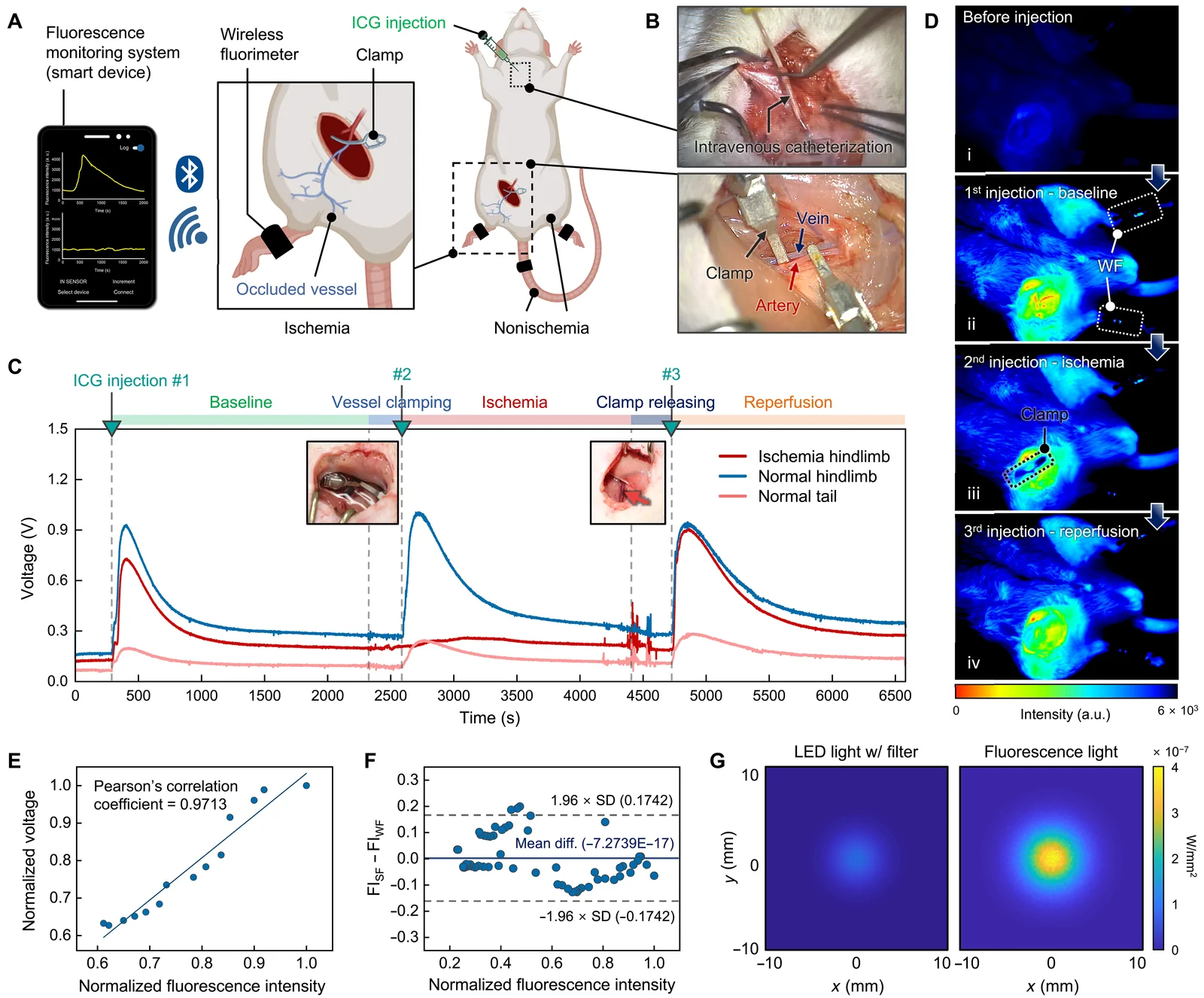
In vivo demonstration of wireless fluorimetry in rodent ischemia models. (**A**) Schematic illustration of the small animal ischemia model. Created in BioRender. Rogers, J. (2026), https://biorender.com/vk3u5ep. (**B**) Optical microscope images of catheter insertion for material injection and vessel clamping to induce ischemia. (**C**) Long-term monitoring of fluorescence response dynamics during three phases of baseline, ischemia, and reperfusion. (**D**) Sequential fluorescence images acquired before and after injections. a.u., arbitrary units. (**E**) Correlation between measurements using a standard fluorescence imager and the wireless fluorimeter, with a Pearson’s correlation coefficient of 0.97. (**F**) Bland-Altman plot comparing fluorescence trends determined using the wireless fluorimeter and the standard fluorescence imager. (**G**) Monte Carlo simulation results for fluorescence light efficiency: (left) filter-blocked LED light and (right) filter-transmitted fluorescence light on the rat paw.

In vivo studies on rodent models involve mounting the devices on the hindlimbs and tail, preparing one hindlimb as an ischemia model, delivering ICG, and performing wireless measurements of fluorescence (Fig. 4). The device architectures for placement on the paw (fig. S22A) and tail (fig. S22B) slightly differ owing to their respective target thicknesses of 4.7 and 7 mm (figs. S23 and S24). Ex vivo studies performed in the absence of the fluorophore confirm reference values on the basis of thickness-dependent signal measurements (fig. S24A). The surgical procedures and experimental protocols for the ischemia model appear in Fig. 4B and figs. S25 and S26, with details in Materials and Methods. Administration of ICG occurs three times for each of the baseline, ischemia, and reperfusion phases. Monitoring during each phase extends for 30 min to capture both the increase in fluorescence induced by ICG administration and the subsequent decrease driven by blood perfusion. Vascular clamps temporarily occlude the dissected artery and vein before the ischemia step before subsequent release during the reperfusion step to restore blood flow.

Figure 4C presents measurements from the ischemic hindlimb, normal hindlimb, and normal tail, demonstrating distinct signal dynamics associated with each physiological condition. In the first baseline step, the fluorescence of both hindlimbs exhibits a similar trend. The signal rapidly increases immediately after ICG injection with a dose of 1.5 ml/kg and progressively decreases over time, indicating a natural physiological response in the healthy model (note S4). The signal on the tail exhibits lower fluorescence than that observed on the hindlimbs, primarily due to differences in target tissue thicknesses. Surgical clamps that occlude the dissected artery and vein at the incised site of the right hindlimb establish the ischemia model.

The second ischemia step involves a second ICG administration. In the normal left hindlimb (blue line), the fluorescence increases rapidly immediately after ICG administration, followed by a gradual decrease over time, similar to the baseline step. In contrast, the right hindlimb with occluded blood flow shows no increase in fluorescence following ICG administration because perfusion does not occur in this hindlimb. This observation indicates that the injected ICG circulates well, demonstrating that the ischemia model is achieved as intended. Furthermore, the negligible increase in fluorescence supports the ability to detect the absence of fluorescence enhancement when ICG enters tissue without blood perfusion.

The third reperfusion step involves injecting the same dose of ICG again to evaluate recovery in the right hindlimb, which experienced ischemia. The increase in fluorescence after ICG injection matches that of the left normal hindlimb, showing complete recovery. In general, tissue damage or necrosis occurs after more than 3 hours of blood flow occlusion (*47*). The protocols here thus avoid damage. For comparison, the studies include an in vivo fluorometric imager that operates continuously during the entire protocol (fig. S27). Figure 4D presents images (i) before injection and (ii to iv) immediately after ICG administration in each of the three phases (fig. S28). The wireless measurements (fig. S29A) are in good agreement with these results (fig. S29B).

Figure 4E shows a correlation between fluorescence images and measurements using the wireless device on the ischemic right hindlimb during the ischemia phase, indicating >0.97 of Pearson’s correlation coefficient. All measurements in the ischemic right hindlimb, normal left hindlimb, and normal tail during three phases of baseline, ischemia, and reperfusion exhibit Pearson’s correlation coefficients exceeding 0.94 (fig. S30). The Bland-Altman plots in Fig. 4F and fig. S31 provide additional means for comparison. During the entire protocol, the NIR LED maintains stable operation with an optical power density of 1.55 mW/mm^2^ (fig. S32 and note S5). Collectively, these results verify capabilities for continuous, reliable real-time monitoring of fluorescence fluctuations with the device introduced here.

Monte Carlo simulation results in Fig. 4G and fig. S33A show the fluorescence efficiency in the rat paw. The fluorescence at the bottom surface is the strongest directly beneath the LED. Owing to the high efficiency of the optical filter, the fluorescence is substantially stronger than the residual LED illumination, ensuring reliable sensing. The ratios, defined by normalizing the peak fluorescence after ICG injection to the value before injection, are 5.52 from both measurements and simulation. Figure S33 (B to D) displays intensity distributions in a three-dimensional (3D) view, a cross-sectional plane, and the bottom surface along X=0. Figure S34 summarizes results in a cylindrical rat tail, confirming that the performance on flat anatomical surfaces is comparable to that on curved or cylindrical surfaces.

### Postoperative tissue perfusion and vascular integrity

**Fig. 5.**
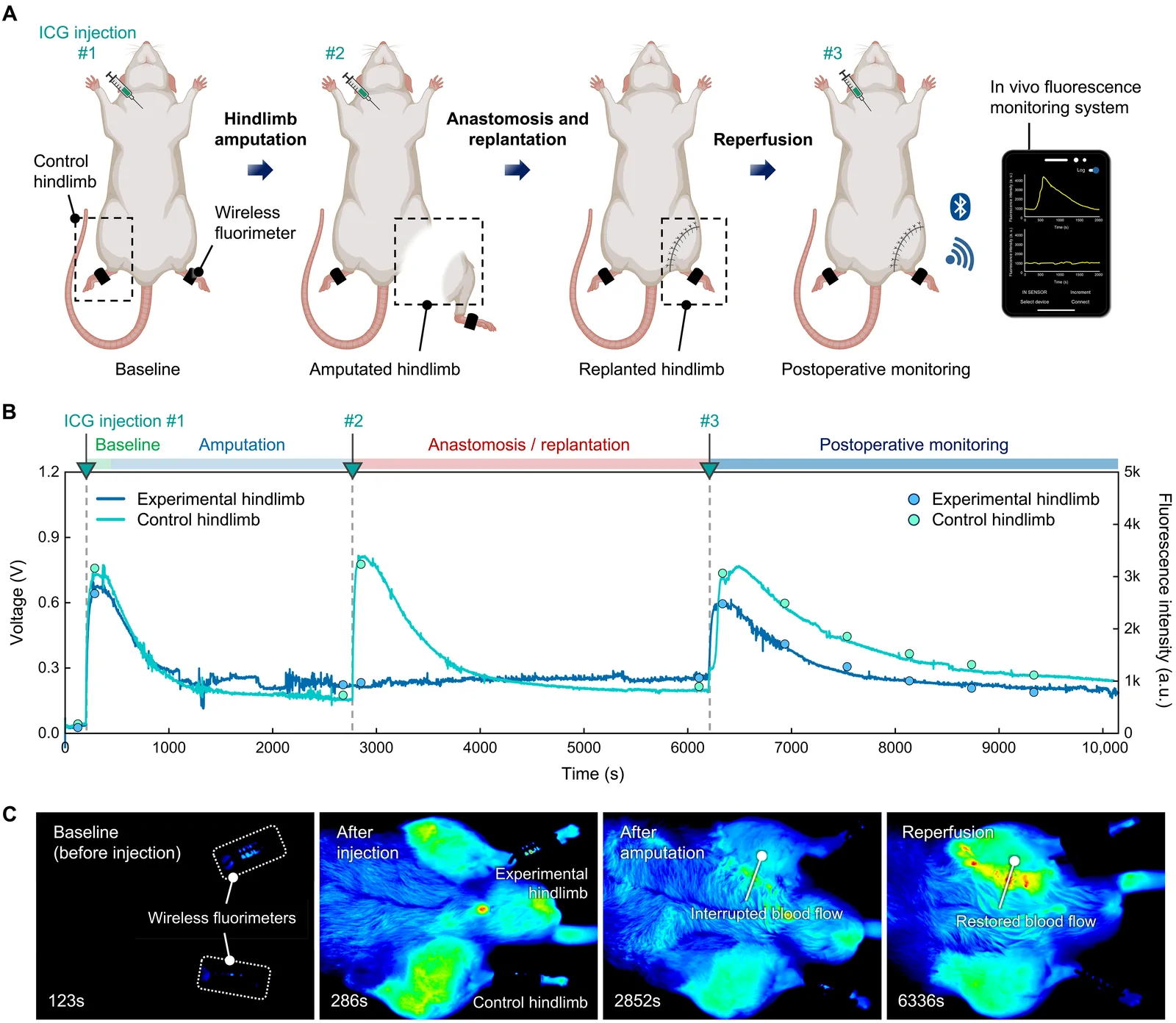
Postoperative tissue perfusion monitoring. (**A**) Schematic illustration of the hindlimb amputation and replantation model. The wireless fluorimeter enables accurate and continuous monitoring of in vivo fluorescence signals for the assessment of postoperative blood circulation following hindlimb replantation. Created in BioRender. Rogers, J. (2026), https://biorender.com/vk3u5ep. (**B**) Real-time monitoring of fluorescence dynamics during amputation, replantation, and postoperative recovery. Continuous profiles and discrete dots correspond to the voltage readouts obtained from the wireless fluorimeter and the fluorescence intensities captured by the fluorometric imager, respectively. (**C**) Sequential fluorescence images acquired at the baseline (before and after injection), after amputation, and following reperfusion.

A more clinically relevant demonstration in rodent models involves mounting the device on the hindlimb, performing hindlimb amputation and replantation, administrating ICG, and monitoring fluorescence signals before and after replantation (Fig. 5). Unlike the acute ischemic model, this study incorporates complete vascular interruption followed by microvascular reconstruction and reperfusion, providing a clinically relevant model for evaluating fluorescence-based monitoring of vascularized composite tissues. The surgical procedures for postoperative tissue perfusion and vascular integrity monitoring are shown in fig. S35, with the details in Materials and Methods. Amputation and replantation of the left hindlimb allow for an evaluation of postoperative blood circulation using the wireless fluorimeter system, where the right hindlimb serves as a control. The systems monitor fluorescence responses during the entire protocol in which ICG administration occurs three times at the baseline, before vascular anastomosis, and postoperative reperfusion following replantation. Figure 5B shows the associated real-time fluorescence dynamics. Continuous line profiles and discrete dots represent the voltage readouts from the systems and the fluorescence intensities captured by the fluorometric imager, respectively.

The control hindlimb exhibits consistent fluorescence kinetics in all steps, with pronounced peaks after ICG injection followed by a gradual decrease over time, reflecting a natural physiological circulation. In the replanted hindlimb, the fluorescence response is comparable to that of the control hindlimb before amputation, whereas the signal shows no increase after the second ICG injection because of the absence of blood perfusion. Following microvascular anastomoses and hindlimb replantation, restoration of characteristic fluorescence kinetics recovery in the replanted hindlimb indicates successful reperfusion and recovery of vascular patency, demonstrating the ability of the device to continuously monitor perfusion dynamics following complex microsurgical reconstruction. The irregular voltage variations observed in the profiles during the amputation stage arise from substantial muscle contractions induced by the bipolar electrocautery used for amputation. The fluorescence intensities quantified from the images show strong consistency with the trends in the profiles (Fig. 5C and fig. S36).

### In vivo validation in large animals

**Fig. 6.**
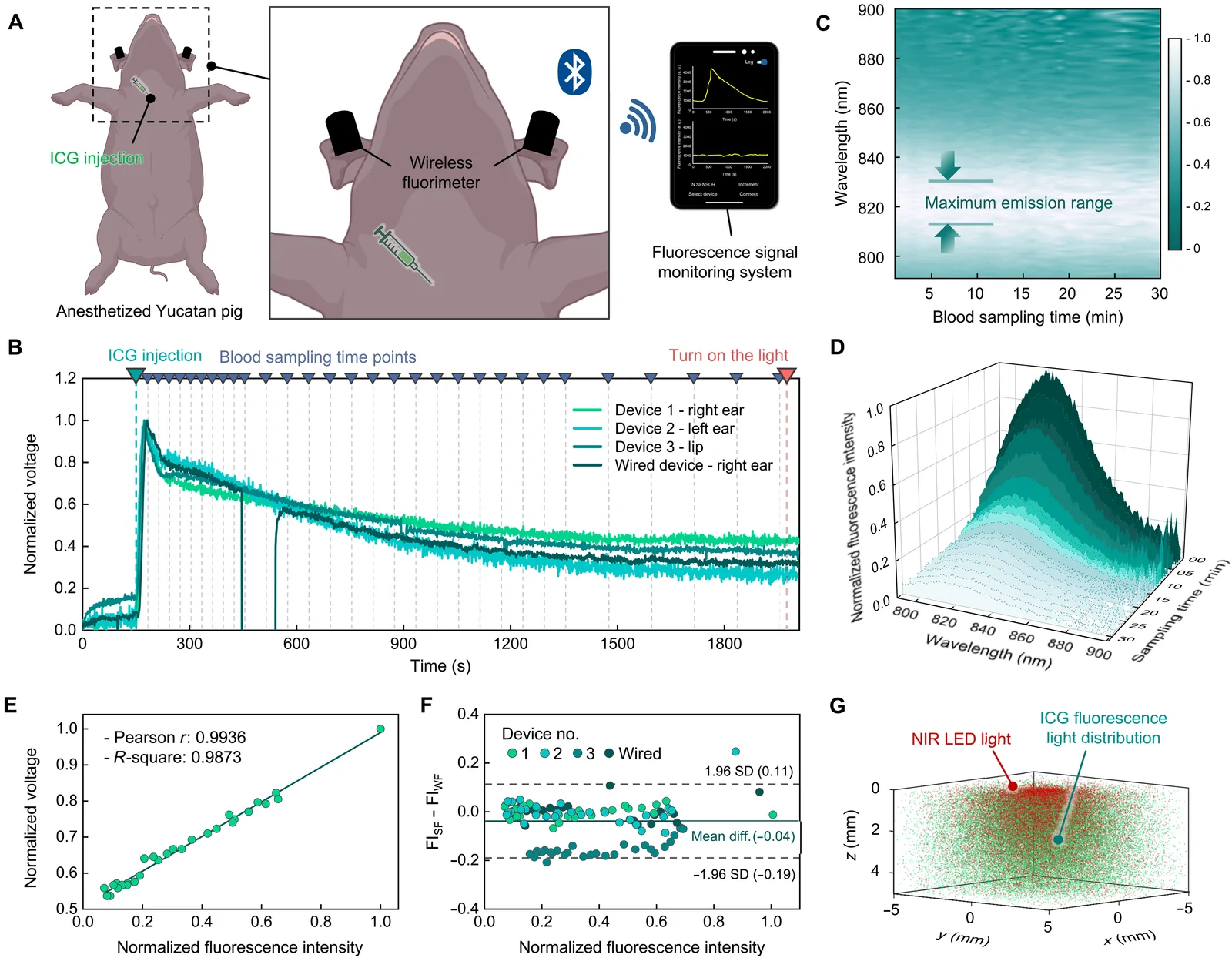
In vivo validation of wireless fluorimetry in porcine models. (**A**) Demonstration setup for the large animal study. Created in BioRender. Rogers, J. (2026), https://biorender.com/vk3u5ep. (**B**) Real-time fluorescence dynamics measured by wired and wireless fluorimeters. (**C**) Fluorescence intensity maps showing the peak emission wavelength of ICG from blood samples. (**D**) 3D emission spectra of changes in fluorescence intensity as a function of time after ICG injection. (**E**) Correlation between fluorescence intensity measured by a standard fluorometer and the wireless fluorimeter. (**F**) Corresponding Bland-Altman plot of fluorescence signal trends from four different devices. (**G**) 3D irradiance distributions of NIR LED light and ICG fluorescence signals in the pig ear model.

In vivo studies on porcine models involve mounting the wireless fluorimeter on the lip or ears, administering ICG material, and performing measurements of fluorescence using both the wireless device and a wired device connected to a commercial multichannel data acquisition system (PowerLab, 8/35, ADInstruments) (Fig. 6). Physiological systems in large animals are notably different than those of small animals because, as with humans, the response to the injectable fluorophore varies between individuals and the internal circulatory system exhibits high levels of complexity (*48*). Large animal studies with porcine models help to evaluate the translational scalability and clinical suitability for human applications.

The devices record fluorescence fluctuations and transmit the data wirelessly to a smart device in real time during the experimental protocol (Fig. 6A). The device dimensions for porcine studies illustrated in fig. S37 accommodate porcine ears and lips, which have thicknesses of 5 and 8 mm, respectively. Figure S38 shows the measurement setup with four devices [two wireless devices and one wired device (fig. S39) on the ears and one wireless device on the lip].

Figure 6B shows that the results align with physiologically meaningful profiles. The upstroke exhibits a rapid increase in fluorescence immediately after ICG injection and culminates at the peak, which corresponds to the point where ICG material distributes completely within the porcine body. Subsequently, a natural physiological decrease in fluorescence over time occurs because of blood perfusion and hepatic metabolic processes (*19*, *49*). Room lights do not affect the operation of the devices. Signal interruptions observed between 302 and 395 s after ICG injection in the wired device result from a temporary wire detachment, highlighting a disadvantage relative to a wireless mode of operation.

Additional comparisons rely on the analysis of blood samples drawn at 30-, 60-, and 120-s intervals after ICG injection until the end of the protocol, as indicated by the vertical blue dashed lines in Fig. 6B. The fluorescence from these blood samples serves as the ground truth for comparison to temporal profiles obtained from the devices. Benchtop-calibrated electrical readouts from blood samples with known concentrations enable quantitative estimation of in vivo ICG concentrations from the monitored voltage signals (fig. S40 and note S6). The fluorescence map and 1D emission spectra in Fig. 6C and fig. S41 show the expected peak emission wavelengths of ICG in the blood samples. The 3D emission spectra in Fig. 6D not only display the maximum fluorescence efficiency for each sample but also reveal the overall time dependence of the fluorescence in all samples.

Measurements from the devices and data from the blood samples (Fig. 6E and fig. S42) show high Pearson’s correlation values between voltage values and intensities. In particular, the values are 0.99, 0.97, 0.99, and 0.99 for devices on the right ear, left ear, and lip and for the wired device on the right ear, respectively. The mean difference and standard deviation in the corresponding Bland-Altman plot are −0.04 and 0.08, respectively (Fig. 6F). The simulation results in Fig. 6G and fig. S43 support capabilities for reliable fluorescence sensing in the porcine ear. Comparisons of simulated and experimentally measured ratios of received fluorescence to LED power in the pig ear experiment suggest that the ICG concentration is 2.17 μg/ml. The simulated maximum effective sensing depth of fluorescence signals after filtering is 11 mm (fig. S44). Additional discussion of the measurement accuracy and reproducibility explains the role of the absorption coefficients, tissue reduced scattering coefficients, and tissue thicknesses used in the simulations (fig. S45 and note S7).

Figure S46 shows that measurement results in additional porcine models (*n* = 3) are consistent with those described above. Figure S47 presents a device designed for mounting on a human finger. This device targets a finger thickness of 13 mm. Operation is also possible on the index, middle, and ring fingers, although the thumb and little finger fall outside the targeted range (fig. S48, tables S3 and S4, and note S8). The device also supports continuous monitoring for up to 33.5 hours without charging, and a fully depleted battery can be recharged wirelessly within only 6 hours at 13.56 MHz, a resonance frequency considered safe for human exposure (figs. S49 and S50). The ability to maintain a stable baseline signal under dynamic conditions further supports the robustness and reliability of the data acquisition using this device (fig. S51 and note S8). Models that consider the effects of skin, fat, muscle, and bone suggest feasibility for use in this manner (figs. S52 and S53), thus motivating translational efforts as next steps. Ex vivo demonstrations of fluorescence sensing in a whole human heart highlight the advantages of wireless and continuous monitoring with the system reported here (figs. S54 and S55 and note S9).

## DISCUSSION

The compact, wireless system reported here enables accurate, continuous, and noninvasive monitoring of in vivo fluorescence signals in real time. The system incorporates tailored combinations of LEDs, PDs, and optical filters to align with targeted fluorescent species. The electronics that interface to these optical components include a BLE SoC with a CPU, ADC, and TIA for quantitative readout of fluorescence intensity, wirelessly collected, continuously displayed, and automatically stored on a standard smart device. A clip-type form factor facilitates mounting on various anatomical sites, with capabilities in simultaneous multisite monitoring.

Conventional fluorescence measurement systems used in clinical settings predominantly rely on imaging-based techniques that require bulky and costly instrumentation or wired probes that demand surgical implantation. Such approaches hinder continuous quantitative data collection, typically require surgical incisions, and confine measurements to localized regions. The technology introduced here serves as a complementary method with potential for translation to clinical practice. Results from ischemic rodent models and postoperative tissue perfusion monitoring demonstrate quantitative and accurate assessments of spatiotemporal variations in fluorescence signals. Studies with porcine models, which exhibit interindividual variability in response to the injected fluorophore and have a substantially complex circulatory system similar to humans, validate physiologically meaningful fluorescence signal profiles.

This platform provides a comfortable user experience owing to its wireless and noninvasive operation. As a specific application, the technology may offer insights for monitoring of liver function in patients with liver diseases and hepatic lesions associated with clearance of ICG through hepatic metabolism. In surgical contexts, continuous intraoperative and postoperative perfusion monitoring in complex reconstructive or transplant surgery may enable objective assessments of graft viability and early detection of vascular compromise. Furthermore, continuous quantification of site-specific fluorescence kinetics with a wireless optical platform may provide a foundation for digital biomarkers that inform prognostic modeling and personalized therapeutic guidance. The simplicity and ease of use of the technology, together with utility in these and other clinical applications, underscore a strong translational potential.

## MATERIALS AND METHODS

### Fabrication of wireless electronics

Flexible printed circuit boards (fPCBs; PCBway) based on polyimide films with thicknesses of 0.15 mm served as substrates for the electronics. The layout for the fPCB was created by Autodesk Fusion (Autodesk). Mounting of the electrical components relied on manual positioning with solder paste (Sn42/Bi57/Ag1, NC191LTA15T5, Chip Quik) dispensed onto the Au pads of the fPCB and melted at 170°C for 10 s using a hot air gun. These components included a BLE SoC (nRF52832, Nordic Semiconductor), a BLE antenna (2450AT18A100, Johanson Technology Inc.), an N-MOSFET (CSD17381F4, Texas Instruments), a 3.0-V LDO regulator (TCR3UM33A, Toshiba), and a 1.7-V LDO regulator (TCR5RG17A, Toshiba). The OS module incorporated an NIR LED (peak emission wavelength at 770 nm, MTSM0077-843-IR, Marktech Optoelectronics Inc.) and an IR PD (maximum sensitivity at 830 nm, SFH2202, ams-OSRAM USA Inc.). A TIA (ADA4505, Analog Devices Inc.) converted the photocurrent from the PD into *V*_PD_. In the power harvesting module, a diode rectifier (BAS4002, Infineon Technologies) and a charge pump (LTC3255IDD, Analog Devices Inc.) were provided for voltage regulation. Tuning capacitors (109 pF) ensured resonance and efficient energy transfer at the operating frequency. A rechargeable Li-Po battery (200 mA·hour; dimensions: 22-mm length, 15-mm width, and 5-mm thickness) provided power to the system [see schematic illustrations and optical microscope images in fig. S1 (A and B)].

### Device assembly

Rhinoceros (V7, McNeel) software served to define the 3D geometries of the clips. Fabrication of the clips and the main body of the device used an SLA 3D printer (Form 3, Formlabs) with a black photocurable resin (Black V4, Formlabs). A cleaning bath (Form Wash, Formlabs) filled with isopropyl alcohol (Thermo Fisher Scientific, US) removed the uncured residual resin from the printed features. A postcuring process at a wavelength of 405 nm involved a 50-min exposure in an ultraviolet curing chamber (Form Cure, Formlabs). The cured clips were released from support pillars as temporary support structures (see photographs in fig. S1C).

The top clip served as a mount for the LED and electronics, secured using an optical adhesive (NOA 63, Edmund Optics). The bottom clip served as a mount for the optical filter (Edmund Optics) and the PD, also secured using an optical adhesive. A flexible wire (SP0013/15, McMaster) electrically interconnected the PD in the bottom clip with the electronics in the top clip. A Li-Po battery was integrated into the bottom clip and electrically interfaced with the electronics housed in the top clip. Two clips were encapsulated with a medical-grade silicone elastomer (Silbione RTV 4420, Elkem) containing a black dye (Slic Pig-black, Smooth-On Inc.) to secure the electronics and the battery while providing an optically isolating enclosure. The torsion springs and metal support were sequentially assembled to provide robust mechanical gripping action to the device [see schematic illustrations in fig. S1 (D to O)].

### Thermal stability

Investigations of the temperature at the surface of the LED used an encapsulated, stand-alone LED clip with a wired setup and an IR camera (FLIR A600, Teledyne FLIR LLC) placed at a distance of 30 cm from the LED. The power supply delivered a voltage of 1.7 V and a current of 30 mA to the LED, similar to the operating conditions of the fluorimeter. The IR camera recorded the temperature at a sampling rate of 3.13 Hz.

### Measurement setup for calibration

A distance of 10 mm between the LED and the PD represented the standard setup. To evaluate both the dynamic range of the device and filter performance, a quartz cuvette (Thermo Fisher Scientific, US) with a thickness of 10 mm was placed between the clips and then filled with DI water. The LED and PD were reliably positioned in opposite orientations against the side wall of the cuvette by the gripping action of the clip mechanism. An optical filter was attached and detached between the PD and the cuvette to compare the received optical power at the PD. Similar studies used an ICG solution at a concentration of 5 μg/ml in place of DI water.

### Device stability via artificial control of the fluorescence solutions

The measurements used a quartz cuvette with a thickness of 10 mm filled with 1.5 ml of an ICG solution at a concentration of 10 μg/ml, with the device clipped against the cuvette wall with the LED and PD facing each other. The reference optical power detected by the filter-covered PD was monitored for 1 min before LED activation, confirming signal stability and the absence of interference from external ambient light under the experimental conditions. Activation of the LED yielded an ICG fluorescence signal, recorded sequentially by stepwise dilution of the ICG solution to reduce the concentrations to 5.0 and 2.5 μg/ml by adding 1.5 and 3.0 ml of DI water, respectively. Subsequently, the ICG solution at 2.5 μg/ml was stepwise increased to 5 and 10 μg/ml by adding 1.0 and 3.5 ml of an ICG solution at a concentration of 20 μg/ml, respectively.

### Fluorescence with ICG, fluorescein, and MB

ICG (SPY AGENT GREEN, Stryker) dissolved in DI water and porcine blood (IGPCWBDF, Innovative Research Inc.) with various concentrations ranging from 0 to 2500 μg/ml served as the measurement solutions for all benchtop characterizations. To investigate the dependence of fluorescence on the concentration of ICG, each ICG solution was pipetted into a 96-well plate (Thermo Fisher Scientific, US) at a volume of 200 μl per well, followed by measurements with (i) an in vivo fluorometric imaging system (IVIS Spectrum, Caliper Life Sciences), (ii) an NIR biomolecular image (Sapphire Imager, Azure Biosystems), (iii) a microplate fluorescence reader (Synergy Neo2, BioTek), and (iv) a device reported here. The excitation/emission settings were (i) 780/820 nm, (ii) 784/832 nm, and (iii) 780/800 to 900 nm. Fluorescence images acquired from the in vivo fluorometric imaging system and the NIR biomolecular imager were processed and quantified into numerical intensity values using associated software (Living Image, version 4.5.2, and Fiji Is Just ImageJ, Fiji, respectively).

To demonstrate the versatility of the platform, fluorescence responses of fluorescein and MB dissolved in DI water at concentrations ranging from 0 to 2000 μg/ml were measured and compared to results from the in vivo fluorometric imaging system and wireless platform. For fluorescence imaging, excitation/emission filter sets of 485/525 nm and 660/685 nm were used for fluorescein and MB, respectively.

### Small-animal studies

All small-animal studies were performed according to the protocol approved by the Wake Forest University (A23-137), and Sprague-Dawley rats (295 to 350 g) were used for planned experiments. Before experimentation, the rats were initially housed in groups under the 12:12-hour light:dark cycle (lights on at 7 a.m.) and kept at ∼25°C, while the relative humidity was maintained between 30 and 70% in the holding room. The rats received water and food ad libitum throughout the experiments, which were conducted during the light cycle (9 a.m. to 2 p.m.). Following transfer of the animals from the holding room to the laboratory with a single animal per cage, the animals were deeply anesthetized with ketamine and xylazine according to standard dosing protocols. Hair on the rat body, specifically over both hindlimbs, was shaved using a portable clipper, followed by skin sterilization with betadine and ethanol swabs. A 20G catheter (BD) was inserted into the jugular vein via a ventral neck incision and secured with a nonabsorbable suture to allow subsequent administration of fluorescent material. Before the experiment was initiated, heparin was administered subcutaneously at a dose of 100 IU/kg for anticoagulation. All rats used in this study were naïve and immunocompetent, with no previous experimental history.

### Ischemia-reperfusion study

The animal was placed in an in vivo fluorometric imager (Pearl Trilogy, LICOR Biosciences), a research-grade preclinical imaging instrument, for sequential dual-channel near-infrared fluorescence image acquisition, followed by device mounting on both hind paws and the tail. ICG injections with a dose of 2.5 mg/kg were performed three times within a single experimental protocol to monitor different physiological rhythms in the baseline, ischemic, and reperfusion phases. Skin incision was made on the right hindlimb, followed by dissection of the perivascular tissue, to expose the femoral artery and vein for subsequent clamping. Vascular clamping was performed immediately before the second ICG injection using microvascular clamps (jaw dimensions: 5.5 mm by 1.5 mm; length: 11 mm; stainless steel; S&T AG) to induce ischemia in the right hindlimb. Reperfusion was initiated by releasing the clamps immediately before the third ICG injection. After monitoring the reperfusion phase with the third ICG injection, the protocol ended. All three phases (baseline, ischemia, and reperfusion) were maintained for 30 min to monitor decreases in fluorescence signals with hepatic metabolism and blood perfusion. During the entire protocol, the system wirelessly monitored fluorescence responses, continuously displayed signals on the user interface in real time, and automatically stored data, while the standard fluorescence imaging system discretely captured images at intervals of 90 to 120 s.

### Hindlimb amputation-replantation

Before hindlimb amputation, the first ICG injection with a dose of 2.5 mg/kg was given to jugular vein to monitor baseline fluorescence signals in both hindlimbs. The skin and muscle tissues of the left groin were sequentially transected using surgical scissors and a bipolar electrocautery (Hyfrecator 2000, CONMED) while preserving the femoral artery and vein. The femoral bone was then transected using a high-speed rotary drill (Dremel 8200, Dremel). The femoral artery and vein were dissected, clamped, and transected to complete the hindlimb amputation. To prevent blood clot formation within the vessels, vascular flushing was performed using heparinized saline. After full amputation, the second ICG injection was carried out to compare the signals from both devices attached to each hindlimb. As the first step in replantation, osteosynthesis was performed to reconnect the transected femoral bones using an intramedullary pin (stainless steel; 18G needle). Muscle tissues were repaired with a 4-0 polyglactin 910 suture (J392H, Ethicon). Microvascular anastomoses of the femoral artery and vein reconnected the transected blood vessels using a monofilament 10-0 polypropylene suture (T05A10N10-13, AROS Surgical Instruments). Reperfusion was initiated by releasing the clamps, followed by skin closure using a 4-0 polyglactin 910 suture (J392H, Ethicon). The animal was then placed on the imaging bed of the fluorometric imager. The third ICG injection was performed in this step to monitor postoperative blood circulation. The wireless fluorimeter system continuously monitored fluorescence dynamics and displayed the signals on the user interface in real time throughout the entire protocol.

### Large-animal studies

All large-animal studies were performed according to the protocol approved by the Northwestern Institutional Animal Care and Use Committee (IS00019051). The pig breeds are Yucatan pig, and they were purchased from an approved vendor (Sinclair BioResources). This study used male and female Yucatan pigs (*n* = 3; 99.8, 85.0, and 80.00 kg), and veterinary staff examined all animals before the study. Before experimentation, the pigs were initially housed in an Association for Assessment and Accreditation of Laboratory Animal Care–accredited facility under the 12:12-hour light:dark cycle and kept at ∼25°C, while the relative humidity was maintained between 30 and 70% in the holding room. The animals were monitored daily by trained staff and fed a commercial pig diet of pig feed twice daily throughout the experiments, which were conducted during the light cycle (8 a.m. to 2 p.m.). The water was available ad libitum. Before initiation of the experimental protocol, the animals were deeply anesthetized under general anesthesia with tiletamine-zolazepam (Telazol; 100 mg/ml; 6 mg/kg; intramuscular), according to standard dosing protocols. The animals were then transferred from the holding room to the laboratory, where they were endotracheally intubated and anesthesia was maintained with isoflurane in 100% oxygen in the laboratory.

To facilitate subsequent injection of the ICG dye solution, an incision on the neck was performed to access the indwelling intravenous line. The wireless and wired fluorimeter systems were then securely mounted on ears and lip of the pig to monitor the fluorescence signals. An external smart device wirelessly monitored fluorescence responses from the device, whereas electrical signals from the wired fluorimeter were recorded using a readout software (LabChart Pro, ADInstruments) during the entire protocol. Heparinized saline (10 cm^3^) was administered for flushing. The ICG at a concentration of 2.25 mg/ml and a dose of 0.15 mg/kg was injected intravenously through an indwelling central venous line within the venae cavae. During the protocol, blood samples following ICG injection were drawn every 30 s for the first 5 min, every 1 min for the subsequent 15 min, and every 2 min for the final 10 min via the arterial line. The fluorescence intensities of blood samples drawn from the pigs were measured immediately after completion of the protocol using a microplate fluorescence reader (Synergy Neo2, BioTek). These measurements served as the ground truth for the electrical data obtained from the device.

### Data collection and analysis

A BLE-enabled smart device running a custom Android application (Galaxy tab S6, Galaxy S8, S9, and S10) wirelessly collected, continuously displayed, and automatically stored fluorescence signals for all measurements, including those associated with device calibration; with measurement of ICG, fluorescein, and MB materials; with device stability; and with small- and large-animal studies. Data were analyzed by MATLAB R2024b, Microsoft Excel, and Origin 2018.

### Optical simulations

The Monte Carlo method was used to simulate light propagation. For simulations involving the tail, ear, paw, and finger, the same procedures as those used in the benchtop simulation were followed, except that the optical parameters and geometries were modified accordingly. These cases are discussed individually in the following sections.

The rat tail was modeled as a cylindrical domain with a diameter of 7 mm, on the basis of experimental measurements, and an axial length assumed to be much larger than the region of interest. The rat paw was modeled as a slab with a thickness of 4.7 mm, also on the basis of experimental data, while its in-plane dimensions are assumed to be effectively infinite. Because the wavelength-dependent absorption coefficient $\mu_{a}\left(\lambda \right)$ and reduced scattering coefficient $\mu_{s}^{\prime }\left(\lambda \right)$ for the tail and paw are not available in the literature, reported optical properties of mouse skin in the 750- to 860-nm range (*50*) were used as reference values. The overall absorption coefficient is expressed as $\mu_{a}\left(\lambda \right)=\mu_{a,\text{tissue}}\left(\lambda \right)+c_{\text{ICG}}\varepsilon_{a,\text{ICG}}\left(\lambda \right)$, where $\mu_{a,\text{tissue}}\left(\lambda \right)$ corresponds to the baseline absorption of the tail or paw tissue.

The pig ear was modeled as a homogeneous slab with a thickness of 5 mm, while its lateral dimensions in the x and y directions were assumed to be much larger than the simulation domain. The wavelength-dependent absorption coefficient $\mu_{a,\text{ear}}$ and reduced scattering coefficient $\mu_{s}^{\prime }$ were taken from a previous study (*51*). The overall absorption coefficient was calculated using the same formulation as above, $\mu_{a}\left(\lambda \right)=\mu_{a,\text{ear}}\left(\lambda \right)+c_{\text{ICG}}\varepsilon_{a,\text{ICG}}\left(\lambda \right)$.

The geometry of the finger (fig. S53) was adopted from here (*52*, *53*), consisting of four components: skin, fat, muscle, and bone. The skin was further divided into six layers (table S2), each characterized by a specific thickness, water volume fraction $V_{w}$, blood volume fraction $V_{b}$, and melanin volume fraction $V_{m}$. The wavelength-dependent absorption coefficient for each skin layer is given by

$$\begin{array}{c} \mu_{a}\left(\lambda \right)=V_{w}\mu_{a,w}+V_{b}\mu_{a,b}+V_{m}\mu_{a,m}+\\ \left(1-V_{w}-V_{b}-V_{m}\right)\mu_{a,\text{base}}+c_{\text{ICG}}\varepsilon_{a,\text{ICG}} \end{array}$$ (1)

where $\mu_{a,w}$, $\mu_{a,b}$, and $\mu_{a,m}$ are absorption coefficients of pure water, blood, and melanin, respectively. The values of $\mu_{a,w}$ and $\mu_{a,b}$ are tabulated from previous work (*41*, *54*). Melanin absorption was modeled as $\mu_{a,m}=6.6\times 10^{10}\lambda^{-3.33}\;{\text{mm}}^{-1}$ (*55*), and the baseline absorption coefficient in the absence of a chromophore is given by $\mu_{a,\text{base}}=7.84\times 10^{7}\lambda^{-3.255}\;{\text{mm}}^{-1}$ (*55*). The reduced scattering coefficient for all six skin layers is $4.8\left[0.409{\left(\frac{\lambda }{500\;\text{nm}}\right)}^{-4}+0.591{\left(\frac{\lambda }{500\;\text{nm}}\right)}^{-0.702}\right]\;{\text{mm}}^{-1}$ (*56*).

The $\mu_{a}$ and $\mu_{s}^{\prime }$ for fat, muscle, and bone were taken from published studies (*56*–*59*).

## Supplementary Materials

### This PDF file includes:

Notes S1 to S9 Figs. S1 to S55 Tables S1 to S4 References
