# Supplementary material for: A compact, wireless fluorimeter system for continuous, real-time monitoring of tissue perfusion via in vivo fluorescence signals
Source: Sci Adv. 2026 Sep 18;12(38):eaeg8187. doi: 10.1126/sciadv.aeg8187 (PMC13588188; doi:10.1126/sciadv.aeg8187)
Supplement: Supplementary file 1 [file sciadv.aeg8187_sm.pdf]

Science Advances
AAAS

Supplementary Materials for

A compact, wireless fluorimeter system for continuous, real-time monitoring of tissue perfusion via in vivo fluorescence signals

Tae Wan Park *et al.*

Corresponding author: Yonggang Huang, y-huang@northwestern.edu; John A. Rogers, jrogers@northwestern.edu

Sci. Adv. **12**, eaeg8187 (2026)
DOI: 10.1126/sciadv.aeg8187

This PDF file includes:

Supplementary Notes S1 to S9
Figs. S1 to S55
Tables S1 to S4
References

Supplementary Note S1. Electromagnetic power harvesting module for wireless charging.

A flexible printed circuit board (fPCB) supports a receiver (RX) coil, which enables wireless charging of a lithium-polymer (Li-Po) battery as a harvesting unit, and provides electrical interconnections between optical sensing (OS) and Bluetooth Low Energy (BLE) system-on-a-chip (SoC) modules. Wireless battery charging system occurs via power transfer at 13.56 MHz through inductive coupling between an external transmission (TX) coil and the RX coil. This operating frequency is widely used for near-field communication (N0046C) and is considered safe for human exposure, making it suitable for biomedical applications (*60, 61*). The oscillating voltage at the RX coil is rectified using a bridge diode and subsequently boosted to 4.12 V via a charge pump to support charging of the Li-Po battery. The resonance frequency of the RX coil during wireless charging in the encapsulated wireless fluorimeter system (WIFS) is tuned by capacitors (109 pF).

Supplementary Note S2. Photophysical factors for quantitative interpretation of *in vivo* fluorescence signals

Quantitative relationship between electrical signal and quantum yield

The measured electrical signal is directly proportional to the light intensity collected by the detector. Since the fluorescence intensity is proportional to the quantum yield, and the LED light is independent of the quantum yield, the electrical signal increases linearly with the quantum yield. The simulation results show the received intensity as a function of quantum yield in **Fig. S11**.

Effects of reabsorption, scattering, and concentration

Reabsorption and scattering are critical. These factors are explicitly accounted for using Monte Carlo (MC) simulations. The relationship between fluorescence intensity and ICG concentration

is non-monotonic. As concentration increases, the signal initially rises due to higher absorption. However, at higher concentrations, reabsorption shielding dominates, causing the signal to decrease. This trend is consistent across our benchtop tests and the additional ear-tissue simulation, confirming that our sensing strategy can capture these complex optical interactions in biological media.

Influence of Microenvironmental Effects (Protein binding & aggregation)

In blood, ICG binding typically increases the fluorescence quantum yield and causes a red-shift in the emission peak compared to aqueous solutions. Both effects increase the amount of fluorescence light can be detected by the sensor (*42, 62, 63*). At high local concentrations or in specific physiological conditions, ICG molecules may aggregate leading to fluorescence quenching and a reduction in quantum yield.

Supplementary Note S3. Compatibility with various fluorophores

All experimental procedures and protocols were approved by Northewstern University Institutional Animal Care and Use Committee (IS00024323). This study used adult Sprague-Dawley rats (Charles River Laboratory) with weights of 291.3 g and 321.2 g. Prior to demonstration, the rats were initially housed in group under 12:12-hour light:dark (LD) cycle (lights on at 7 am) and kept at approximately 25°C, while the relative humidity maintained between 30 to 70% in the holding room. Anestesia was induced and maintained with isoflurane delivered in oxygen. Hairs over the chest and both hindlimbs were removed using a commercial hair remover (Nair™ Baby Oil Body Cream, Church & Dwight Co., Inc.), followed by skin sterilization with betadine and ethanol swabs. Far-field electrocardiogram (ECG) signals were recorded throughout the entire protocol using PowerLab 4/26, PowerLab FE234 Quad Bio Amp, and LabChart ECG

Recording software (ADInstruments) with three electrodes placed on the chest and both hindlimbs of the rats. The ECG data were collected by sampling rate of 512 Hz.

To enable fluorophore administration, the jugular vein was exposed through a ventral neck incision, followed by insertion and fixation of an indwelling catheter using nonabsorbable suture using 5-0 silk retraction suture. A heparin (100 IU/kg) was injected into the rat for anticoagulation. The device mounted on the right hindpaw and started recording for the confirmation of the baseline signals, followed by injection of MB and fluorescein solutions at a concentration of 2.5 mg/mL through the ventral catheter. The dose of each fluorophore was 2.5 mg/kg. During the entire protocol, the device continuously monitored fluorescence responses, wirelessly displayed signals on the user interface in real time, and automatically stored data. The fluorescence kinetics were successfully observed after injection of fluorophores, as shown in **Fig. S21**. Normal cardiac rhythms were maintained regardless of MB and fluorescein injections. These results verify that the wireless fluorimeter system reported here is compatible not only with ICG but also with other fluorophores. At the end of the experiment, the animals were euthanized with the approved method of euthanasia. All rats used in this study were healthy, immunocompetent, and no previous experimental history.

Supplementary Note S4. Injection dose of ICG in rodent and porcine models.

Since the fluorescence-concentration curve of ICG exhibits non-monotonic behavior, which is attributed to reabsorption and self-quenching effects at high concentrations (*64*), optimization of the injection dose is required to monitor the reliable fluorescence dynamics.

Rodent models

An ICG dose of 2.5 mg/kg was administered to rat models to operate to the initial low-concentration regime, where fluorescence intensity increases monotonically and approximately linearly with ICG concentration. Since the blood volume in rodents is approximately 7~7.84% of their body weight (*65-67*), an ICG dose of 2.5 mg/kg was administered to rat models (295-330 g), corresponding to an estimated concentration below 20 μg/mL assuming complete systemic distribution.

Porcine models

Based on the successful demonstration of kinetic tracking following ICG administration in the rat model, a substantially lower dose of 0.15 mg/kg was injected to the porcine models to better reflect clinically relevant conditions and demonstrate its translational applicability. Using a blood volume corresponding to about 6~7% of body weight in porcine model (*68-70*), the resulting ICG concentration is estimated to be below 2.5 μg/mL.

The selected dose was intended to place the estimated systemic ICG concentration within the increasing approximately linear area of the experimentally measured ICG fluorescence-concentration curve, while avoiding the high-concentration quenching area. Therefore, the device operation in *in vivo* fluorescence monitoring were designed to account for this non-linear response rather than assuming a linear fluorescence-concentration relationship.

Supplementary Note S5. Long-term stability of excitation source.

The stability of excitation source is a key factor in fluorescence sensing based on optical approach. A spectrometer (FLAME-S-VIS-NIR-ES, Ocean Optics Inc.) equipped with an optical fiber (QR400-1-UV-VIS, Ocean Optics Inc.) served to characterize the long-term stability of the NIR LED, the main excitation source in this study. Measurement involved positioning the optical fiber

and LED linearly in a 180° alignment and continuously monitoring the radiant efficiency over 2 h. The optical power density was calculated by integrating the wavelength-dependent spectral radiant efficiency profile over the measured spectral range and normalizing by the illuminated area according to

$$E = \frac{1}{A}\int_{\lambda_{min}}^{\lambda_{max}} P_{\lambda}(\lambda)d\lambda$$

where E, A, and $P_{\lambda}(\lambda)$ are the optical power density, illuminated area, and spectral optical power density as a function of wavelength, respectively. The variation in optical output power was below 1.7% over the entire measurement. This fluctuation is substantially smaller than the signal changes resulting from variations in ICG concentration. A voltage-mode driving scheme using an LDO regulator was selected to optimize the device for long-term wearable monitoring by enabling miniaturization and low power consumption. Furthermore, the LDO-based system provides a low-noise voltage source, maintaining the high signal-to-noise ratio (SNR) that is essential in biochemical sensing (*71*).

Supplementary Note S6. Concentration-voltage quantification.

The wireless fluorimeter reported here can detect fluorescence signals from ICG solutions over a wide range of concentrations from 0 to 2500 μg/mL, convert them into electrical signals, and display them as quantitative values.

To quantify ICG concentration from the raw voltage readouts measured *in vivo* experiments, blood samples drawn during real-time measurement compare with benchtop-prepared samples with known concentrations, which provide calibrated voltage-concentration relationships (**Fig. S40**). First, to mimic the experimental conditions of the porcine ear with a thickness of 5 mm, benchtop quantification involves measuring ICG in blood samples of known concentrations in a quartz

cuvette with a thickness of 5 mm using the wireless fluorimeter (**Fig. S40A**). Owing to the design of the ICG dosing strategy to operate within the low-concentration monotonic regime (see **Supplementary Note S4**), the concentration-voltage relationship exhibits a linear dependence. Second, following ICG injection in the porcine model, blood sampling performed at 30, 60, and 120 s intervals after the fluorescence signal reached its peak value. These drawn blood samples enable direct comparison with benchtop results using the same measurement setup. Red dashed line in **Fig. S40** denotes peak fluorescence signal in this study, showing strong agreement between readout from *in vivo* measurement (2.20 μg/mL) and simulation results (2.17 μg/mL). In addition, the fluorescence dynamics in the *in vivo* measurement closely matched with those in the drawn blood samples, while exhibiting a 36.1% reduction due to the tissue attenuation. Collectively, after applying a correction factor of ~1.565, the fluorescence signals measured through the porcine ear with a thickness of 5 mm correspond to the calibration equation in **Fig. S40A**, enabling quantitative estimation of ICG concentration.

Supplementary Note S7. Sensitivity analysis

To account for physiological variations such as blood perfusion, hydration levels, and melanin content, we performed a sensitivity analysis using the porcine ear tissue model. The baseline absorption and reduced scattering coefficients values were varied by 20%. Although the absolute fluorescence intensity fluctuates with changes in tissue optical properties, the signal ratio before and after ICG injection remains nearly constant, because both the LED and fluorescence intensities change in the same direction as the tissue optical properties vary (**Fig. S45**).

Supplementary Note S8. On-body test with human participants

The experiments for tests of motion artifact resistance and device calibration for human use were approved by the Institutional Review Board of Northwestern University (STU00220375). All participants provided informed consents prior to the tests.

Device calibration for human use

The measurements involved mounting the device to the index fingers of five participants and to five fingers of a single participant with varying thicknesses (Thumb, 15 mm; index, middle, and ring fingers, 13 mm; little finger, 11 mm). Five participants fully inserted their index fingers into the device configured with a thickness of 13 mm, showing highly consistent baseline signals (**Fig. S48A**). Thickness-dependent baseline signal acquisition consisted of sequentially securing the device to the fingers, activating the system using a smart device, and monitoring the signals in real time (**Fig. S48B**). The participants remained stationary during the protocol.

Resistance for motion artifacts

The measurement involved mounting the wireless fluorimeter to the index finger of the participant with a thickness of 13 mm, attaching motion sensors to the chest and dorsal hand, and allowing free movement. The finger clip device provided secure mounting on the finger of participant (**Fig. S51A**). The motion sensors on the chest and dorsal hand, simultaneously capturing inertial measurement unit (IMU) data and cardiac sound intensity (CSI). During the protocol, the participant walked on level ground and ascended and descended stairs. Throughout these activities, the baseline signal maintained stability, demonstrating reliable operation under dynamic conditions (**Fig. S51**).

Supplementary Note S9. ***Ex vivo*** **demonstration of fluorescence sensing with human heart.**

Donor human heart rejected for organ transplant was procured from the Gift of Hope Organ & Tissue Donor Network as deidentified discarded tissue not involving human individuals. The tissue was maintained in University of Wisconsin cardioplegic solution in the cold box utilizing specialized transport. Prior to initiation of the experimental protocol, the heart was then transferred to a closed-loop *ex vivo* perfusion system with a customized tissue chamber (17 cm × 17 cm × 6 cm) and was perfused with a modified Tyrode's solution (128.2 mM NaCl, 4.7 mM KCl, 1.05 mM $MgCl_2$, 1.3 mM $CaCl_2$, 1.19 mM NaH_2PO_4, 20 mM $NaHCO_3$, 11.1 mM glucose) with a volume of 4 L and bubbled with 95% O_2/5% CO_2. The pressure of the heart was maintained between 80 mmHg and 120 mm Hg by regulating the perfusion flow rate of 253 mL/min through tubing-based peristaltic pump (Masterflex™ L/S™ Precision Variable-Speed Console Drive, Avantor). The right and left coronary ostia were cannulated separately with the coronary cannulas to support the physiologic perfusion conditions, and the outlet was drained through the coronary sinus directly into the chamber. The system incorporated a dedicated injection port for reliable ICG administration into the perfusion line.

To measure the fluorescence perfusion in whole human heart, the tailored clip design was engineered (**Fig. S54**). The device configuration embedded the PD clip within the heart tissue and placed the LED clip on the surface of the heart, allowing the two parts to face each other across the conus branch of the right coronary artery in the pulmonary artery of the heart (**Fig. S55A-B**). The clips were secured inside and onto the heart surfaces using non-absorbable monofilament 6-0 polypropylene suture (Ethicon, 8705H). The signal acquisition from PD used a smart device while independently driving the LED using an external power source. ICG at a concentration of 2.5 mg/mL was then injected into the 4L Tyrode's solution with a volume of 2 mL through the port in

the line, resulting in a rapid increase in fluorescence intensity. After monitoring the saturated fluorescence curve over 30 min, an additional ICG with a volume of 0.5 mL was injected, resulting in subsequent increase in fluorescence intensity (**Fig. S55C**). The experimental setup lacked blood perfusion and liver metabolism, resulting in no observable signal decrease over time. The *ex vivo* demonstration highlights successful perfusion monitoring using the wireless fluorimeter platform reported here in a whole human heart.

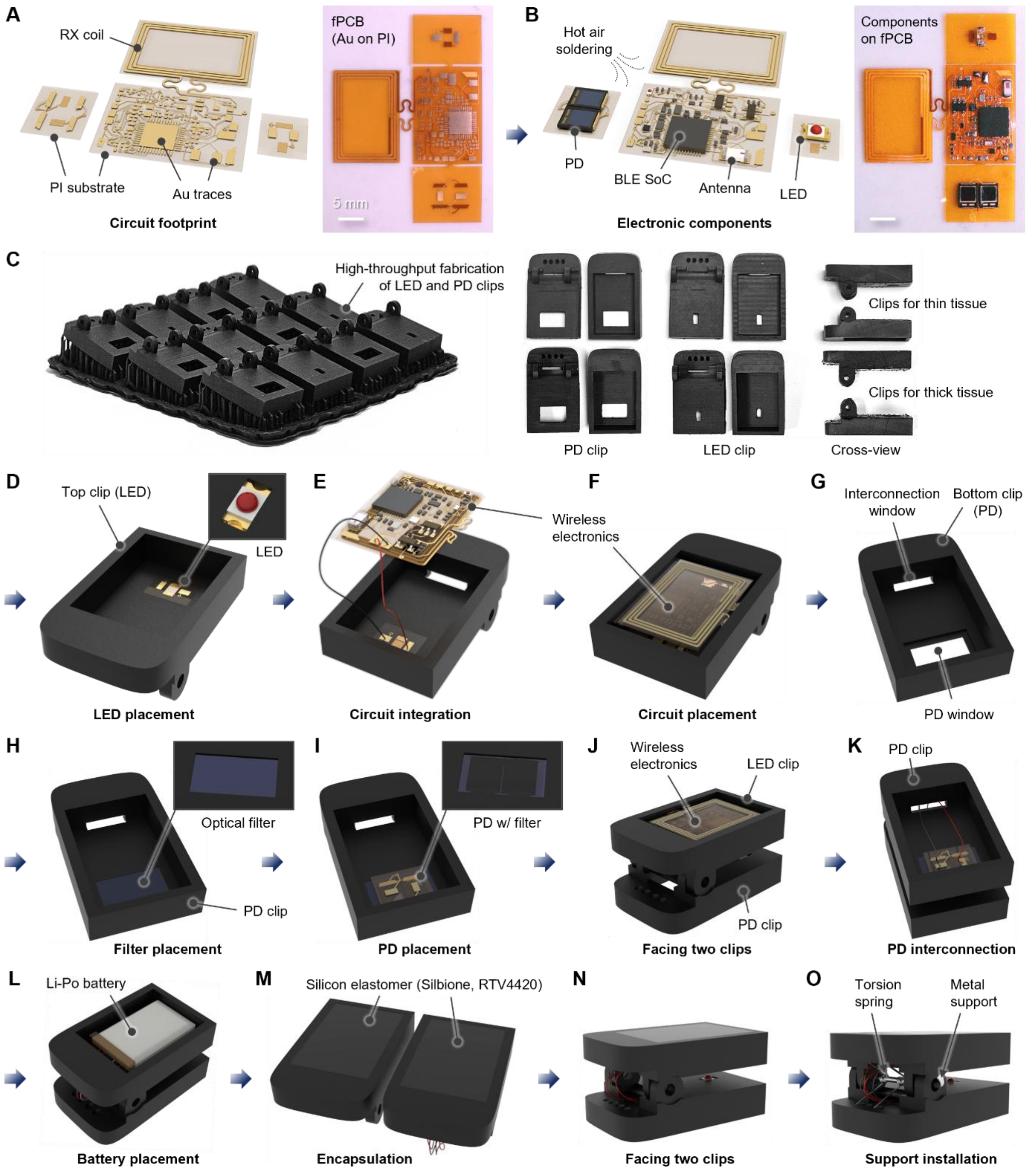

Fig. S1. Device fabrication and assembly procedures. **(A)** fPCB circuit footprint. **(B)** Soldering electronic components. **(C)** Clip fabrication *via* additive manufacturing. **(D)** LED placement on top clip fabricated by SLA additive manufacturing. **(E)** Circuit integration with LED. **(F)** Circuit placement in LED clip. **(G)** Preparation of bottom clip for PD. **(H)** Optical filter placement in PD clip. **(I)** PD placement. **(J)** Facing two clips. **(K)** PD interconnection with wireless electronics in the LED clip. **(L)** Battery placement and interconnection with wireless electronics. **(M)** Encapsulation of LED and PD clips. **(N)** Facing two encapsulated clips. **(O)** Device assembly with support installation to provide gripping functionality.

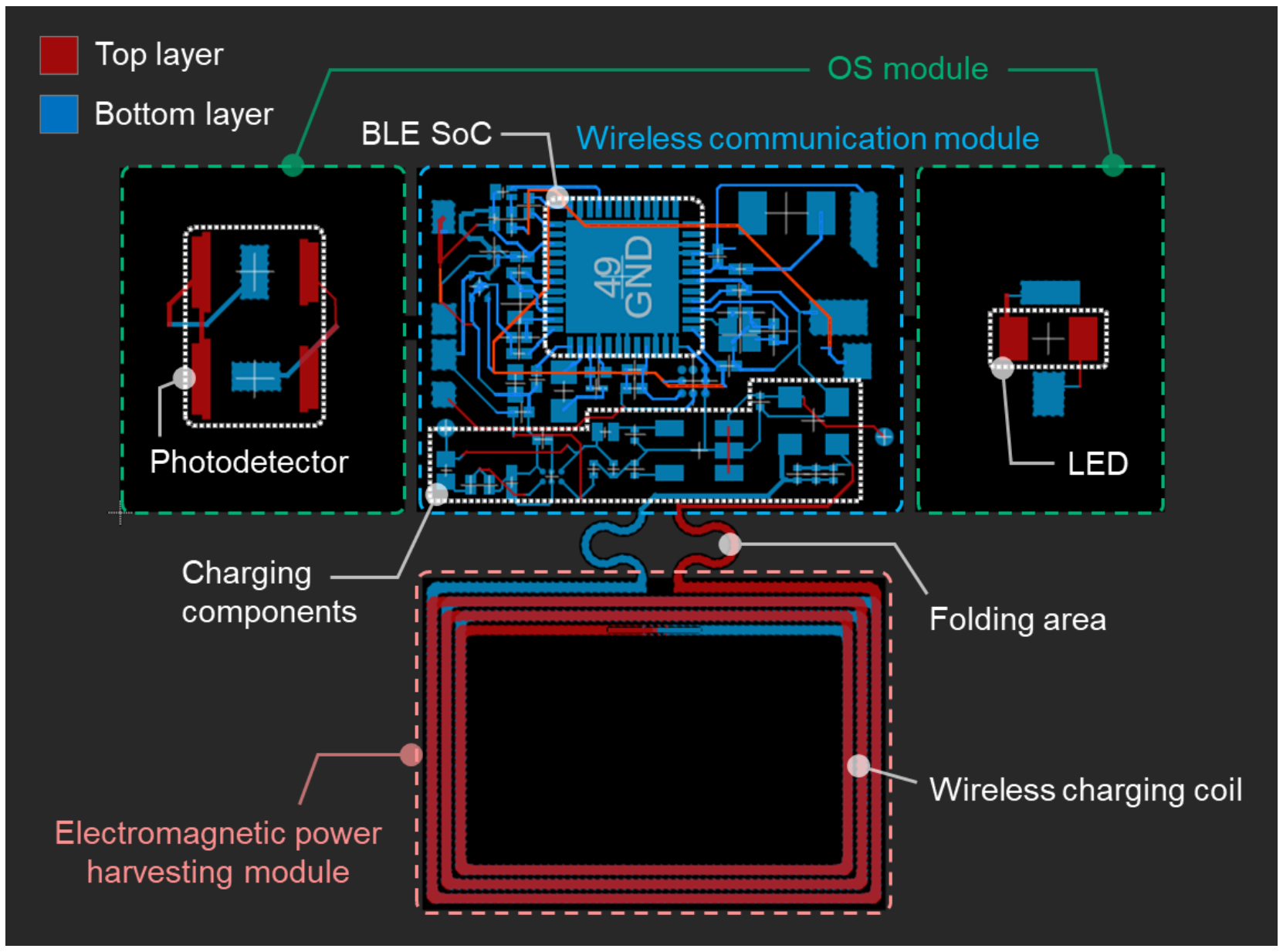

Fig. S2. Layout of the flexible printed circuit board. Annotated board layout showing the OS module (green dashed boxes), wireless communication module (blue dashed box), and electromagnetic power harvesting module (pink dashed box).

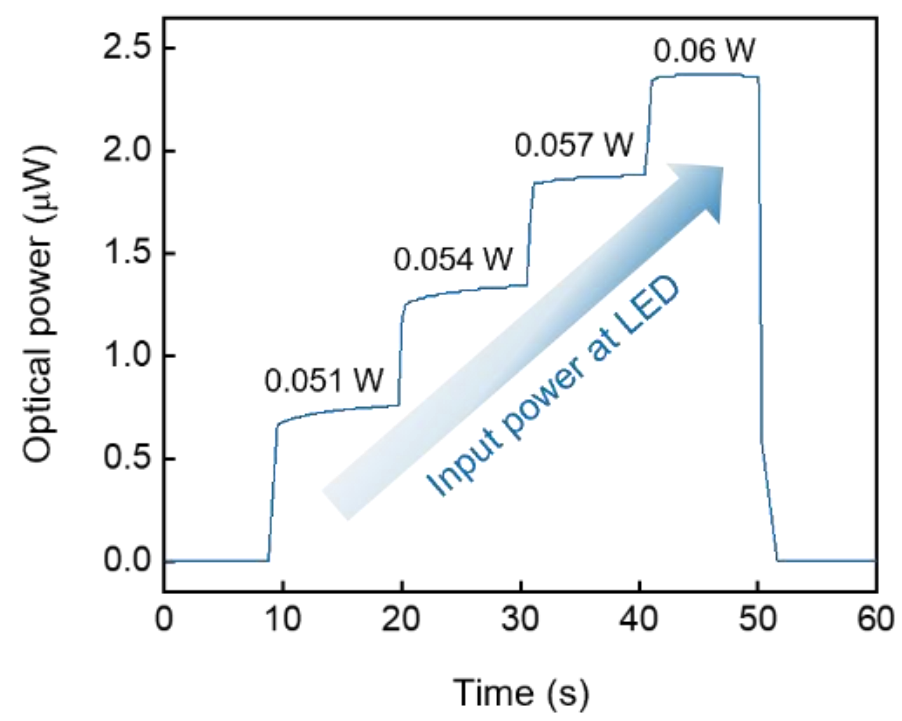

Fig. S3. Relationship between the applied electrical power and the optical output power of the NIR LED. The optical emission power profile of the NIR LED measured during periods of changes in the input power from 0 to 0.06 W using an Optical Power Meter (PM100D, ThorLabs). Each period lasts 10 seconds with the same input power as a power supplier. The optical power response follows the applied power levels, demonstrating controllable, reversible optical output during cyclic operation.

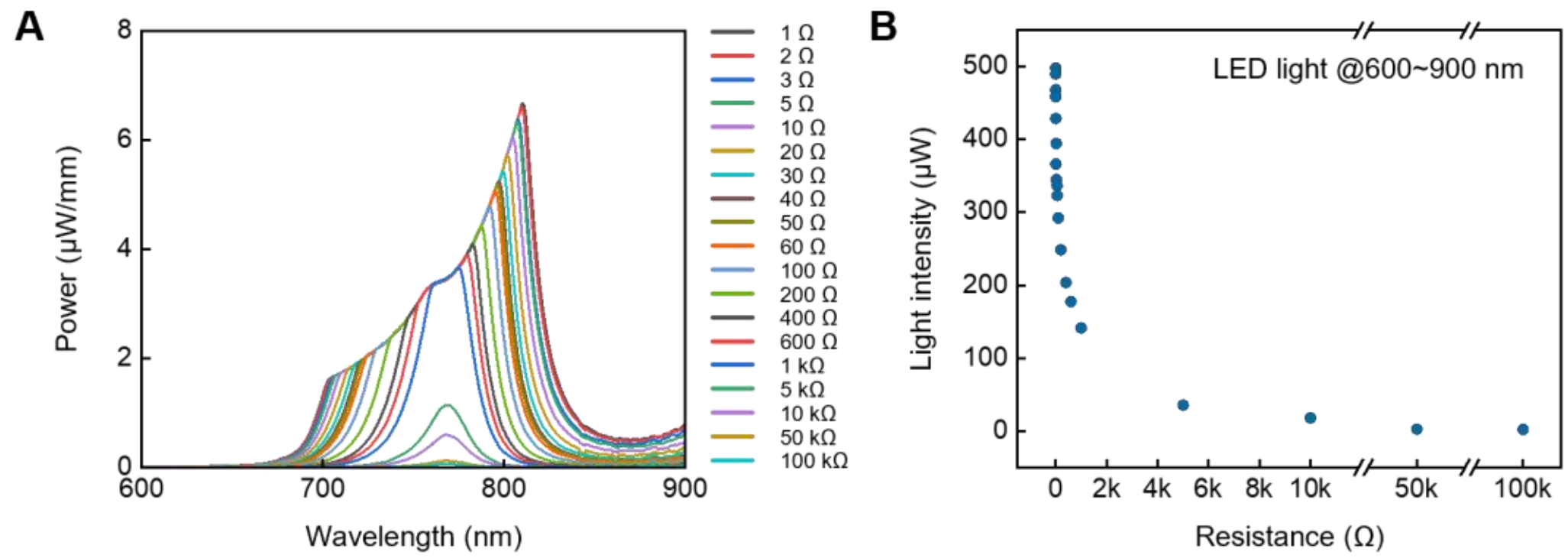

Fig. S4. Characteristics of optical power from the NIR LED as a function of resistance. (A) Emission power spectra of the NIR LED measured under different resistance conditions, estimated by using an Ocean Optics spectrometer. Changes in spectral power intensity reflect differences in driving currents, which are controlled by the resistance at 1.7V supplied by a power supplier. **(B)** Integrated light intensity calculated by integrating the emission power spectra over the wavelength range from 600 to 900 nm as a function of resistance, demonstrating a tunability of the NIR LED optical emission power.

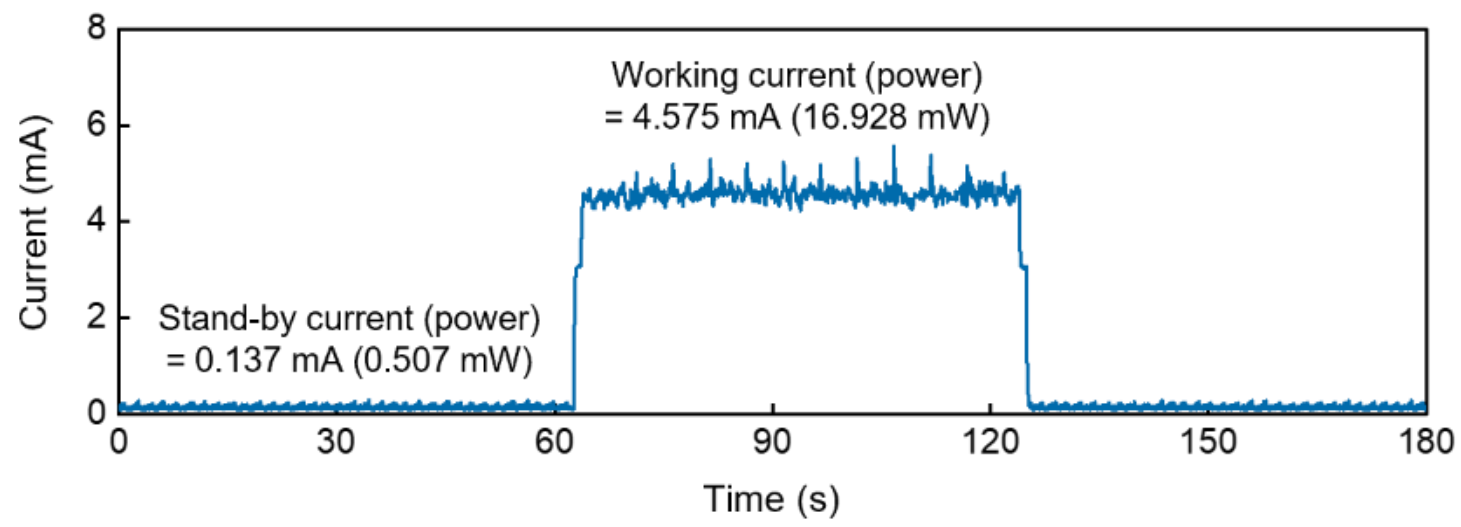

Fig. S5. Power consumption of the wireless fluorimeter. Current consumption profile of wireless fluorimeter system during standby and operation modes.

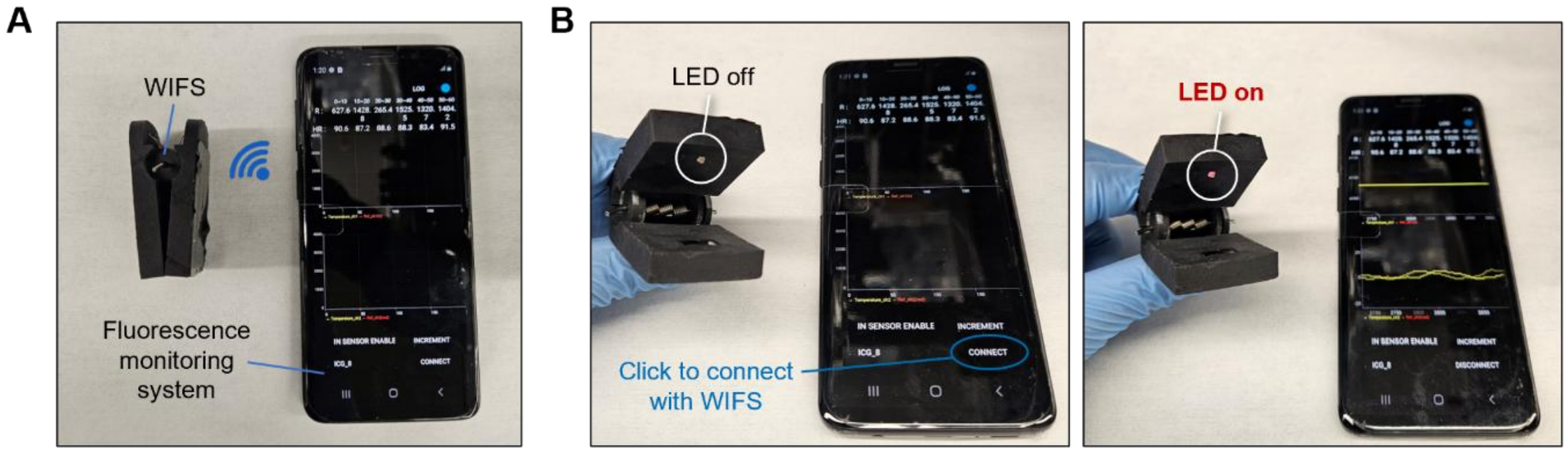

Fig. S6. Wireless fluorimeter and fluorescence monitoring system. (A) Photograph of the wireless fluorimeter system and a smart device to monitor the fluorescence signal. **(B)** Device operation. The LED in the wireless fluorimeter automatically actives upon pressing the connect button in the monitoring system.

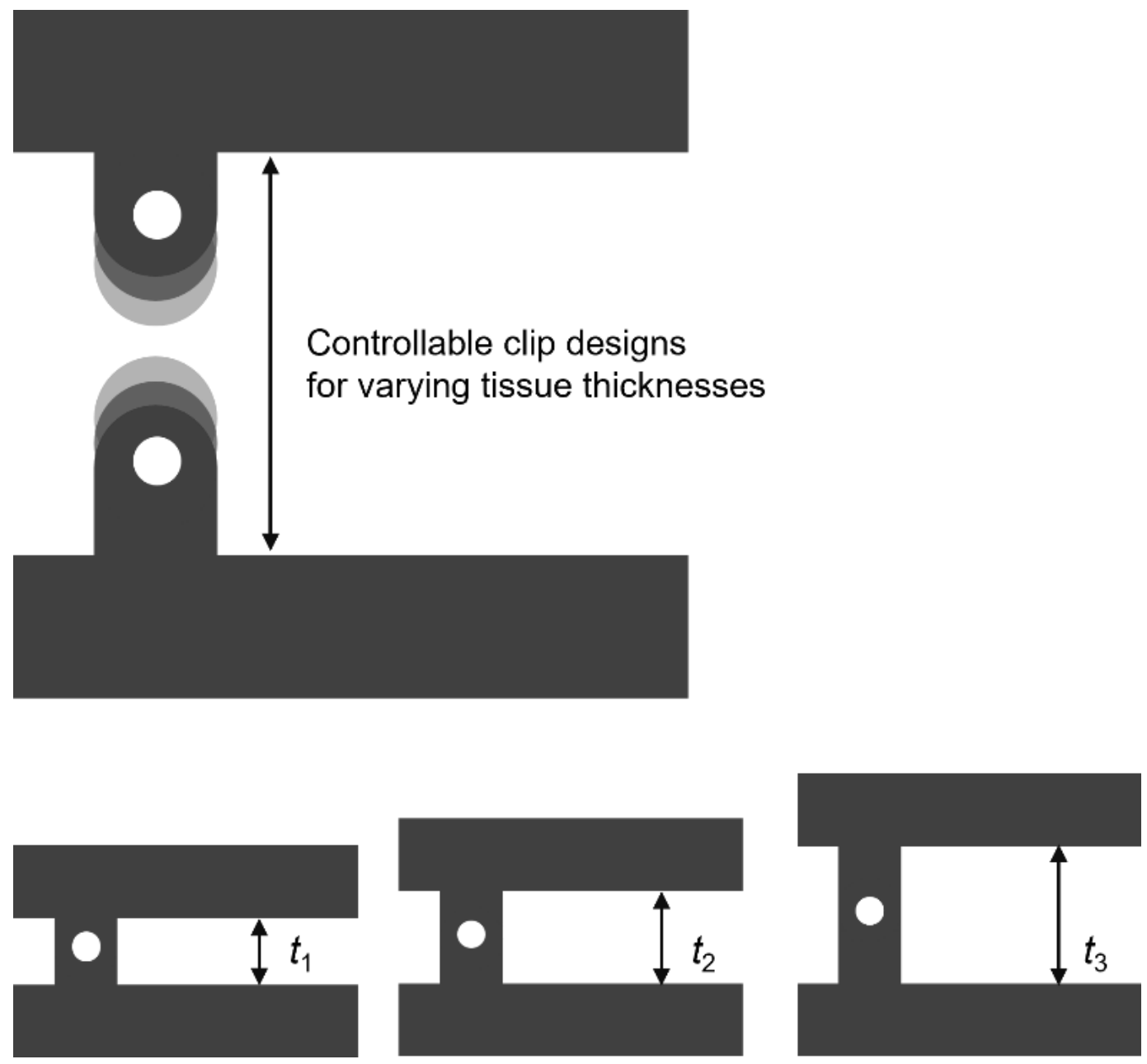

Fig. S7. Schematic illustrations of mechanical clip, designed for tissues with different thicknesses.

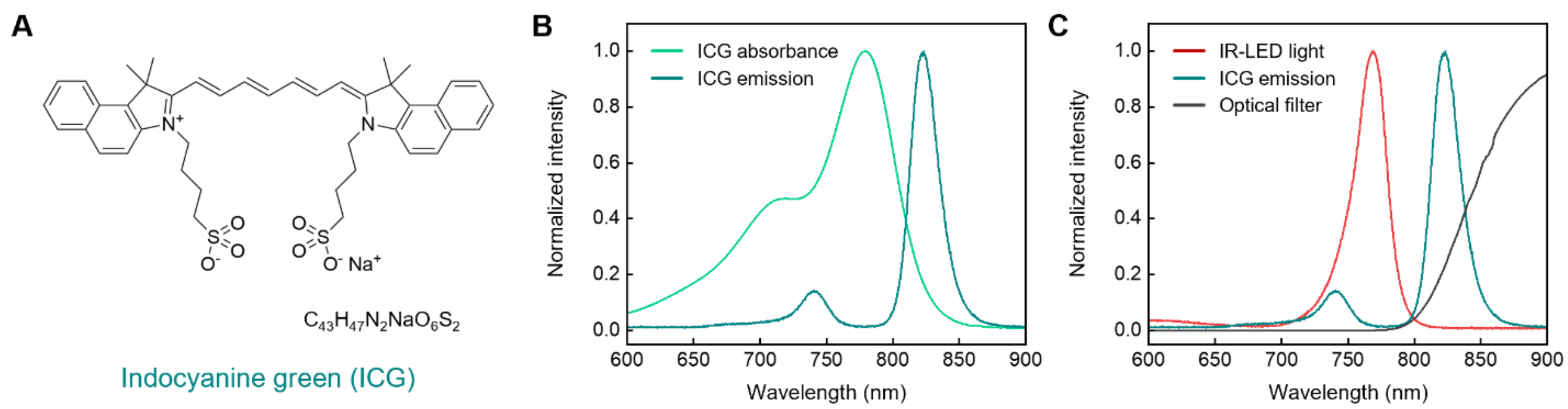

Fig. S8. Fluorescence sensing strategy for ICG. (A) Chemical structure of ICG. **(B)** Absorbance and emission profiles of ICG. **(C)** Optimization for successful ICG sensing. An optical filter (black curve) successfully blocks an NIR LED light (red curve) and selectively passes an ICG emission light.

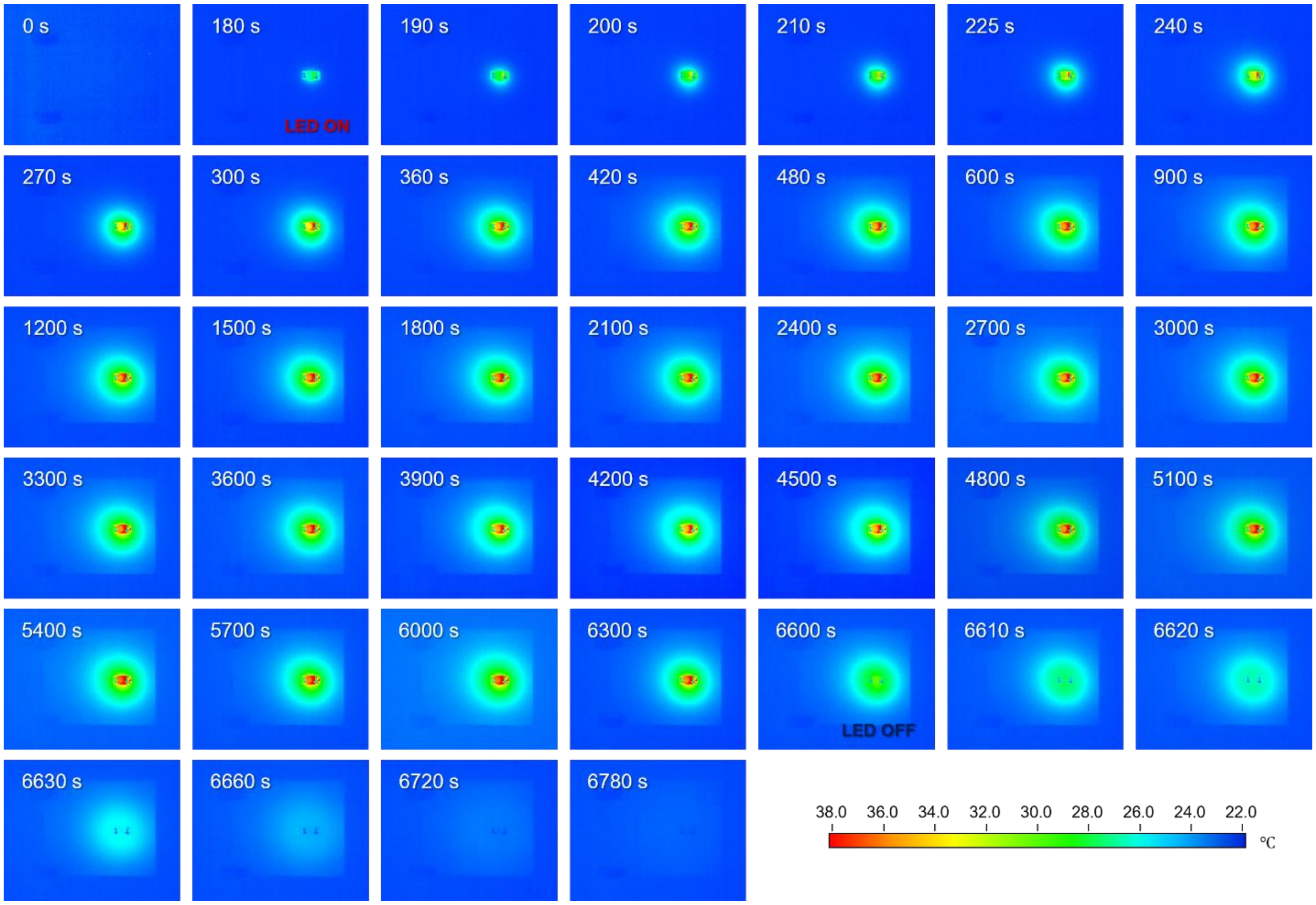

Fig. S9. Temperatures on the surface of the device during long-term operation as measured by an IR camera. Maximum temperature of 37.1℃, compliant with medical safety standards for skin contact on animal and human use.

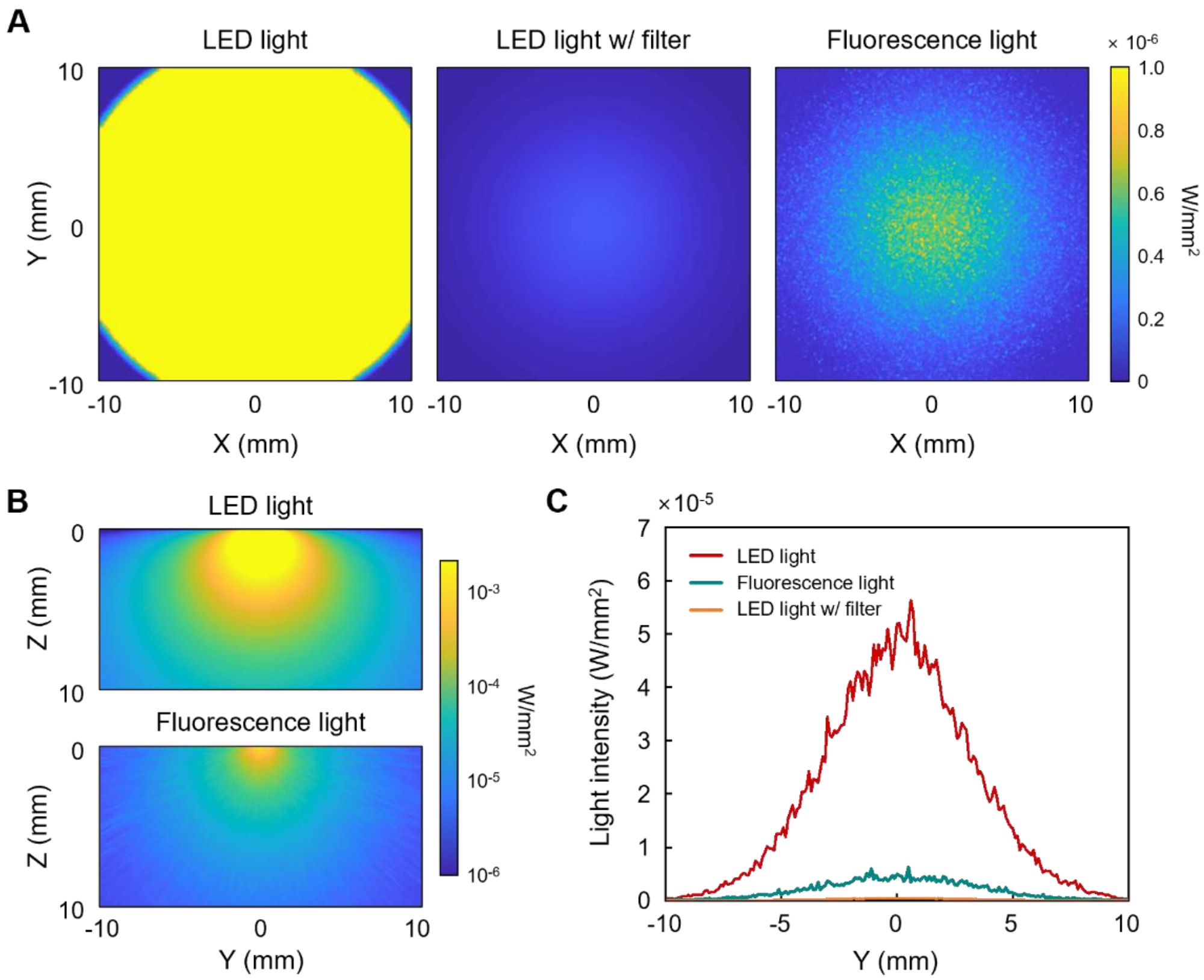

Fig. S10. Monte Carlo simulation of light intensity distributions. (A) Light intensity distribution at the bottom surface. **(B)** Light intensity distribution in a cross-sectional plane. **(C)** Light intensity distribution at the bottom surface along $X = 0$.

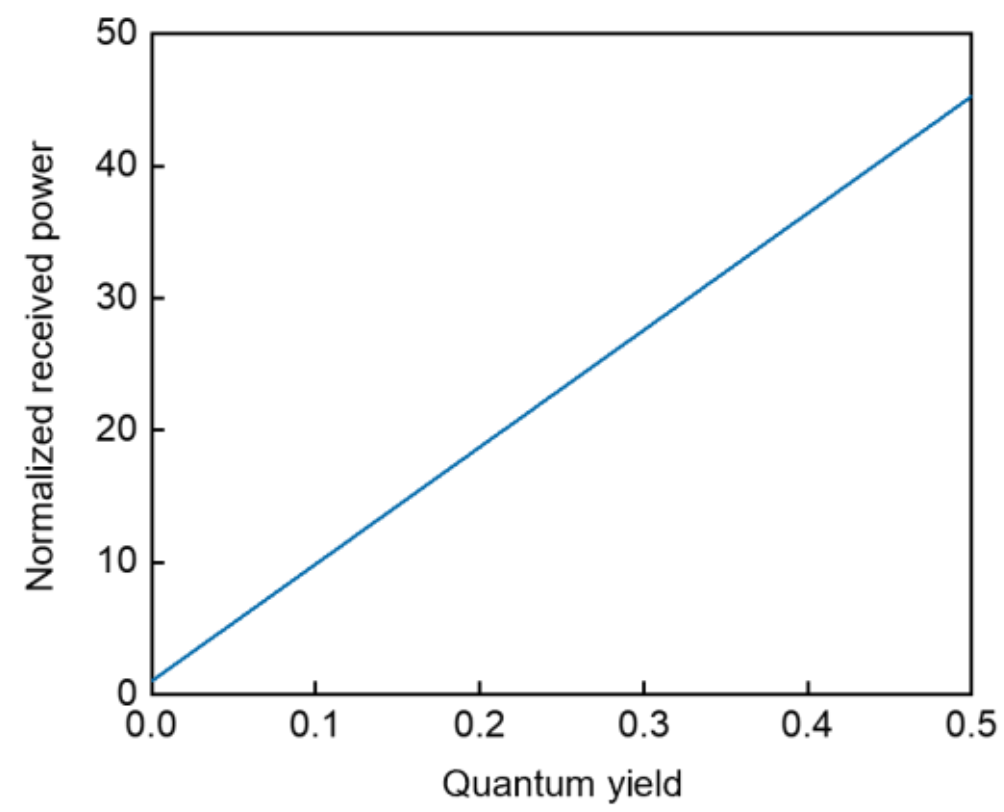

Fig. S11. Linear relationship between the normalized received power (normalized to unity at zero quantum yield) and quantum yield.

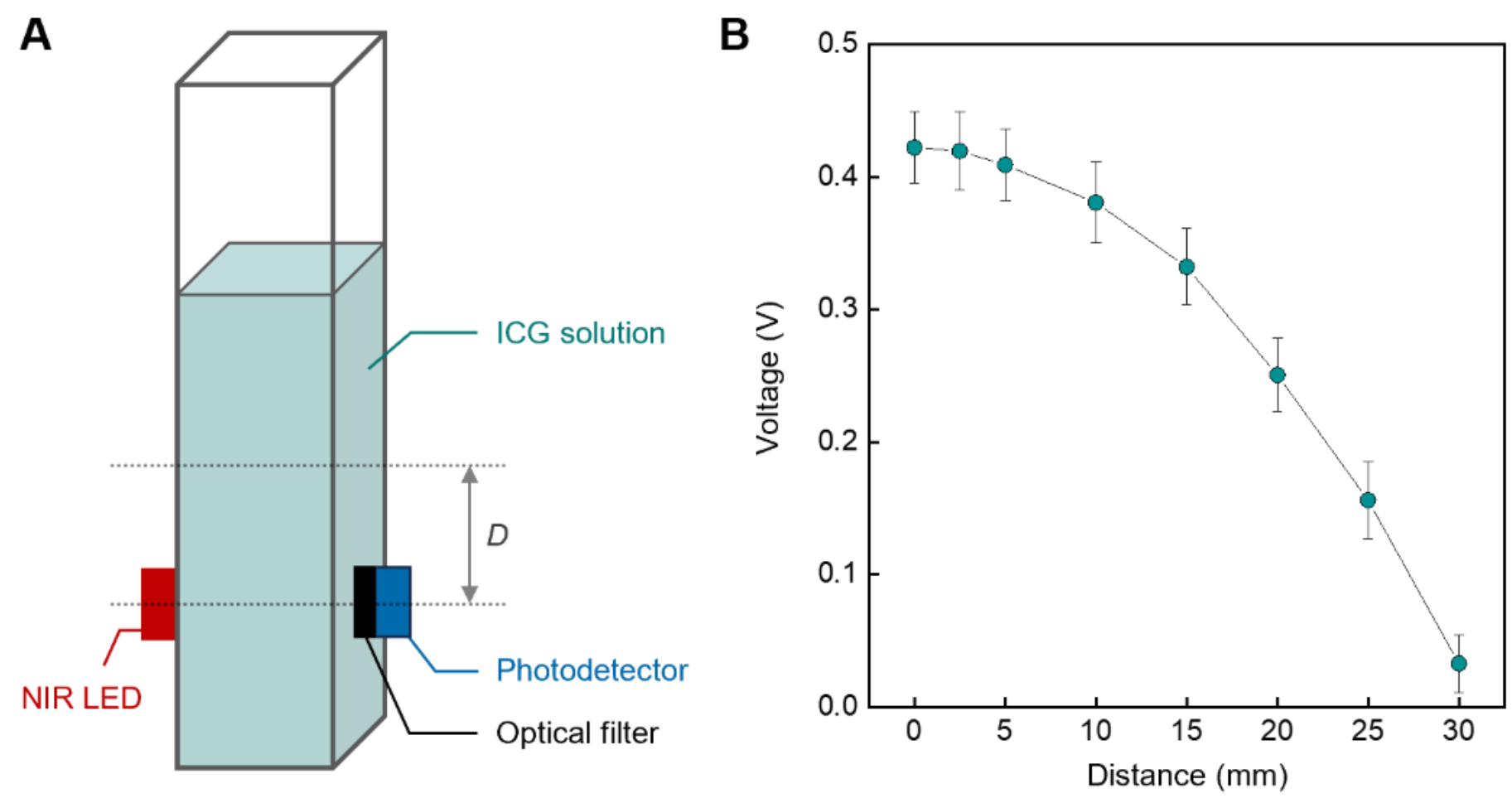

Fig. S12. Effect of optical misalignment on fluorescence readouts. (A) Schematic illustration of the experimental setup. **(B)** Fluorescence readouts with varying distance (*D*) between the NIR LED and photodetector, showing a decrease in signal with increasing *D*.

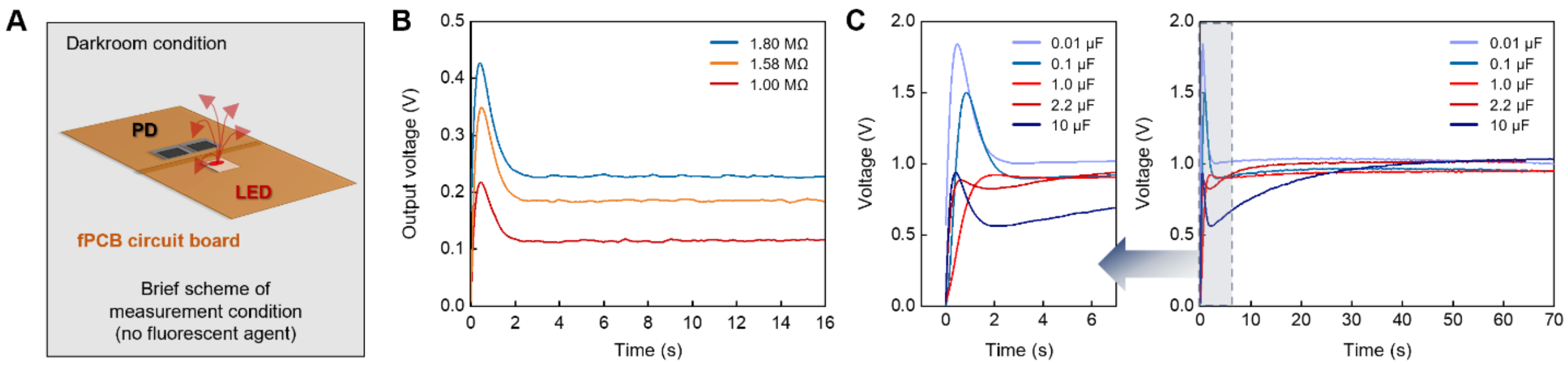

Fig. S13. Device sensitivity and response speed. (A) Schematic illustration of the response measurement setup. **(B)** Response signal amplitude as a function of the resistance values in the opamp. **(C)** Response speeds and control using capacitors. All measurements were conducted under dark room conditions.

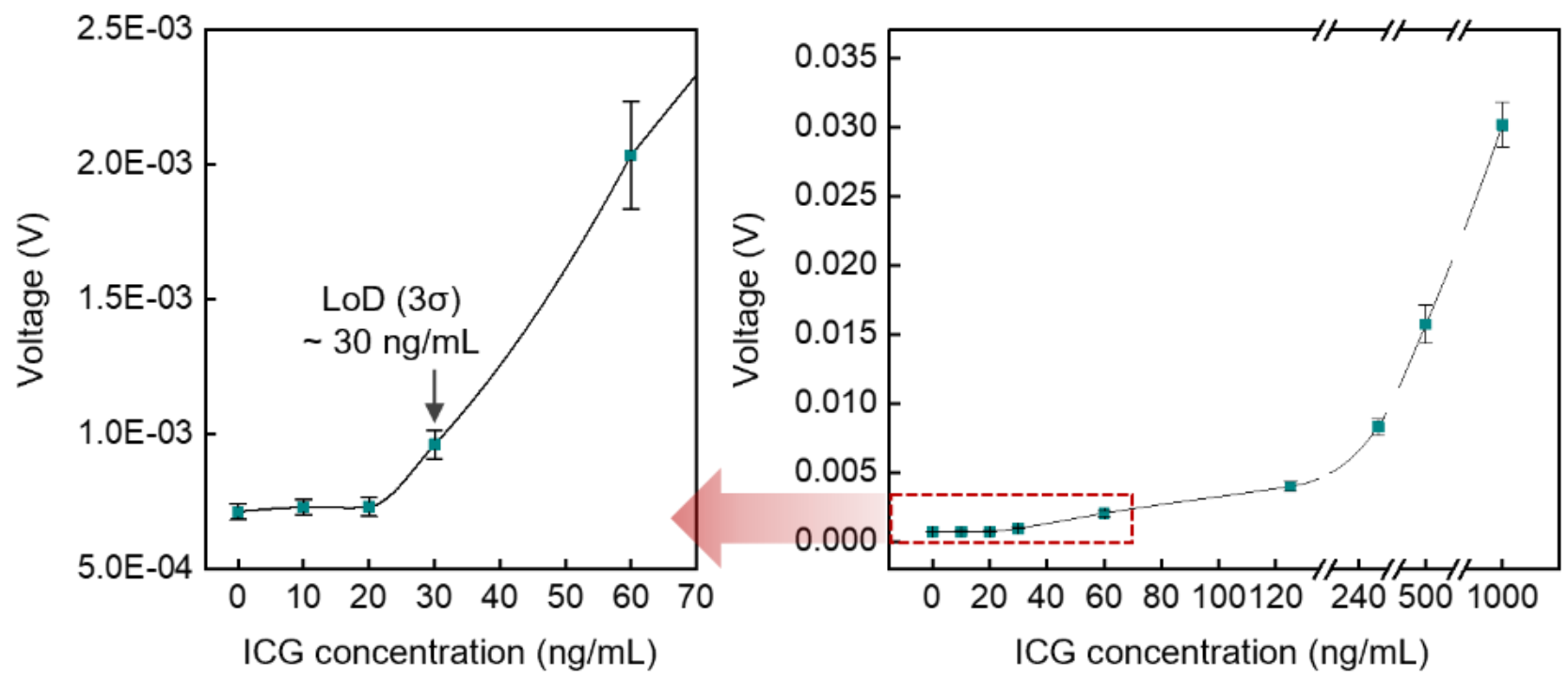

Fig. S14. LOD of the wireless fluorimeter system. The standard curve was generated by polynomial fitting. The wireless fluorimeter reported here detects ICG concentrations down to 30 ng/mL.

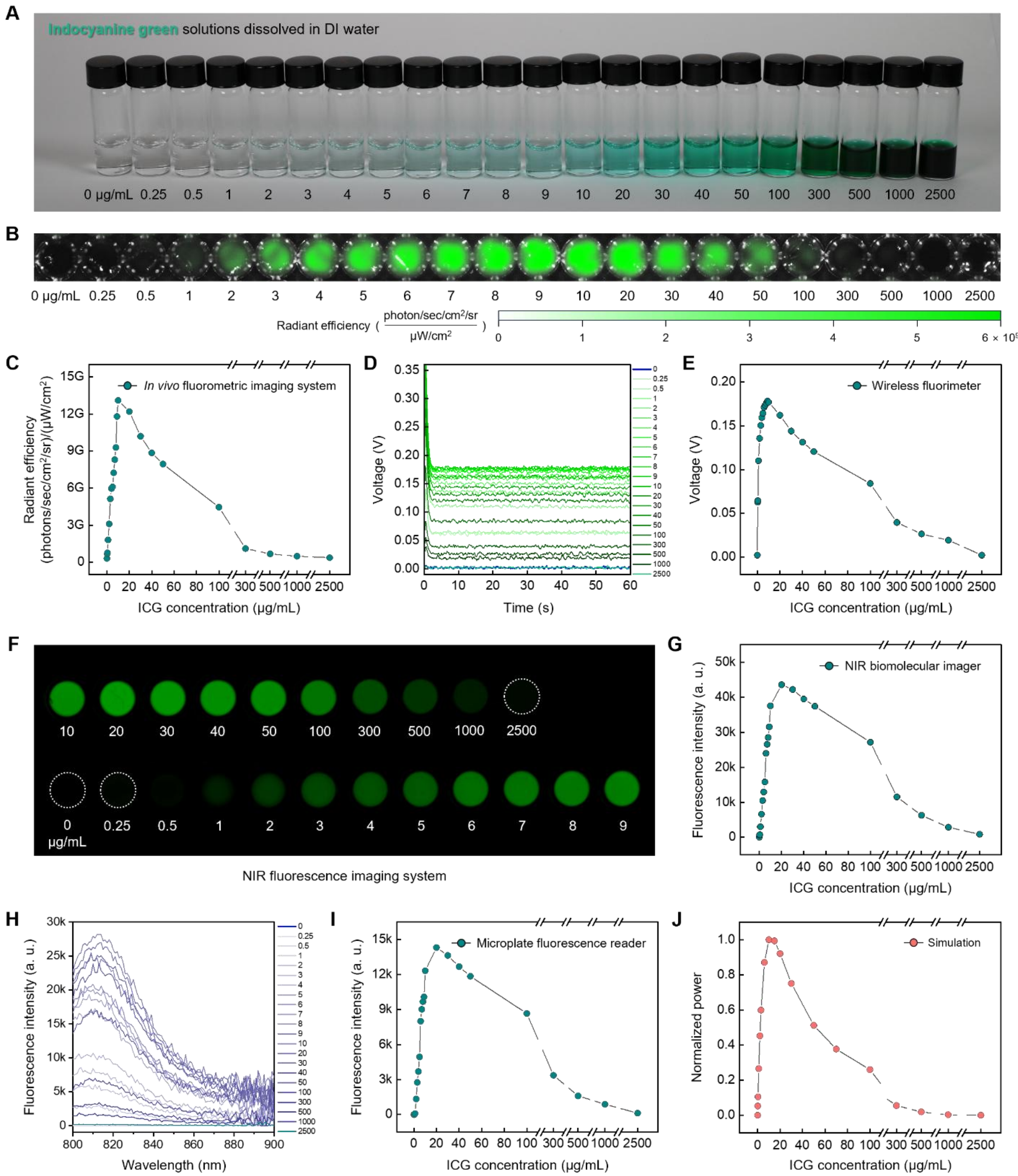

Fig. S15. Fluorescence sensing characteristics of ICG dissolved in DI water. (A) Photograph of ICG solutions dissolved in DI water with various concentrations from 0 to 2,500 µg/mL. ICG fluorescence signals captured by **(B, C)** *in vivo* fluorometric imaging system, **(D, E)** wireless fluorimeter, **(F, G)** NIR fluorescence imaging system, and **(H, I)** microplate fluorescence reader instrument. All measurements show strong agreement with **(J)** Monte Carlo simulation results.

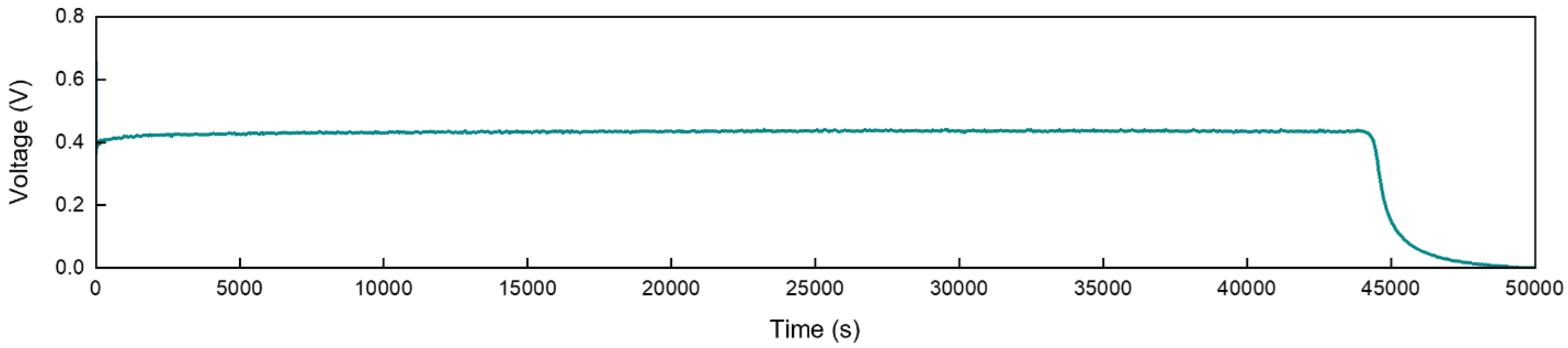

Fig. S16. Feasibility study of continuous real-time monitoring of fluorescence signal with a wireless fluorimeter. The concentration of ICG solution was maintained at 2.5 μg/mL during the long-term measurement.

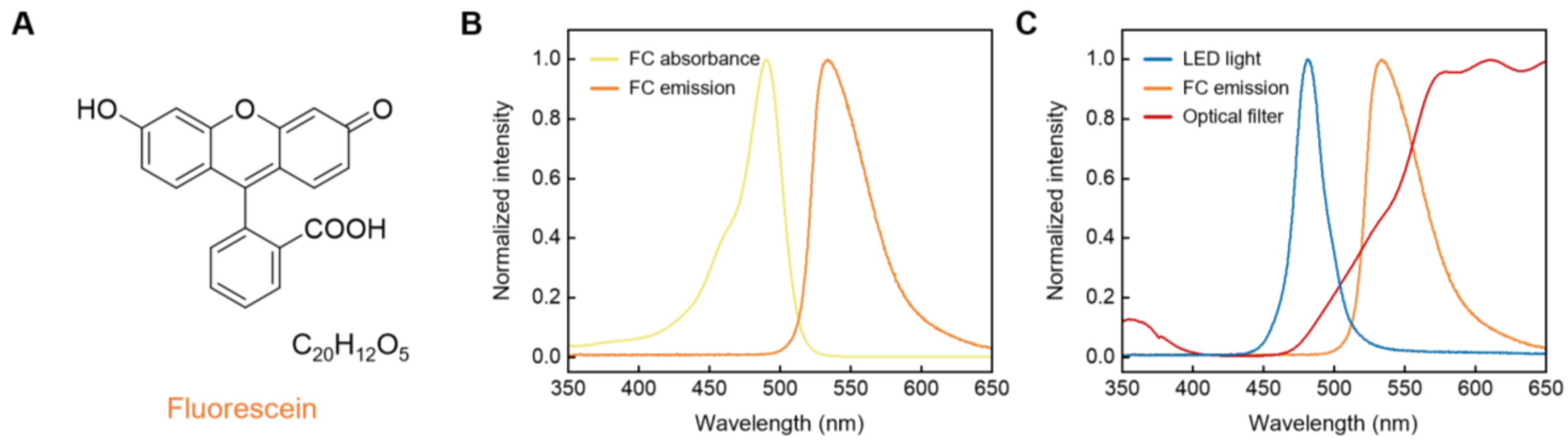

Fig. S17. Fluorescence sensing strategy for fluorescein. (A) Chemical structure and **(B)** absorbance and emission profiles of fluorescein. **(C)** Optimization for successful sensing of fluorescein. An optical filter (red) effectively blocks a blue LED light (blue) and selectively passes a fluorescein emission light (orange).

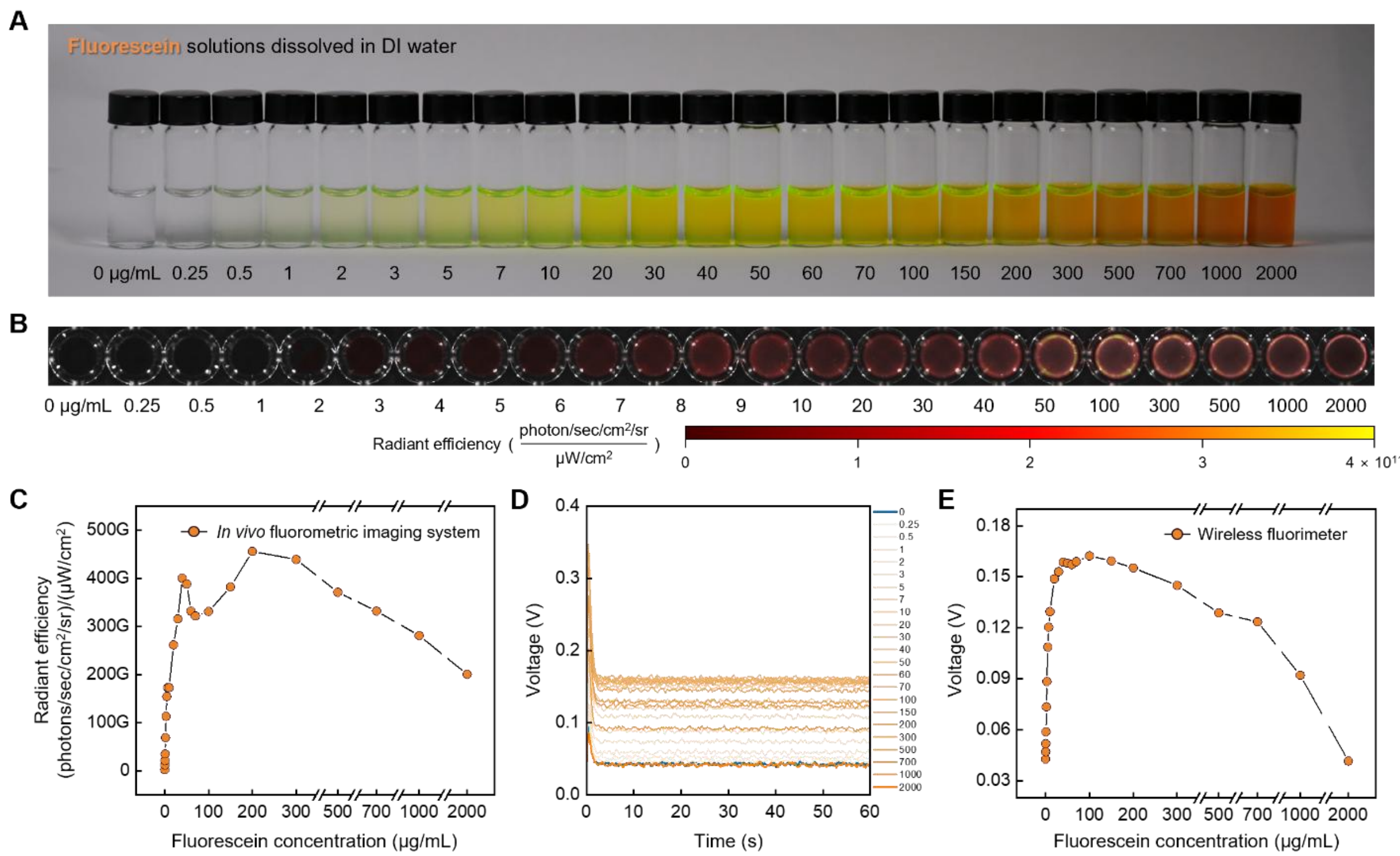

Fig. S18. Fluorescence sensing of fluorescein dissolved in DI water. (A) Photograph of fluorescein solutions with various concentrations from 0 to 2,000 μg/mL. Fluorescence signals captured by **(B, C)** *in vivo* fluorometric imaging system and **(D, E)** wireless fluorimeter.

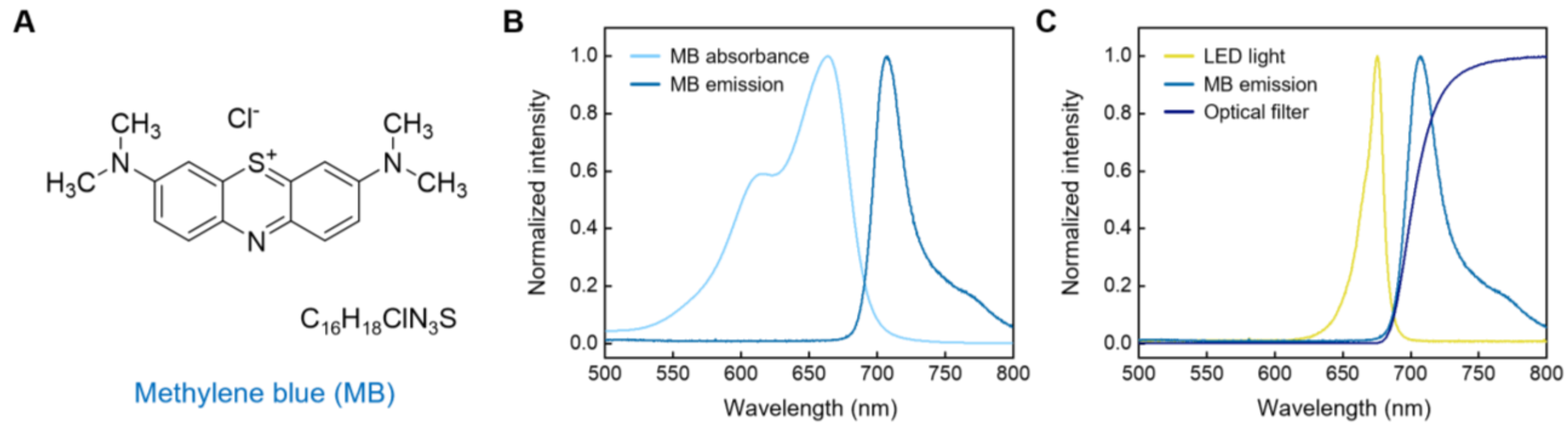

Fig. S19. Fluorescence sensing strategy for MB. (A) Chemical structure and **(B)** absorbance and emission profiles of MB. **(C)** Optimization for successful sensing of MB. An optical filter (dark blue) systematically blocks a yellow LED light (yellow) and selectively passes a MB emission light (light blue).

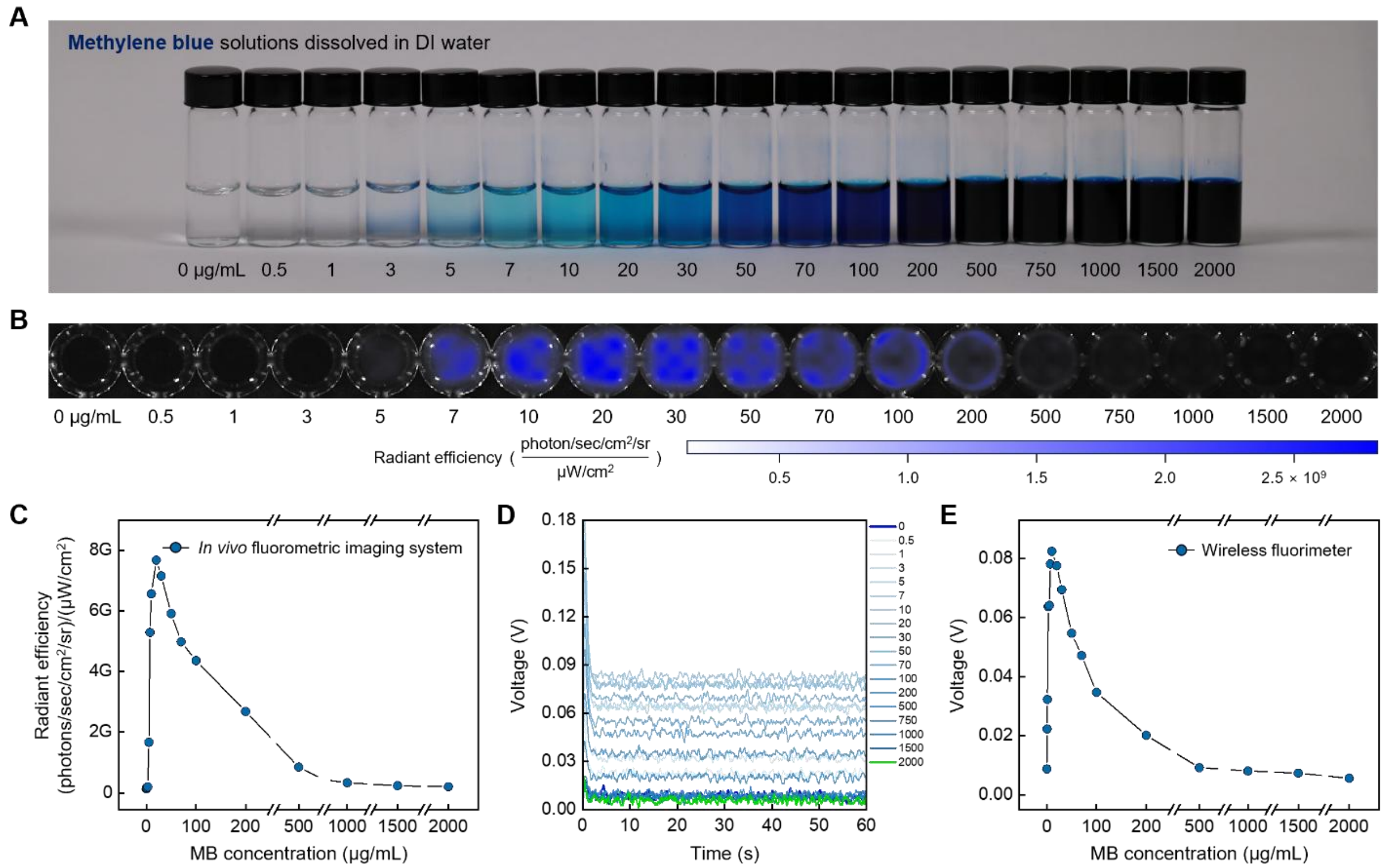

Fig. S20. Fluorescence sensing of MB dissolved in DI water. (A) Photograph of MB solutions dissolved in DI water with various concentrations from 0 to 2,000 μg/mL. MB fluorescence signals captured by **(B, C)** *in vivo* fluorometric imaging system and **(D, E)** wireless fluorimeter.

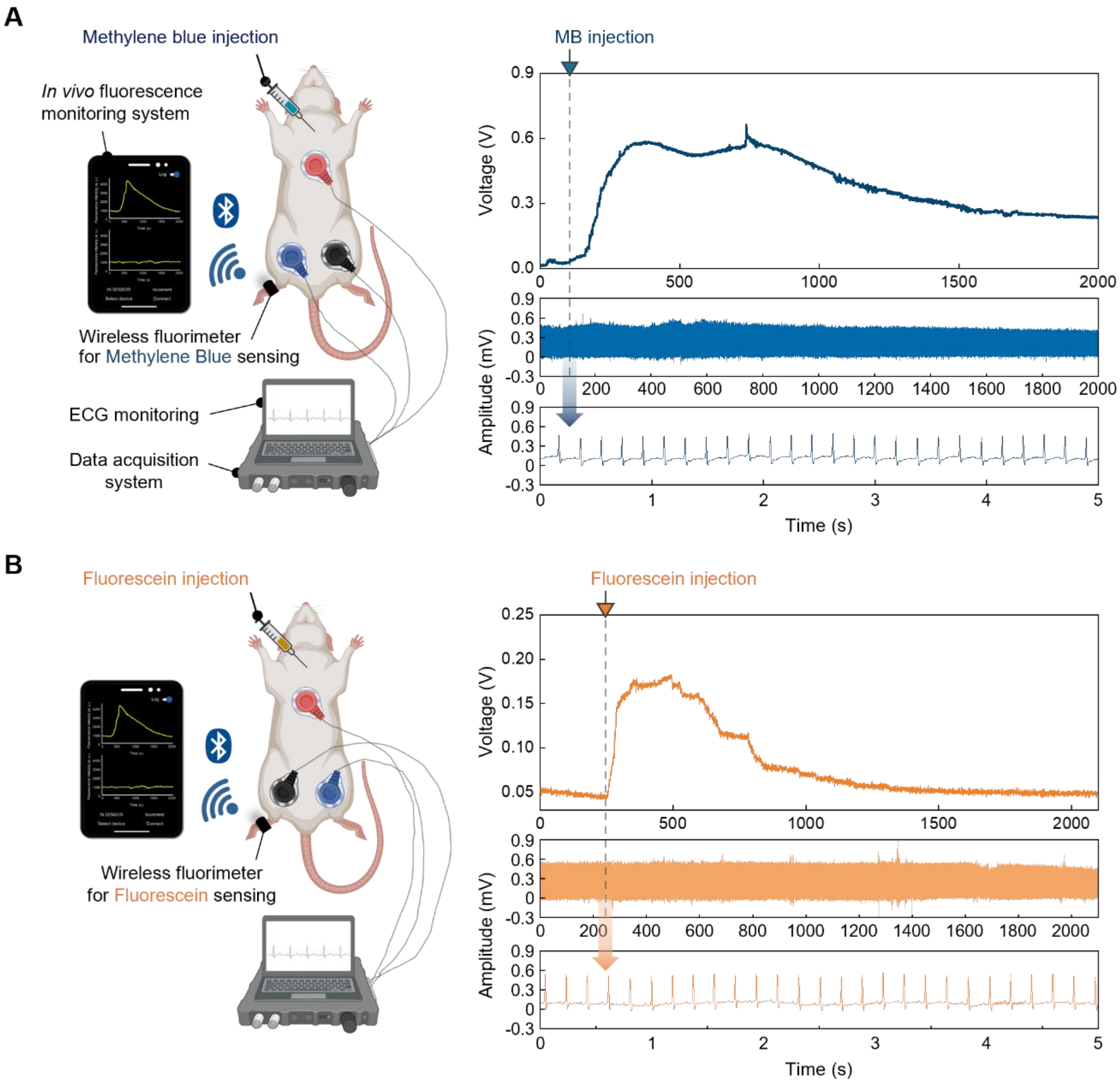

Fig. S21. Real-time fluorescence profiles of various fluorophores. Time-dependent fluorescence profiles of **(A)** MB and **(B)** fluorescein measured by wireless fluorimeter systems customized for each fluorophore. During the entire protocol, electrocardiogram (ECG) signals from the rodent models were recorded, and normal cardiac rhythms were maintained regardless of MB and fluorescein injections. Created in BioRender. Rogers, J. (2026) https://biorender.com/vk3u5ep.

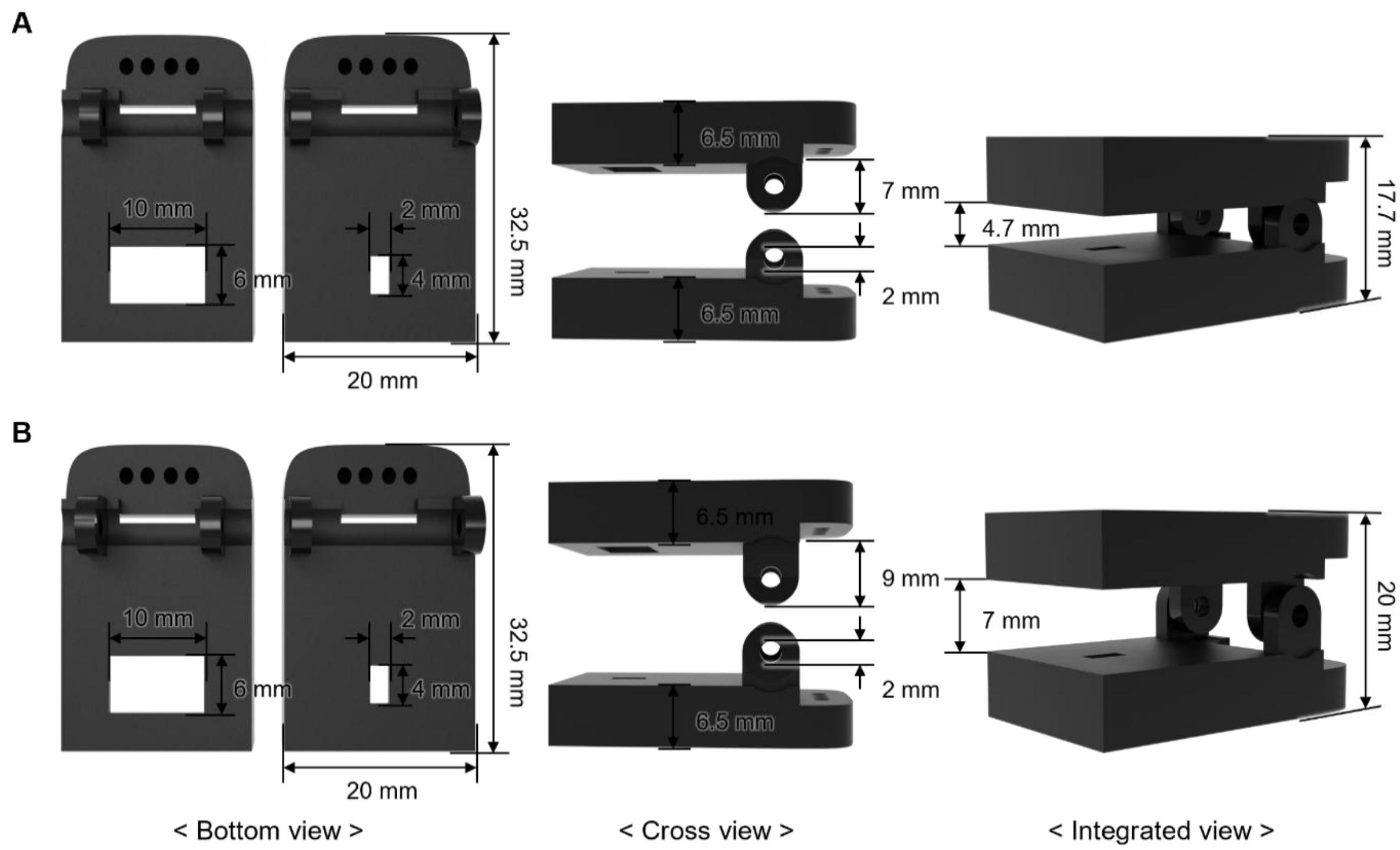

Fig. S22. Tailored clip designs for small animal studies. Dimensions of clips designed for placements on **(A)** rat paw and **(B)** rat tail with thicknesses of 4.7 mm and 7 mm, respectively.

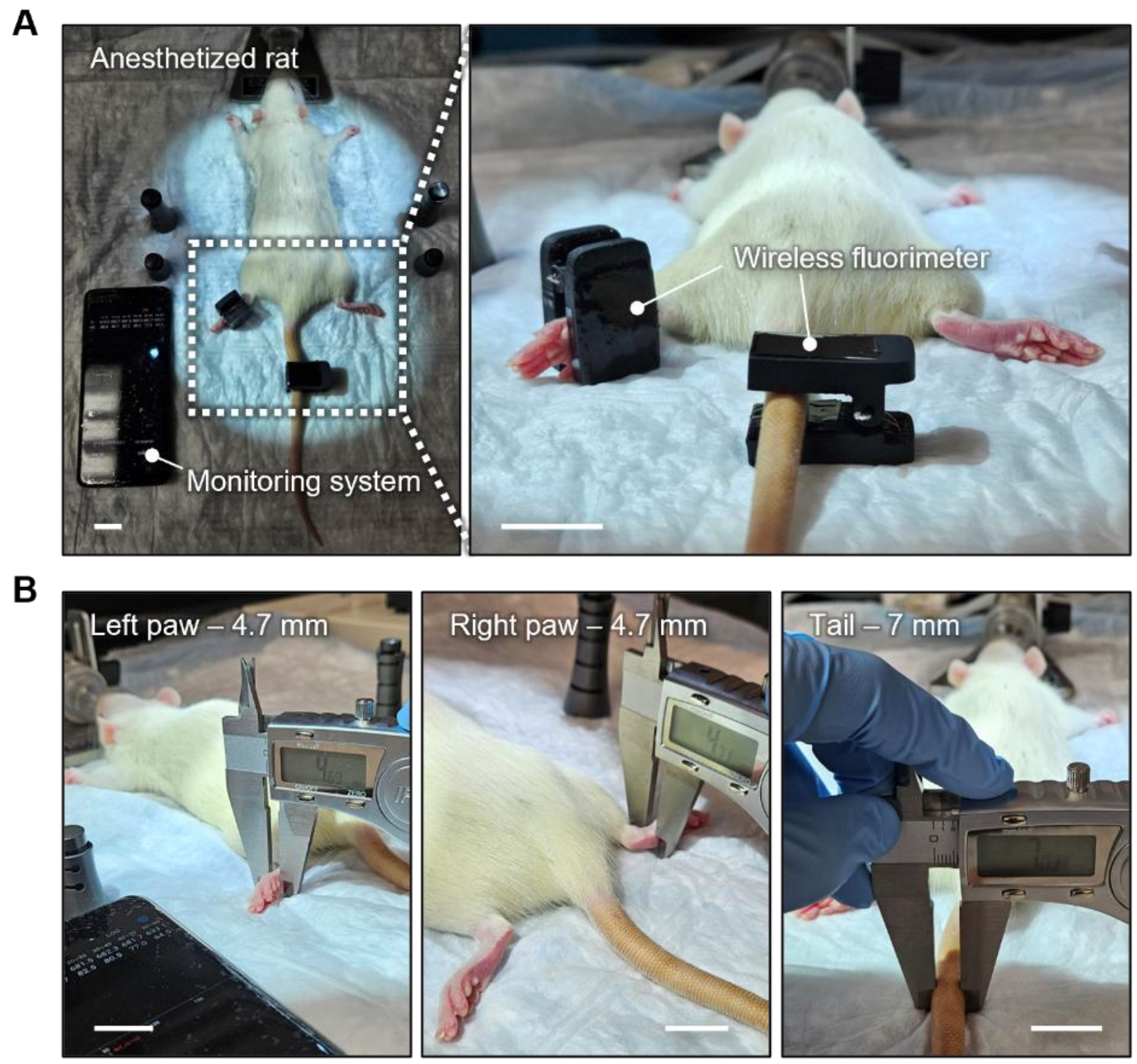

Fig. S23. Tailored devices for placement on the paw and tail of a rat model. (A) Photographs of a rat with a device mounted. **(B)** Measurements of target thicknesses on the paw and tail. Scale bars, 2 cm.

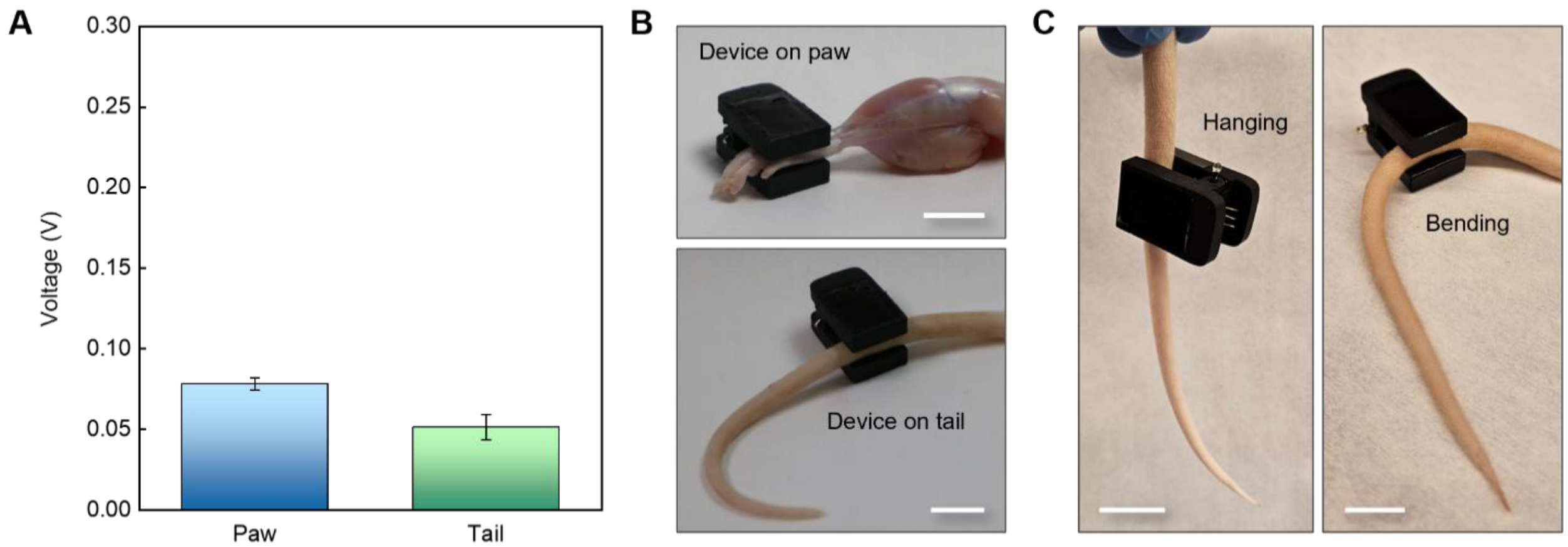

Fig. S24. Device calibration for small animal studies. (A) Initial values of devices depending on the thickness of the paw (4.7 mm) and tail (7 mm). **(B)** Photographs of a device on a rat paw and rat tail. **(C)** Photographs showing reliable attachments under conditions in which the device is suspended from the tail or mounted on a bent tail. Scale bar, 2 cm.

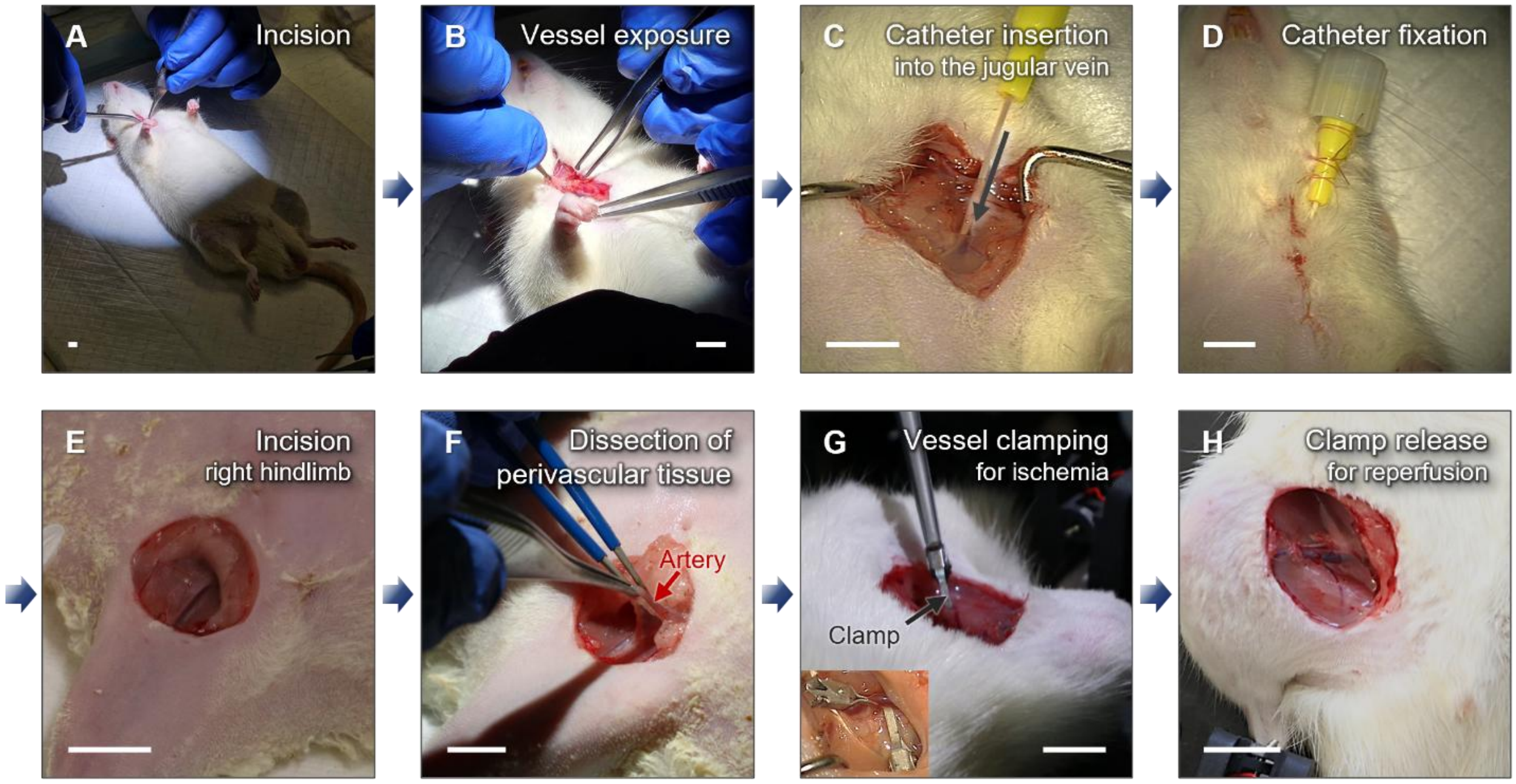

Fig. S25. Surgical procedure in a rat model for hindlimb ischemia. (A, B) Incision for vessel exposure. **(C)** Catheter insertion into the jugular vein for injection of fluorescent material. **(D)** Catheter fixation. **(E, F)** Incision and dissection of perivascular tissue to identify artery and vein prior to ischemia on the hindlimb. **(G)** Vessel clamping for hindlimb ischemia. **(H)** Clamp release for reperfusion. Scale bars, 1 cm.

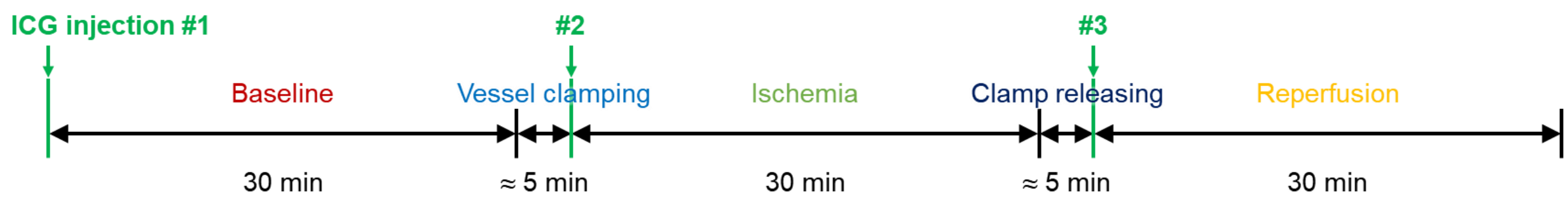

Fig. S26. Experimental protocol for ischemia studies with rodent models.

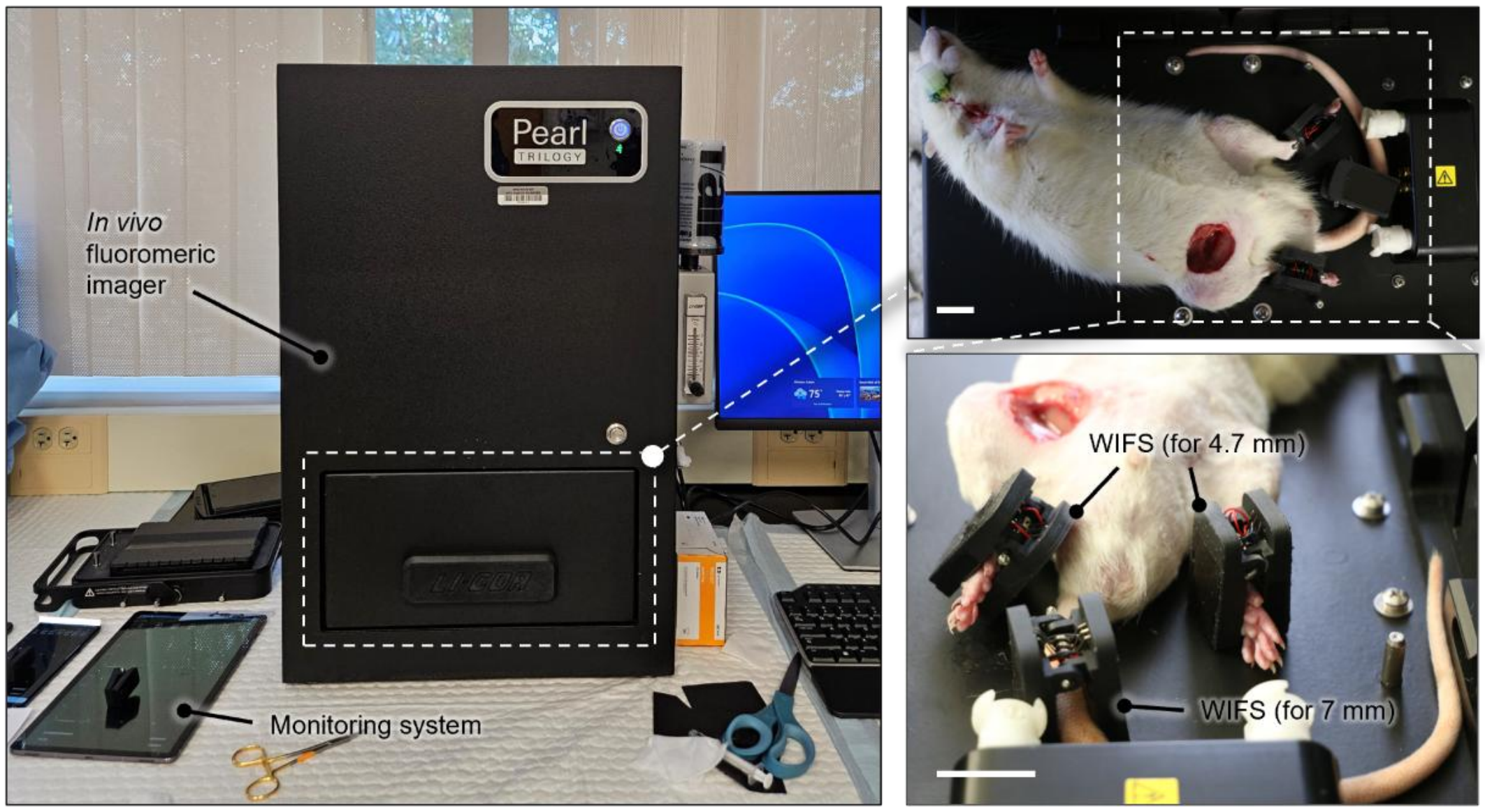

Fig. S27. ***In vivo*** **fluorometric imager.** ICG-injected rats are placed in the fluorometric imager to capture time-dependent fluorescence images during the experimental protocol. Scale bars, 2 cm.

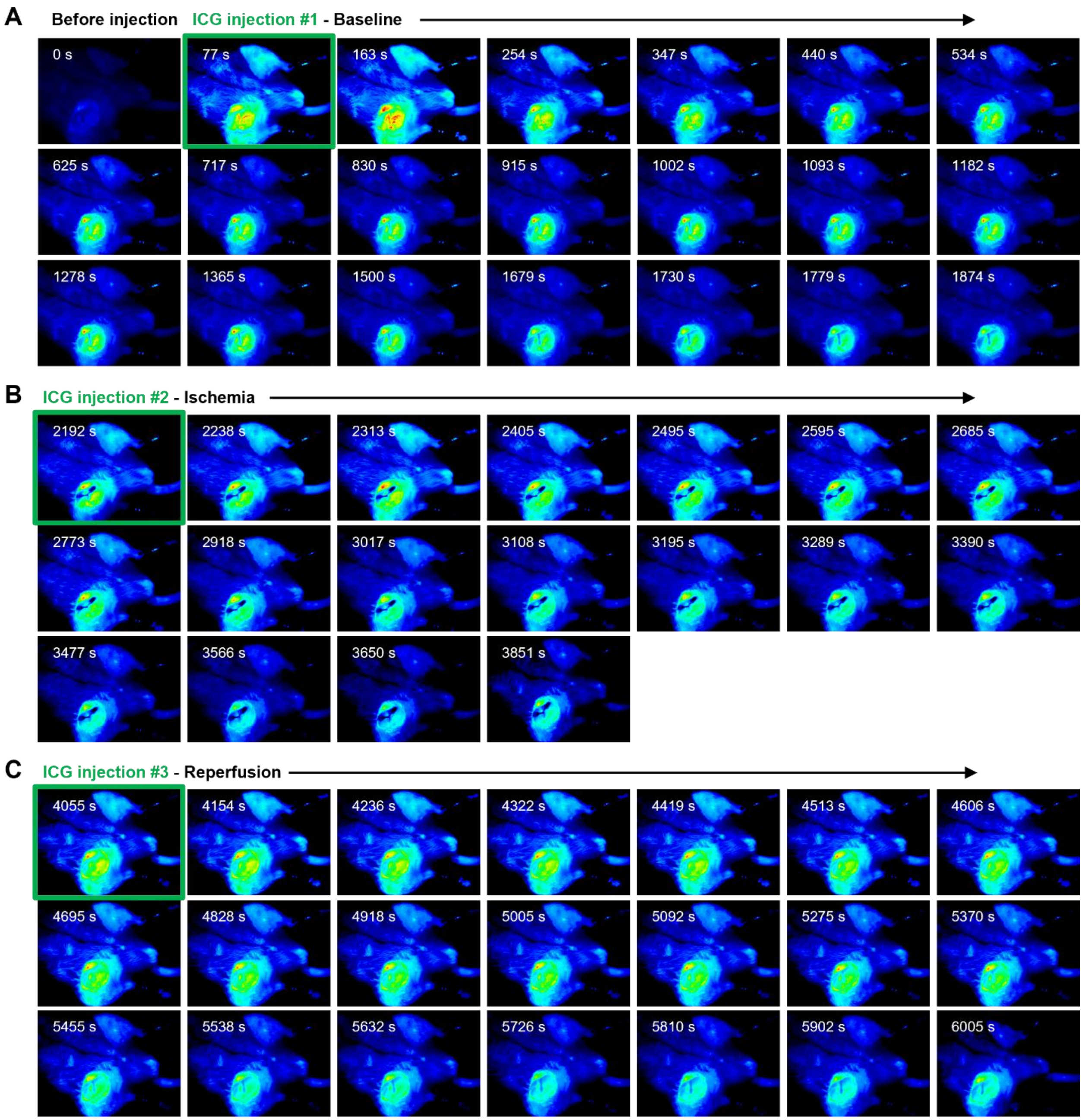

Fig. S28. Quantitative *in vivo* fluorescence imaging of an ICG-injected rat during an ischemia study. Continuously captured fluorescence images during **(A)** baseline, **(B)** ischemia, and **(C)** reperfusion phases. The images at 0, 163, 2238, and 4154 s are shown in **Fig. 4D**.

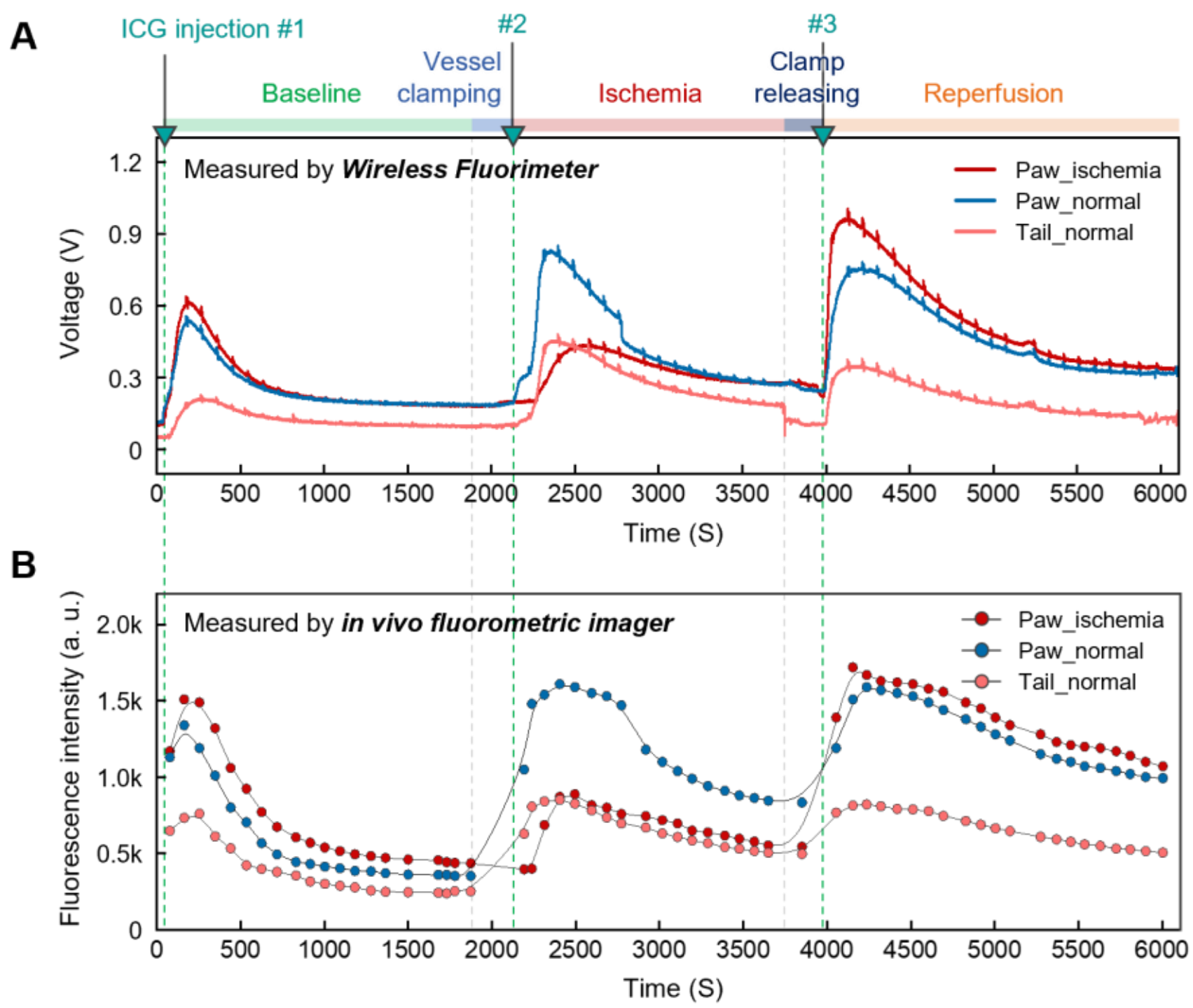

Fig. S29. Comparative analysis of measurements obtained from a wireless fluorimeter and an *in vivo* fluorometric imager. (A) Real-time fluorescence profiles from a wireless fluorimeter. **(B)** Time-dependent fluorescence intensity measured by an *in vivo* fluorometric imager.

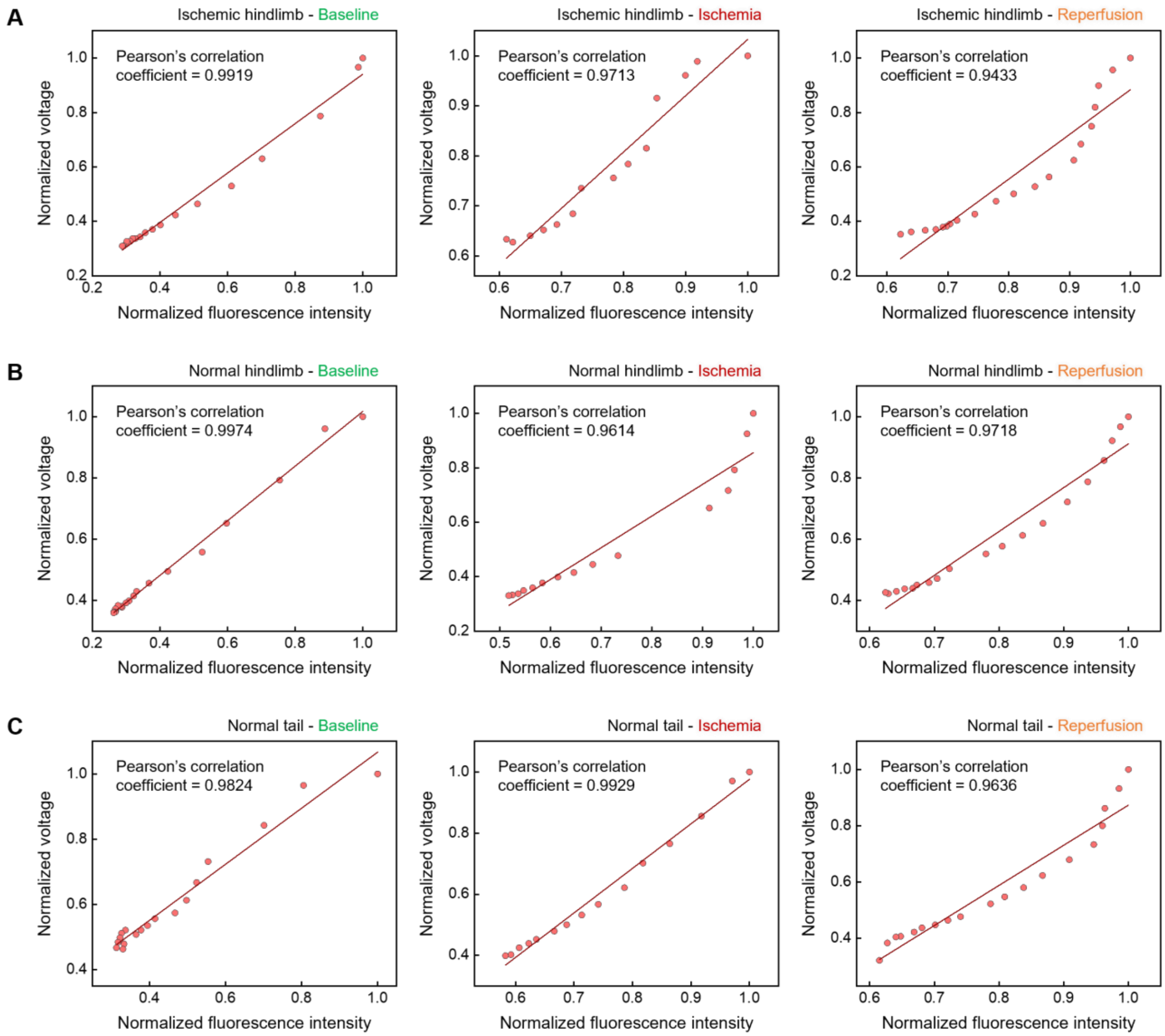

Fig. S30. Correlation between fluorescence intensity measured by a standard fluorescence imager and electrical values measured by a wireless fluorimeter. Data reliability at the **(A)** right ischemia hindlimb, **(B)** left normal hindlimb, and **(C)** normal tail during the three steps of baseline, ischemia, and reperfusion. All measurements exhibit strong reliability, with Pearson's correlation coefficient exceeding 0.94.

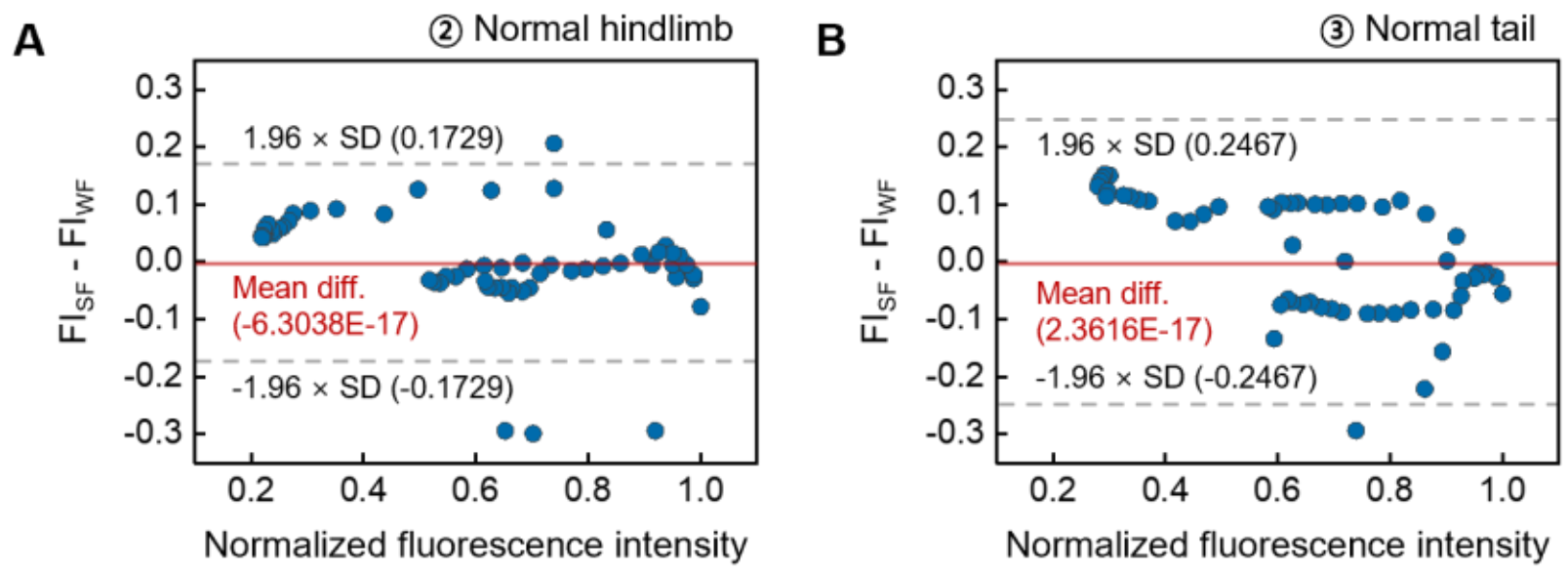

Fig. S31. Bland-Altman plots comparing fluorescence intensity determined by a wireless fluorimeter (WF) and a standard fluorometer (SF) in the (A) normal hindlimb and (B) normal tail.

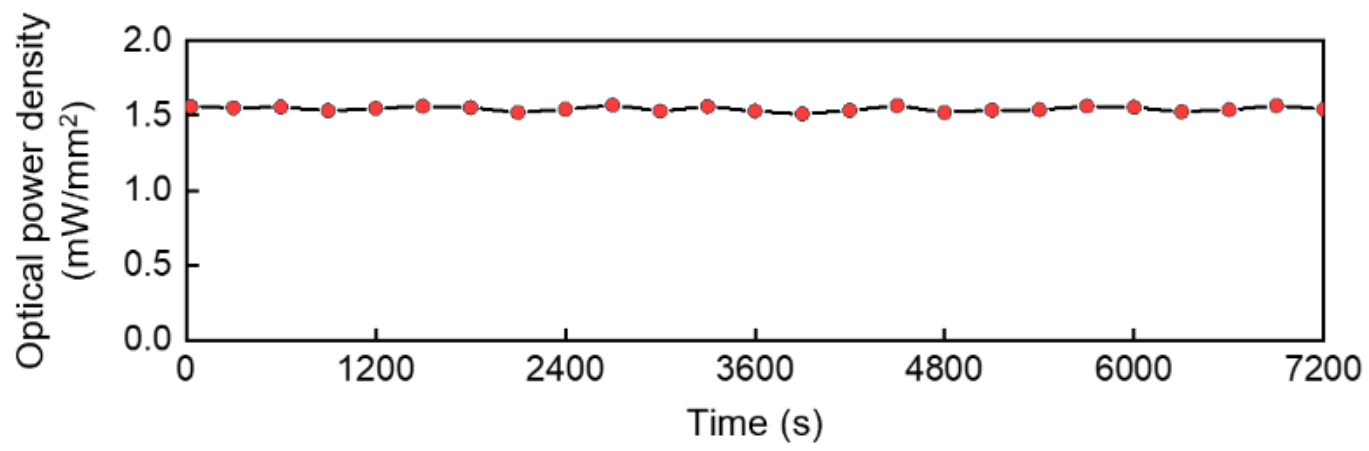

Fig. S32. Long-term stability of optical power density. The NIR LED exhibits a mean optical power density of 1.55 mW/mm^2 with a low variation of 1.65%, showing stable operation over 2 hours.

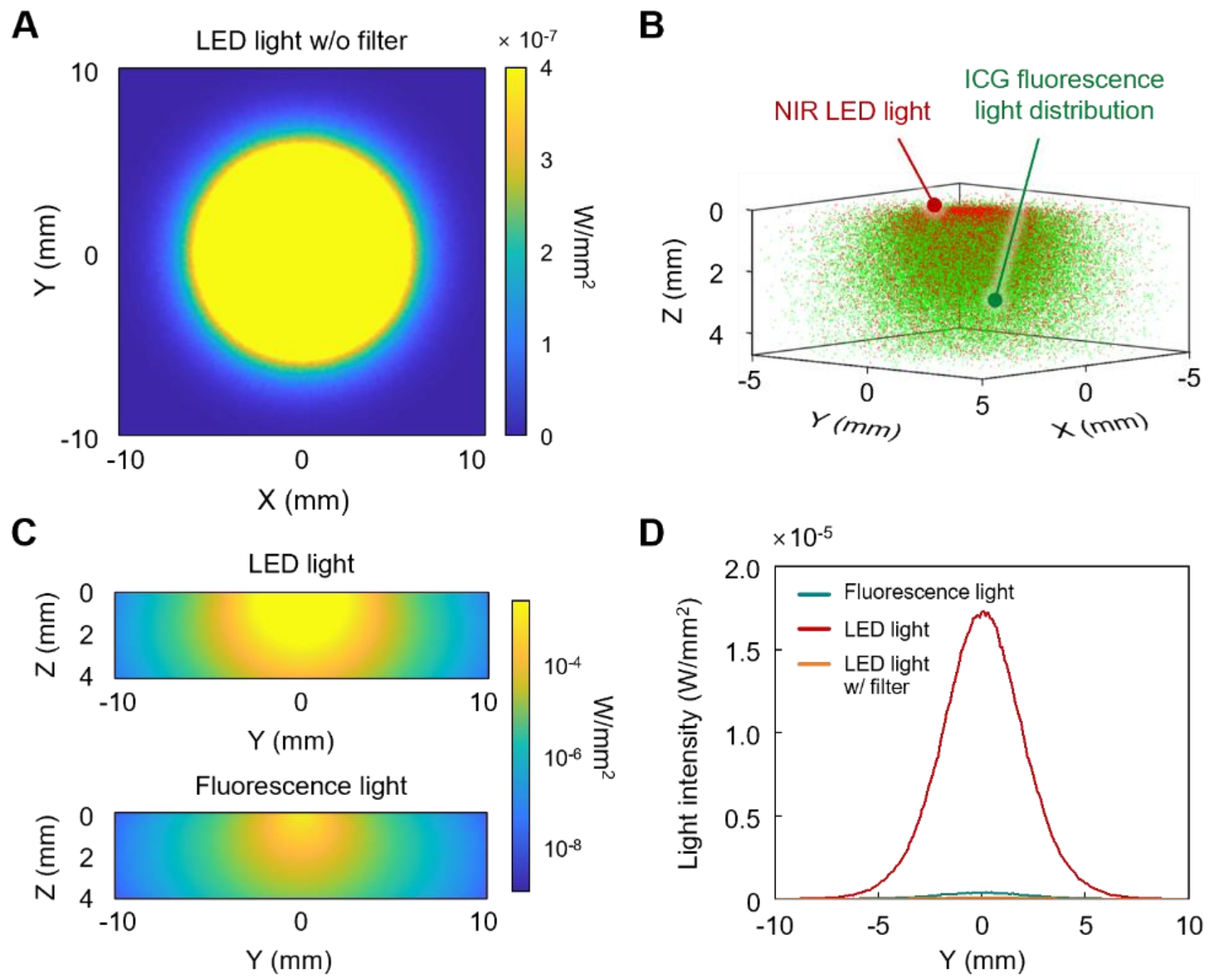

Fig. S33. Monte Carlo simulation for the rat paw. (A) Light intensity distribution at the bottom surface. **(B)** Three-dimensional light intensity distribution. **(C)** Light intensity distribution in a cross-sectional plane. **(D)** Light intensity distribution at the bottom surface along $X = 0$.

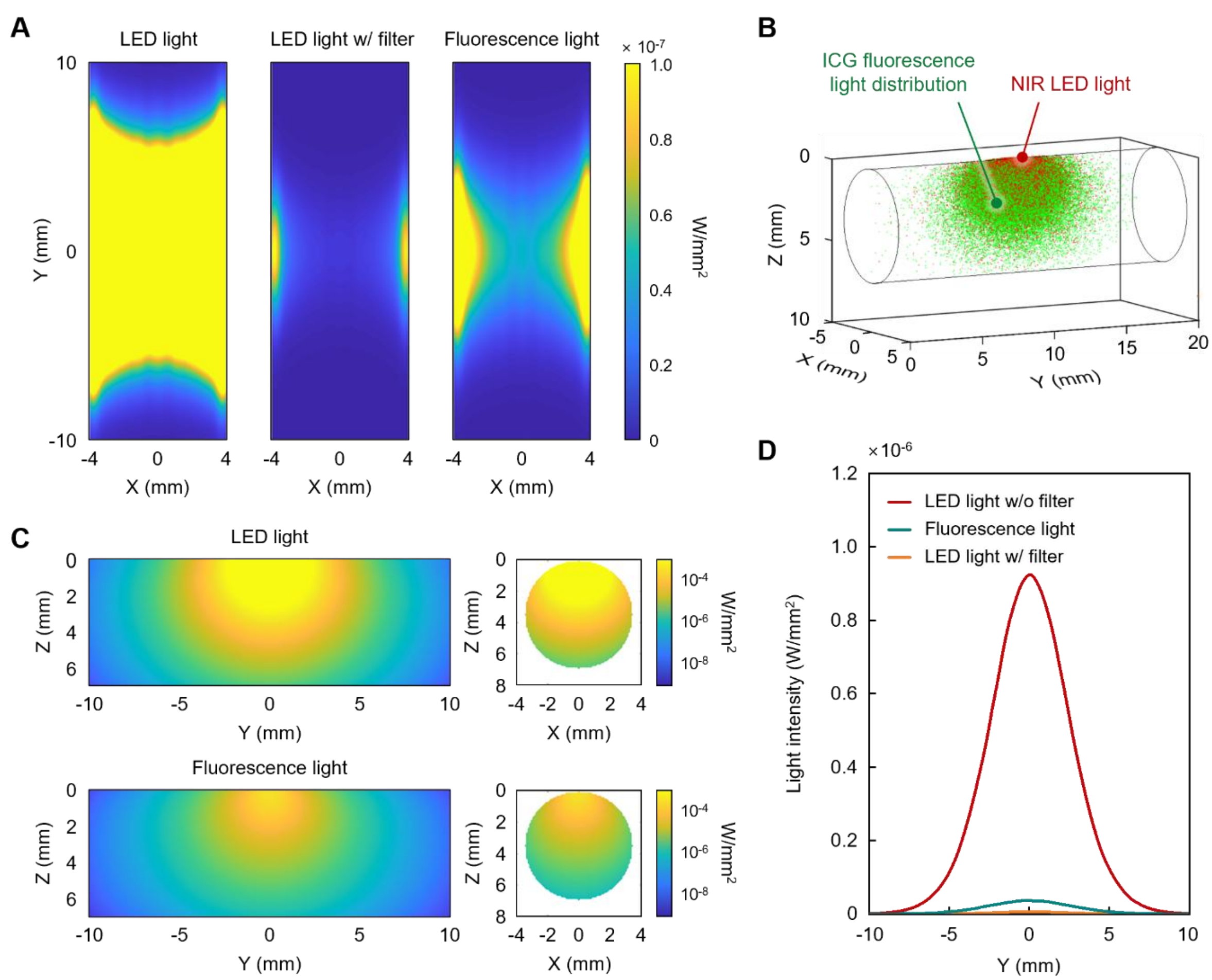

Fig. S34. Monte Carlo simulation for the rat tail. (A) Light intensity distribution at the bottom half of the cylindrical surface. **(B)** Three-dimensional light intensity distribution. **(C)** LED light and fluorescence intensity distributions in cross-sectional planes perpendicular to the X- and Y-directions. **(D)** 1D intensity profiles of LED light, LED light with filter, and fluorescence light along $X = 0$.

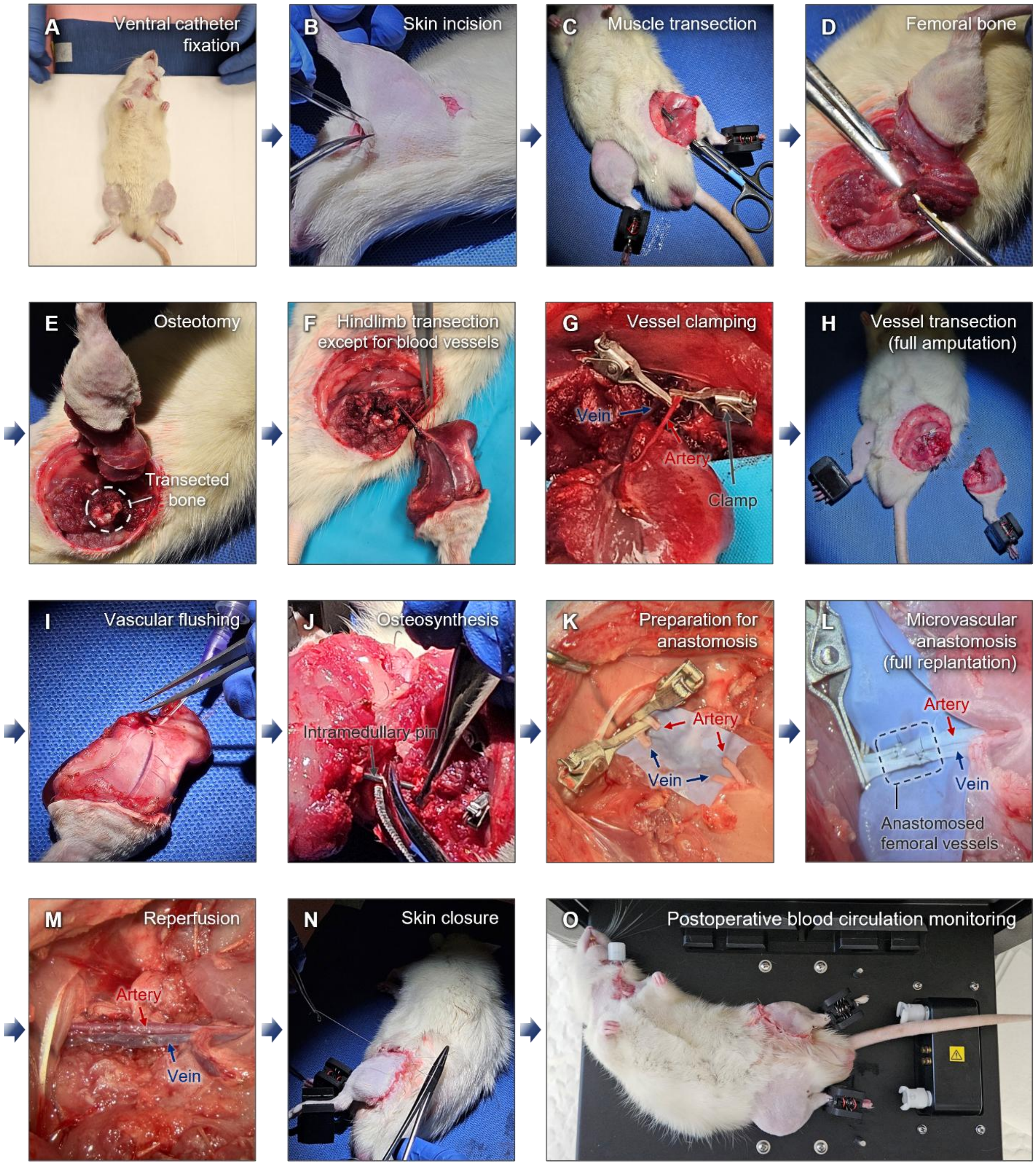

Fig. S35. Surgical workflow of the rat hindlimb amputation-replantation model. **(A)** Shaving and jugular catheter insertion. **(B)** Skin incision. **(C-E)** Transection of muscle, soft tissue, and femoral bone. **(F)** Hindlimb transection except for artery and vein. **(G)** Vessel clamping. **(H)** Transection of artery and vein. **(I)** Vascular flushing. **(J)** Bone fixation. **(K)** Transected artery and vein. **(L)** Microvascular anastomosis. **(M)** Clamp releasing. **(N)** Skin closure. **(O)** Postoperative perfusion monitoring.

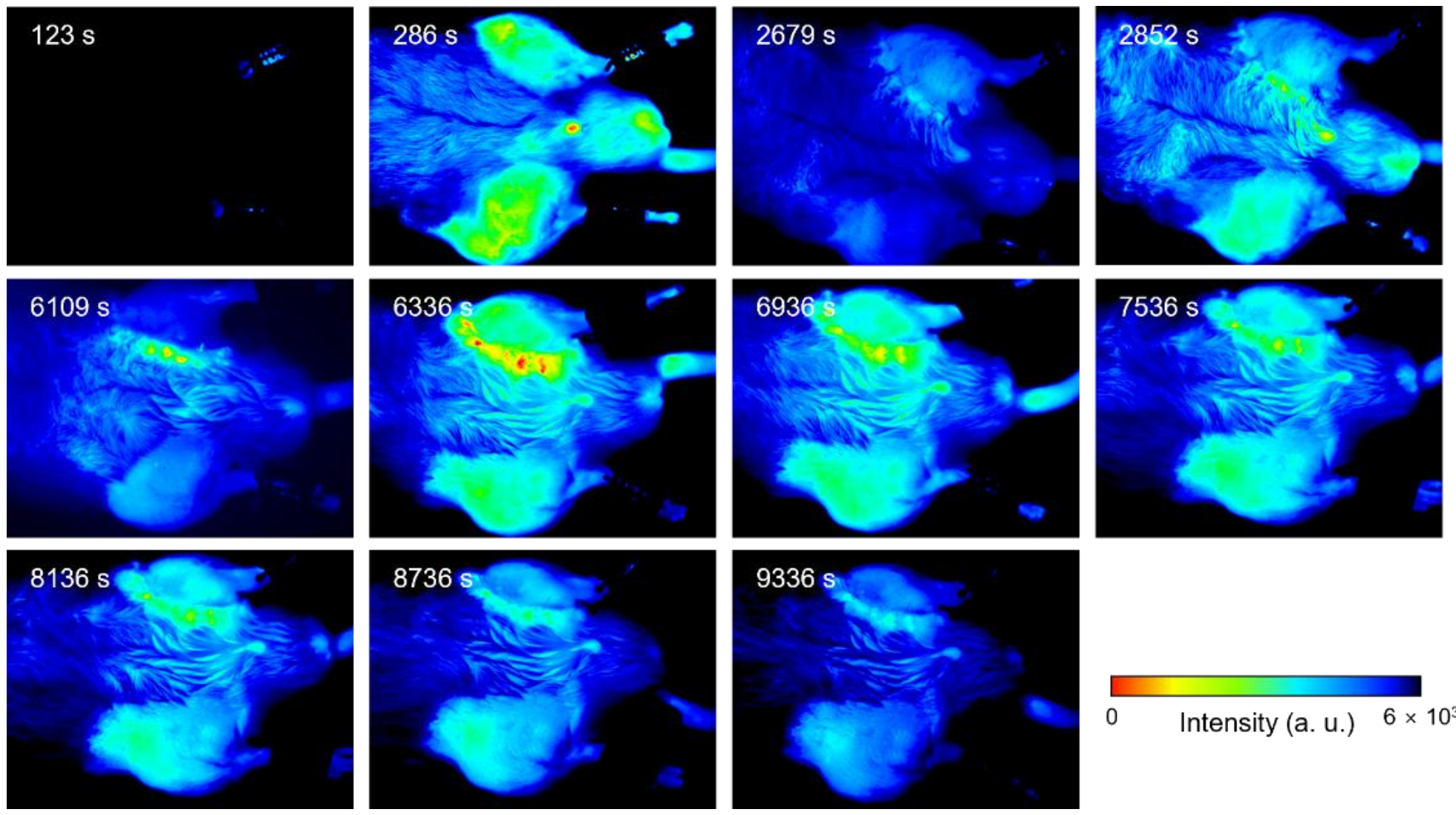

Fig. S36. Quantitative *in vivo* fluorescence imaging of an ICG injected rat during the amputation-replantation protocol. Continuously captured fluorescence images using *in vivo* fluorometric imager. The images at 123, 286, 2852, and 6336 s are shown in **Fig. 5C**.

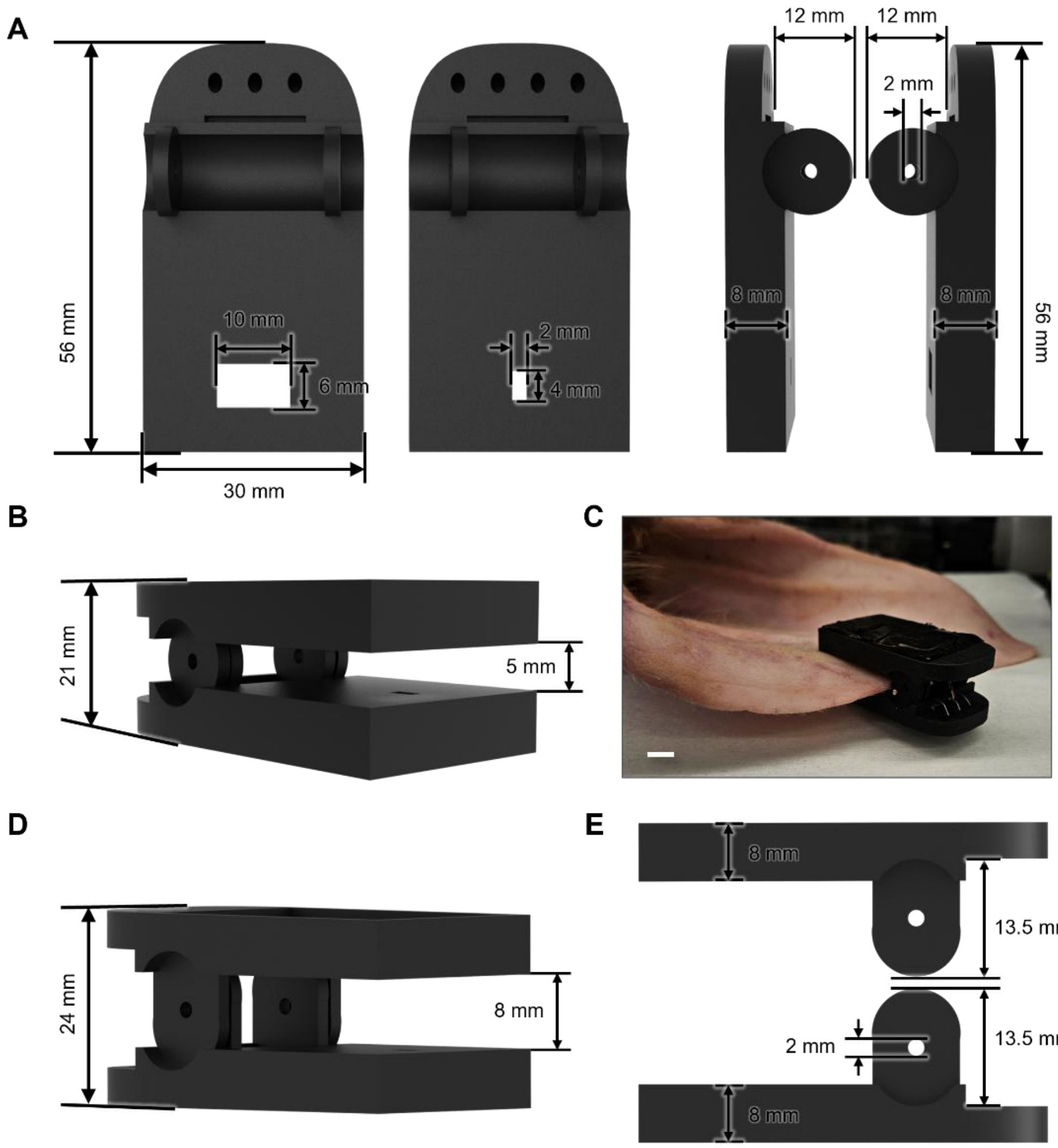

Fig. S37. Dimensions of wireless fluorimeters for large animal studies. (A, B) Device designed for placement onto porcine ear with a thickness of 5 mm. **(C)** Photograph of wireless fluorimeter mounted on a porcine ear. Scale bar, 1 cm. **(D, E)** Schematic illustrations of the device for placement on the porcine lip with a thickness of 8 mm.

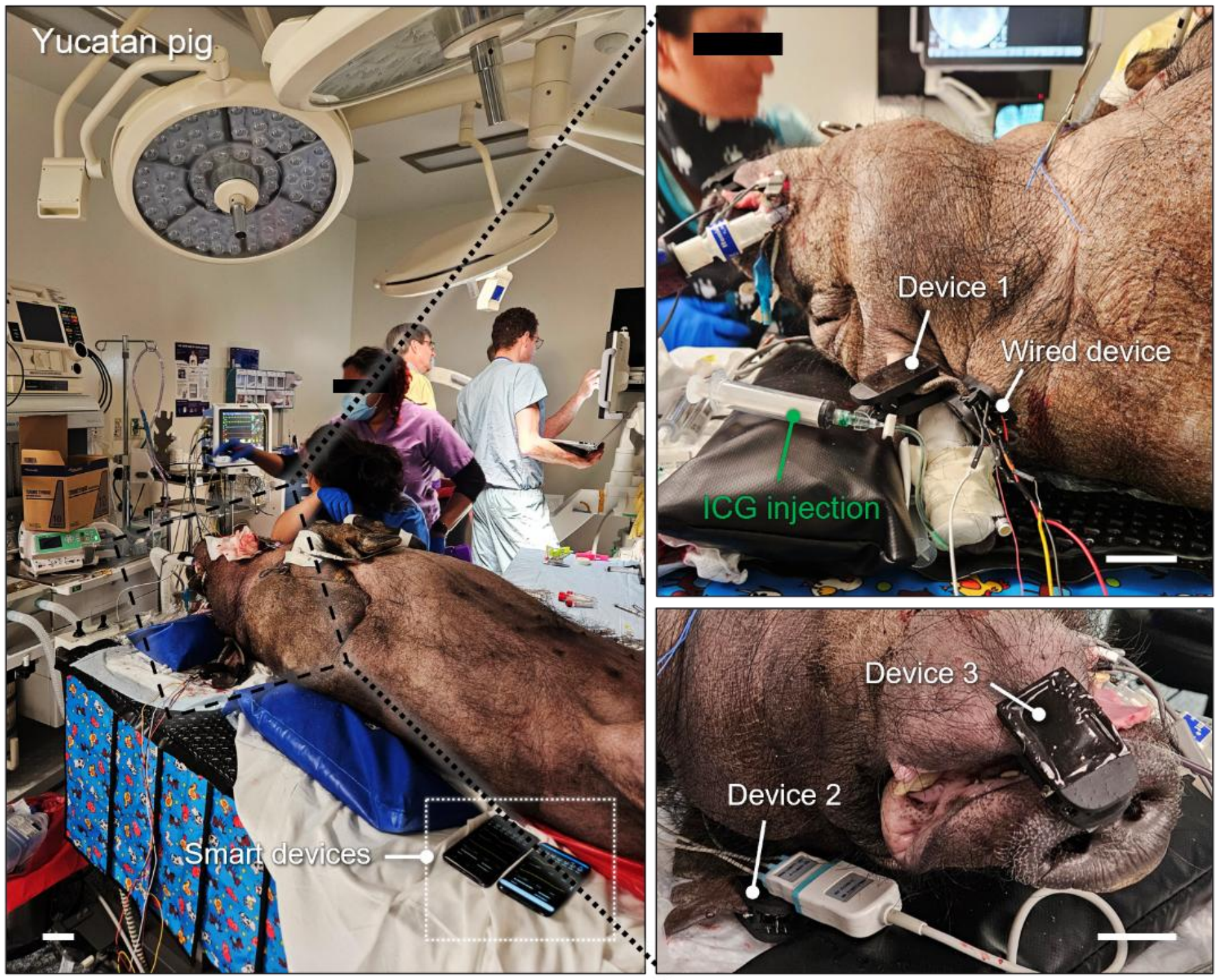

Fig. S38. Fluorescence measurement setup in an anesthetized Yucatan pig model. Scale bars, 5 cm.

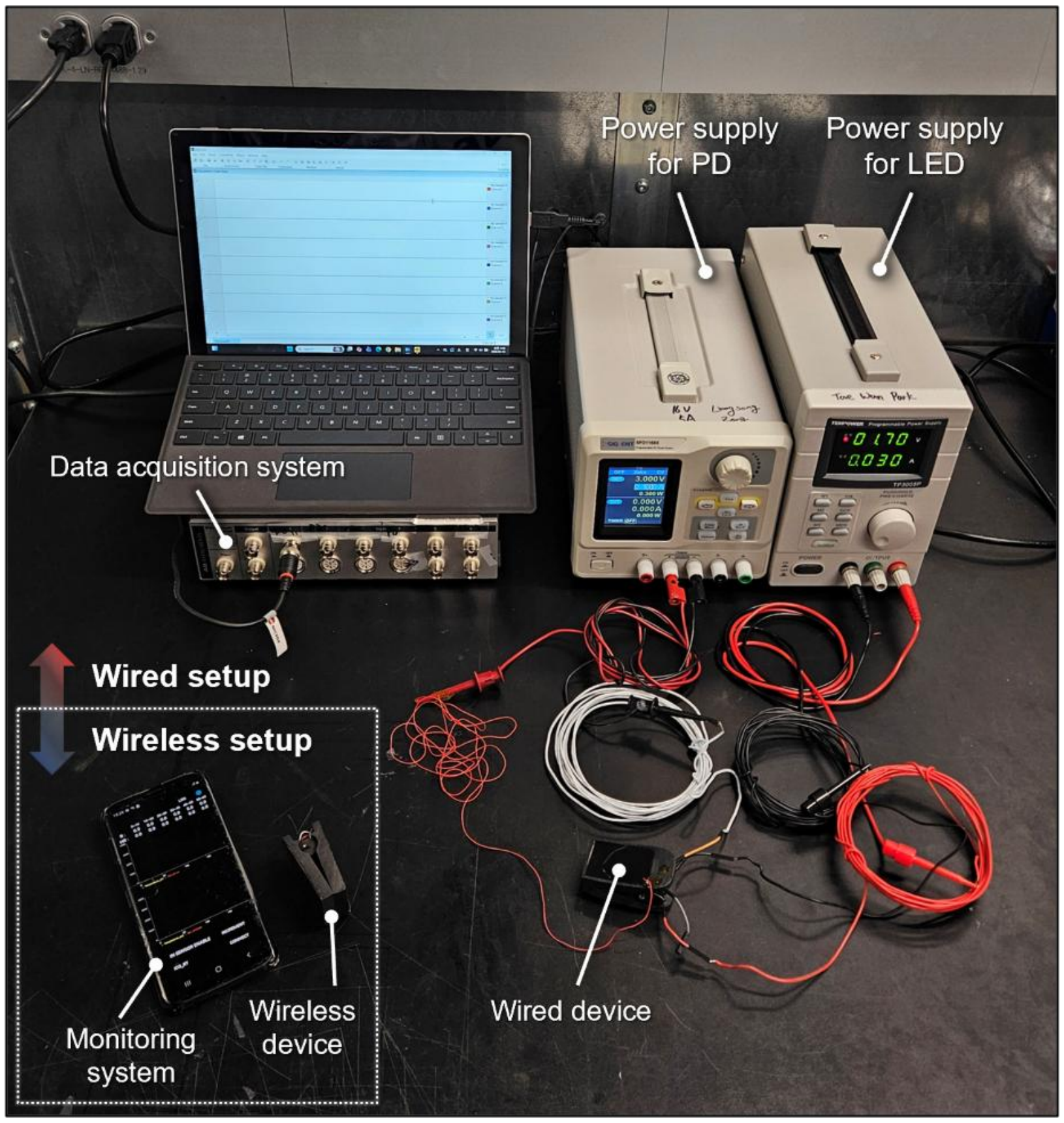

Fig. S39. Experimental setup for wired fluorescence sensing compared to that for a wireless setup.

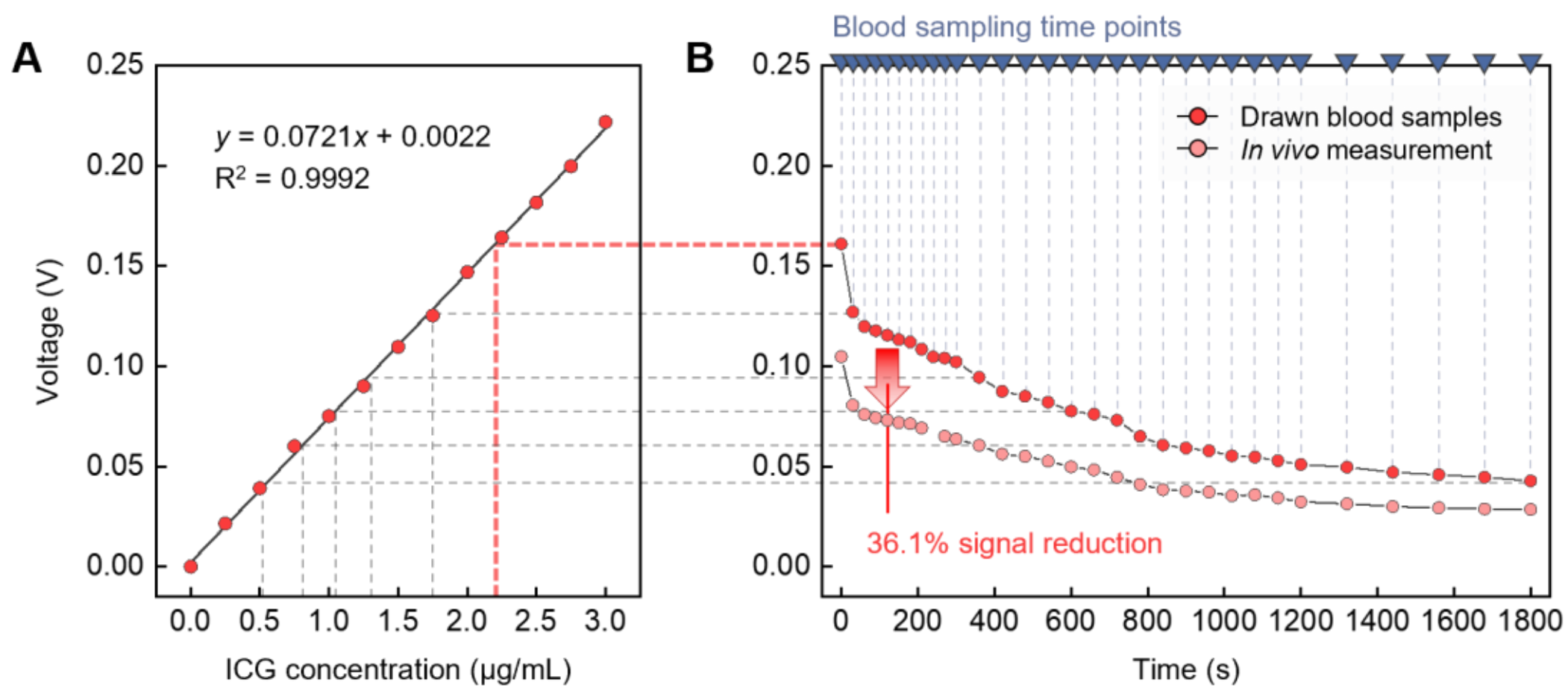

Fig. S40. Concentration-dependent quantification of voltage responses from the wireless fluorimeter in porcine model. (A) Benchtop quantification of voltage responses for ICG in porcine blood with varying concentrations. **(B)** Voltage outputs from the drawn blood samples (red dots) and *in vivo* measurement (pink dots) in porcine study. The peak voltage corresponds to 2.20 μg/mL, as determined from benchtop calibration (red dashed line), showing strong agreement with the simulated result (2.17 μg/mL).

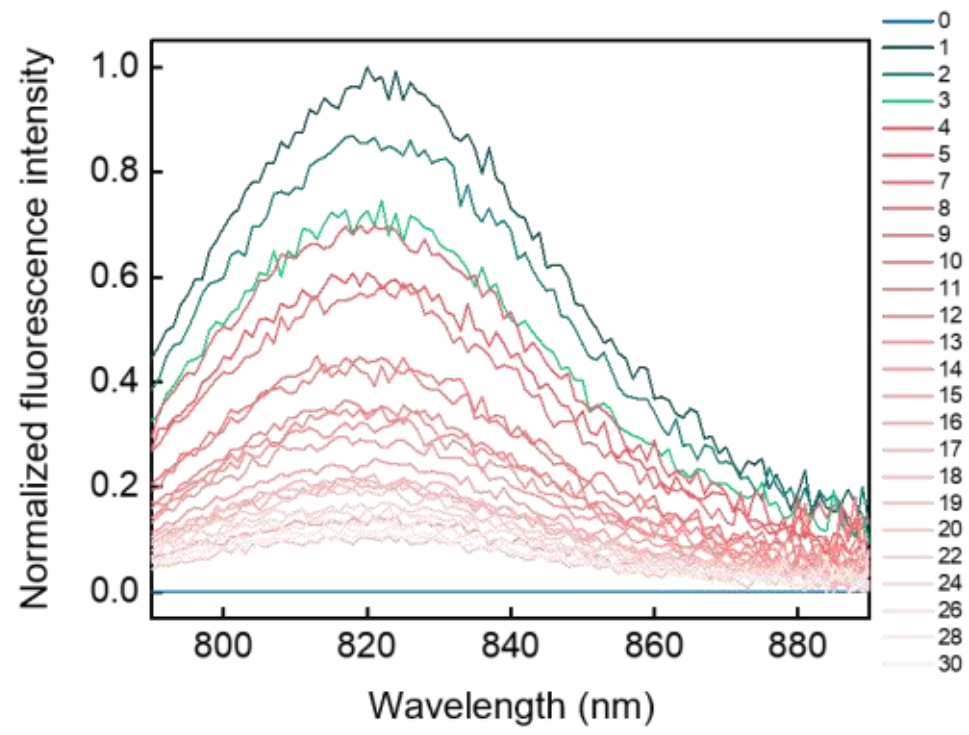

Fig. S41. 1D emission spectra showing fluorescence efficiency of drawn blood samples.

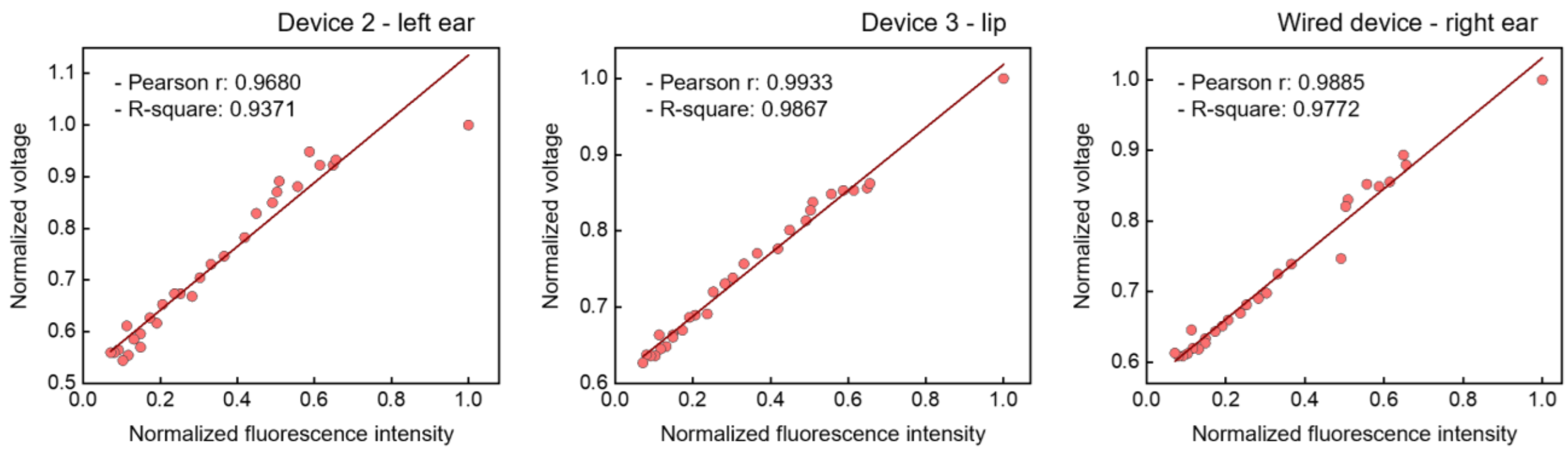

Fig. S42. Correlation between the electrical outputs from the wireless fluorimeter and the fluorescence intensity measured by a standard fluorometer in an ICG-injected Pig 1.

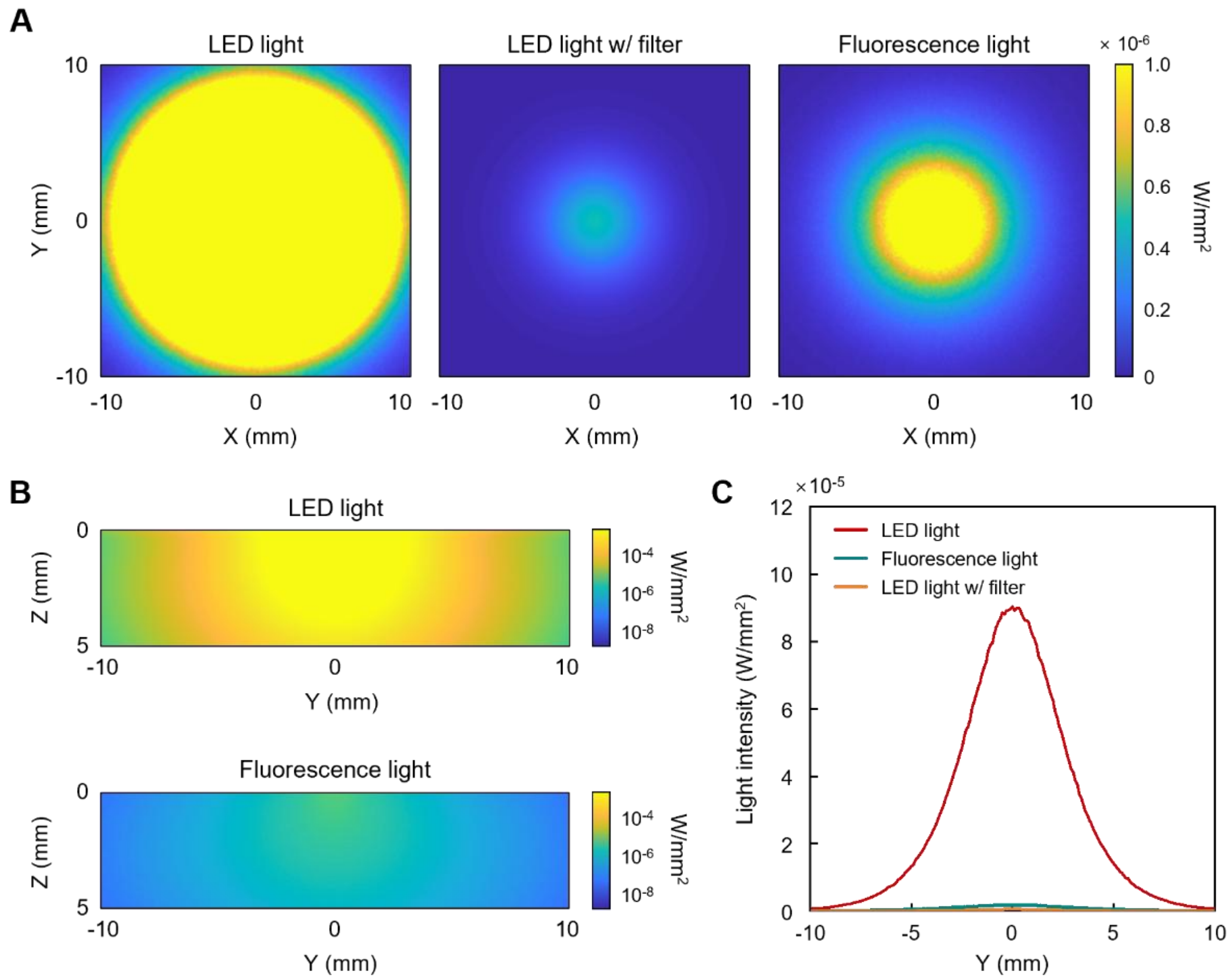

Fig. S43. Monte Carlo simulation for the porcine ear. (A) Light intensity distribution at the bottom surface. **(B)** Light intensity distribution in a cross-sectional plane. **(C)** 1D light intensity profiles of LED light, LED light with filter, and fluorescence light in the pig ear at the bottom surface along $X = 0$.

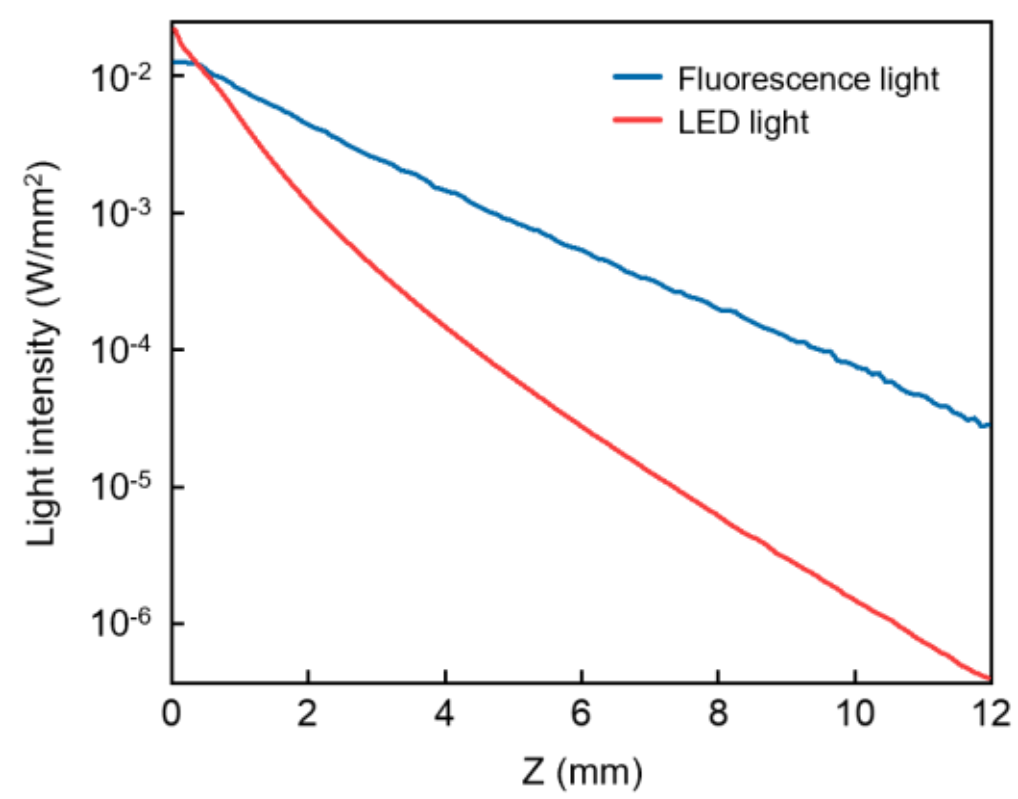

Fig. S44. LED (red line) and fluorescence (blue line) intensities as functions of tissue depth when the filter is applied.

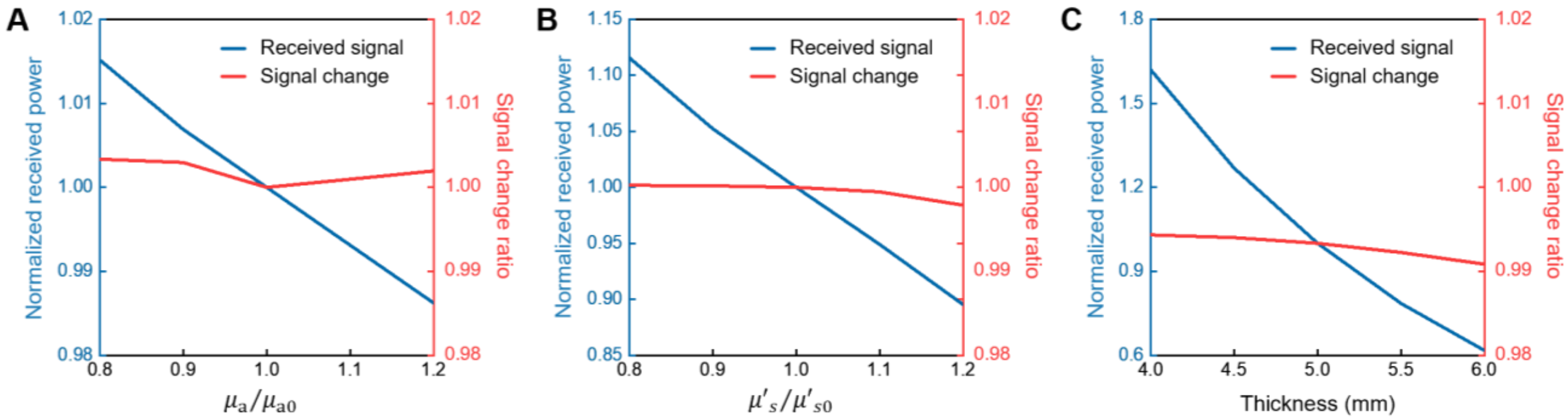

Fig. S45. Normalized received power (blue lines) after ICG injection as functions of (A) tissue absorption coefficient, (B) tissue reduced scattering coefficient, and (C) tissue thickness. Red lines denote the ratio of the received power after and before ICG injection.

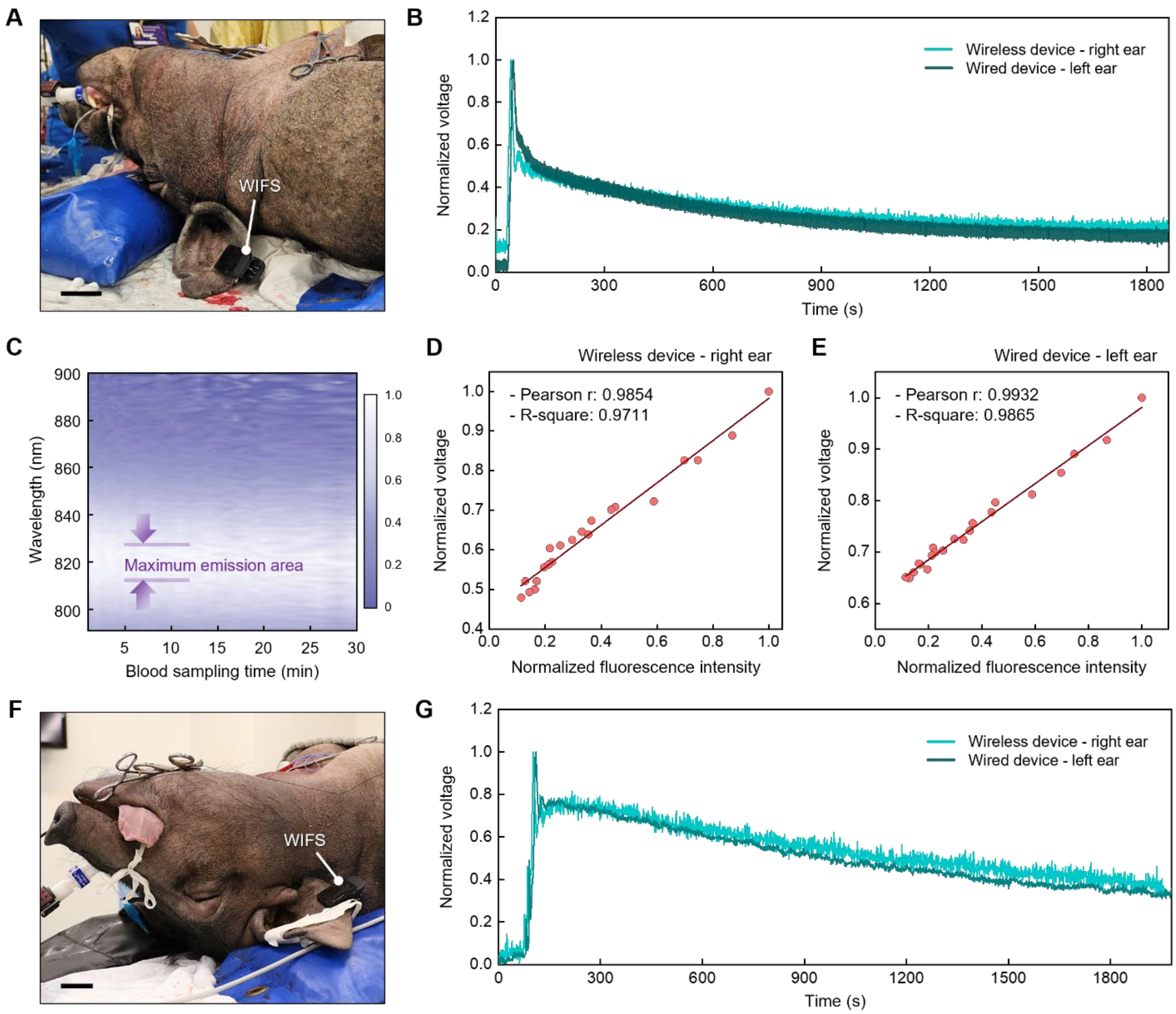

Fig. S46. Additional large animal study. (A) Photograph of a device mounted in a Yucatan pig (Pig 2). **(B)** Real-time fluorescence dynamics measured by the wired and wireless fluorimeters. **(C)** Fluorescence intensity map showing the peak emission wavelength of ICG from collected blood samples. **(D, E)** Correlation between fluorescence intensity measured by a standard fluorometer and the wired and wireless fluorimeters. **(F)** Photograph of Pig 3 during the demonstration. **(G)** Real-time fluorescence profiles measured by the wired and wireless fluorimeters in Pig 3, showing excellent agreement. Scale bars, 5 cm.

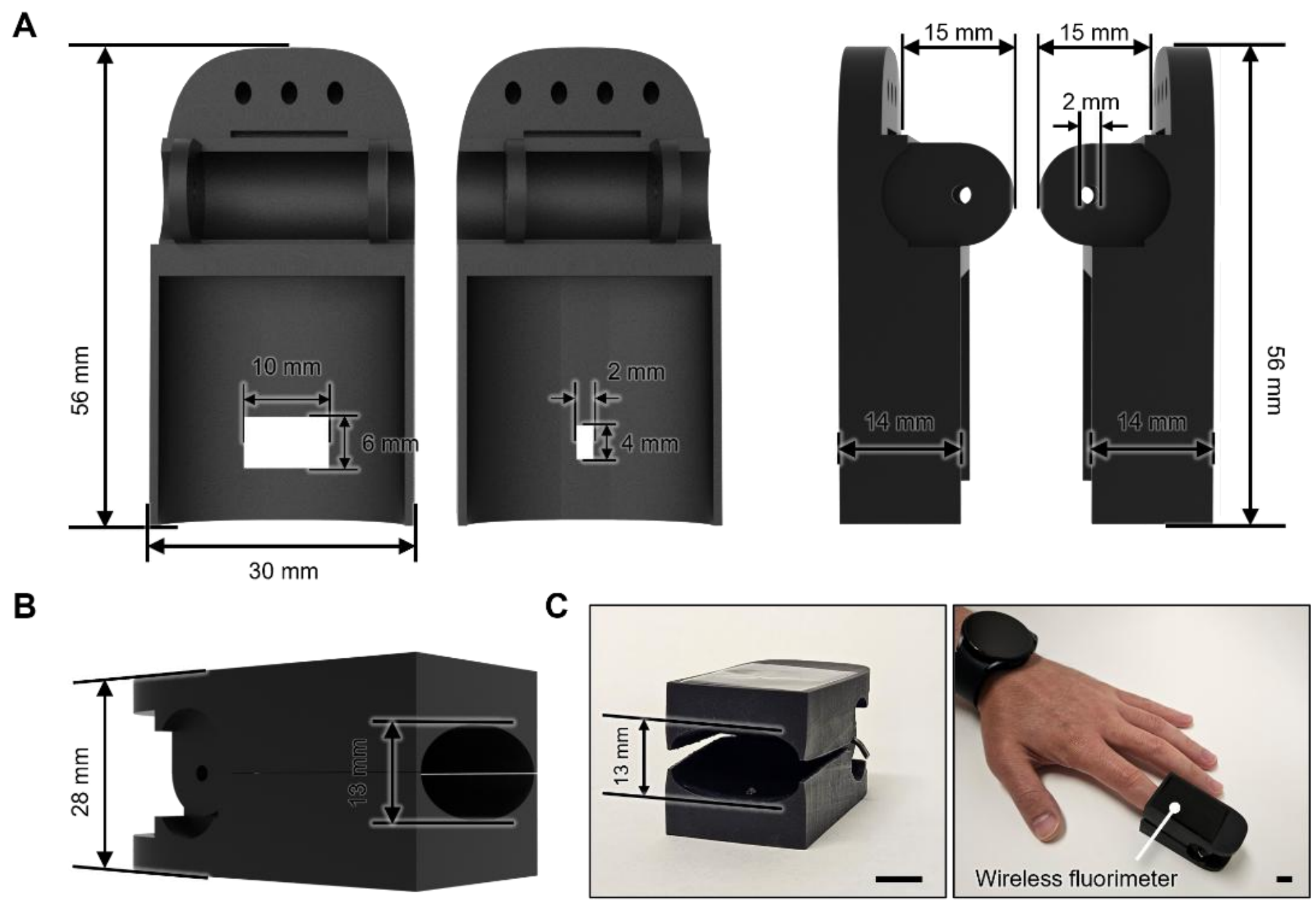

Fig. S47. Engineering of the device platform for human application. (A, B) Dimensions of a finger-clip WIFS designed for measurements on a human finger. **(C)** Photographs of WIFS for human use. Scale bars, 1 cm.

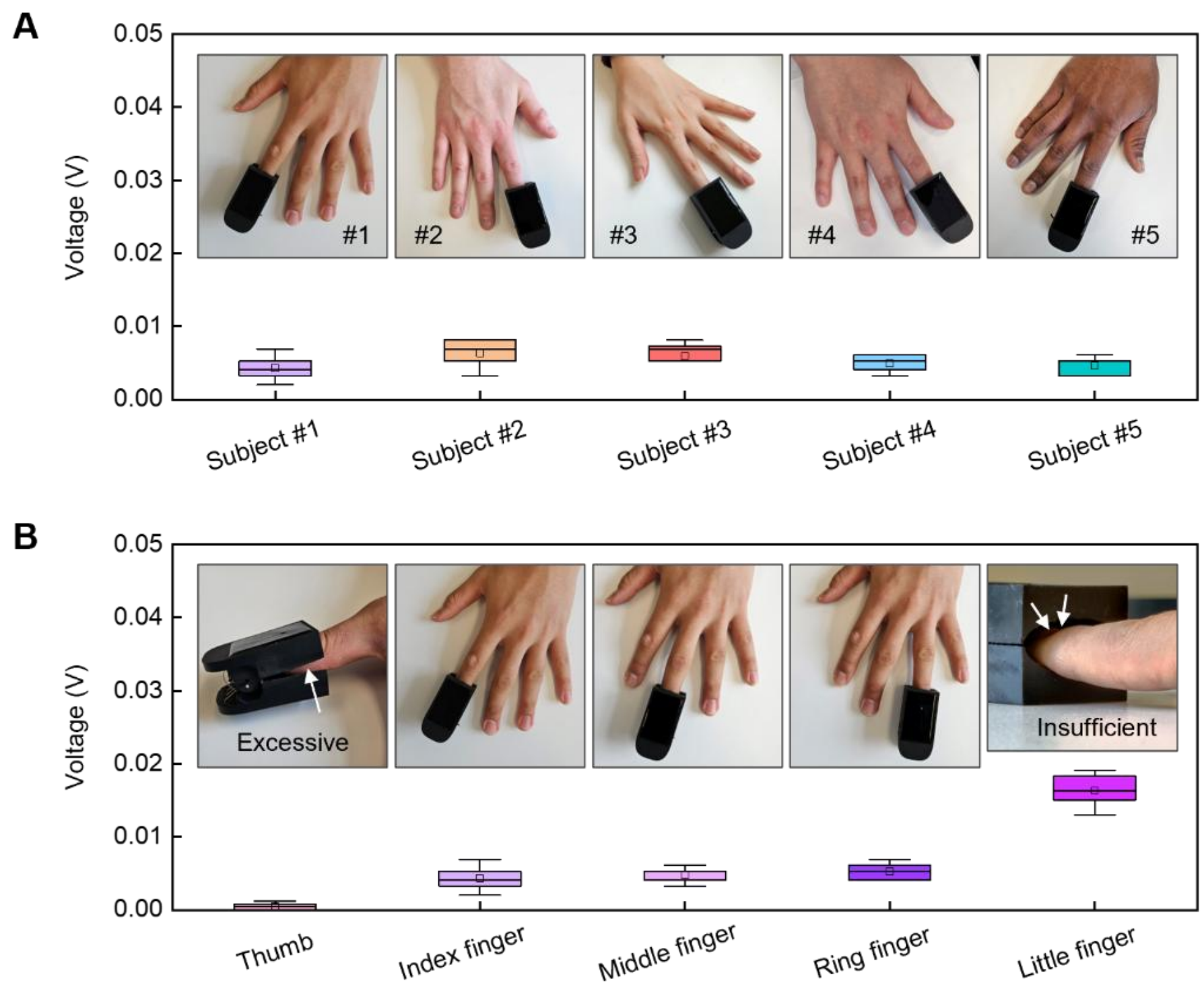

Fig. S48. Thickness-dependent device calibration for use on the human finger. (A) Measurements from five subjects with the index finger fully inserted into the device configured for a thickness of 13 mm. **(B)** Measurements across different fingers of a single subject with varying thicknesses (Thumb, 15 mm; index, middle, and ring fingers, 13 mm; little finger, 11 mm).

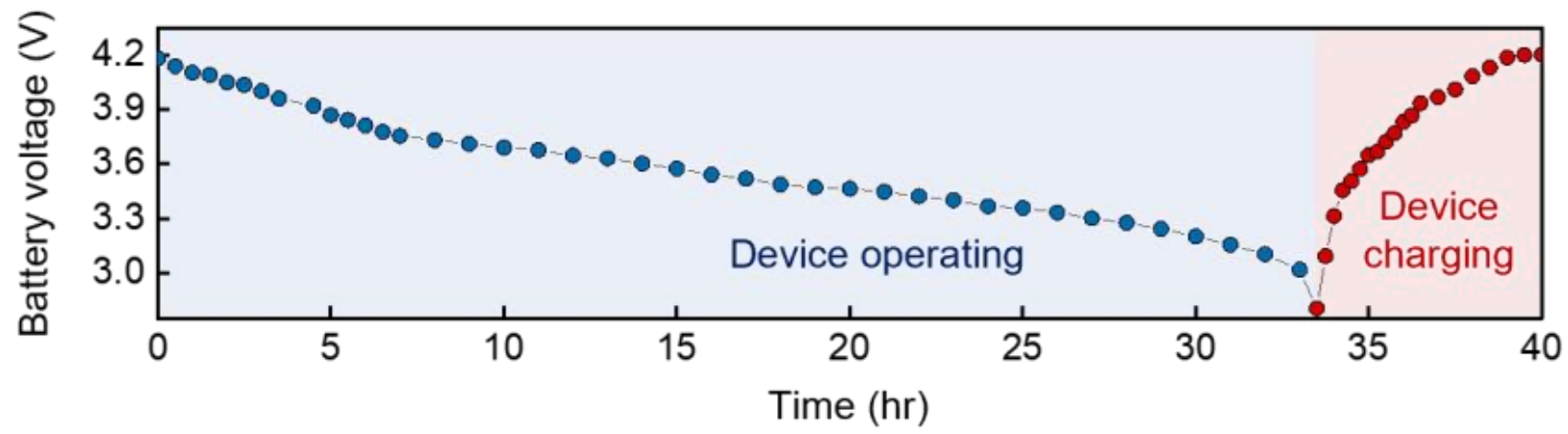

Fig. S49. Device life of wireless fluorimeter system. Battery voltage profile over operating time for a fully charged battery, followed by the charging behavior of a depleted battery.

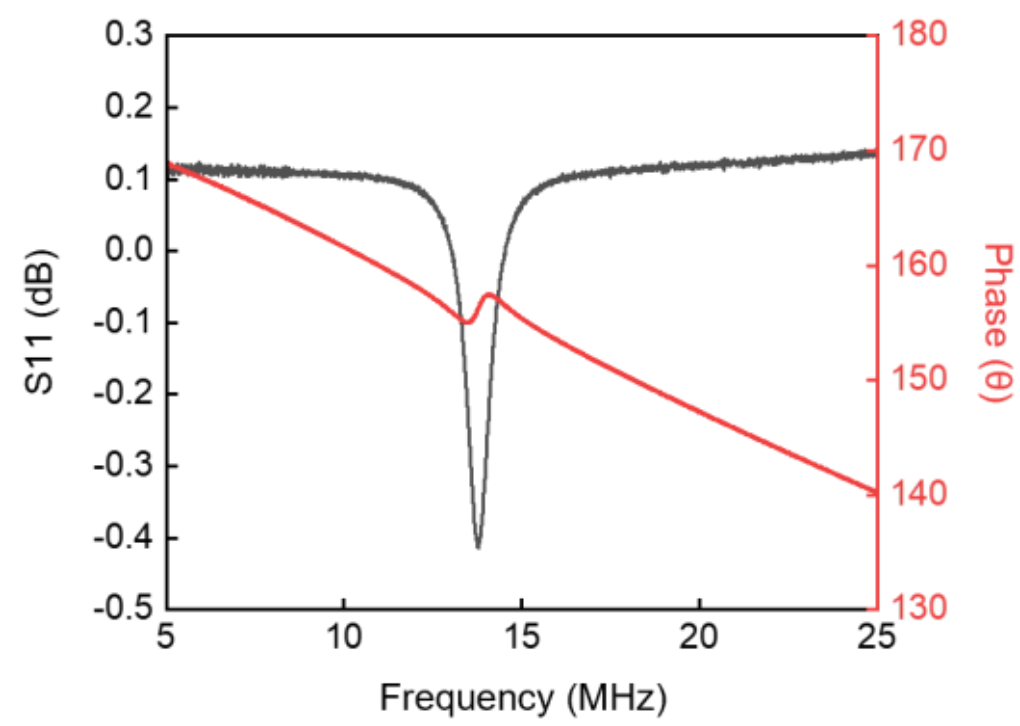

Fig. S50. Resonance frequency of RX coil in the wireless fluorimeter (black, S11; red, phase). The integrated coil with a 13.56 MHz resonance frequency allows magnetic coupling for effective wireless charging with successful near-field communications in both unencapsulated and encapsulated devices.

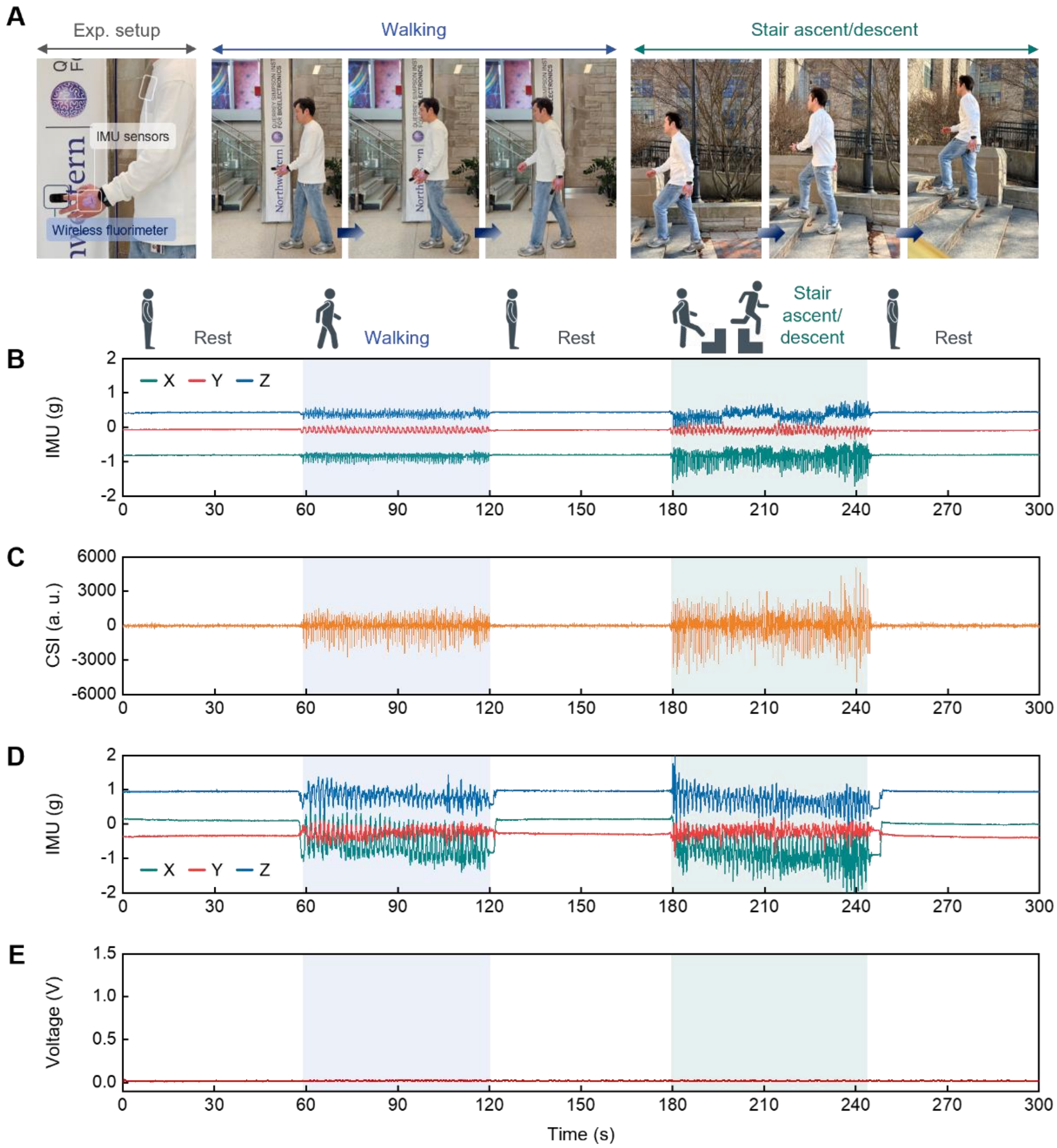

Fig. S51. Signal stability during free movement in a human subject. (A) Photographs of the experimental setup and subject movements during the protocol. Physiological signals, including **(B)** body movements (inertial measurement unit, IMU) and **(C)** cardiac sound intensity (CSI) recorded using a wearable sensor attached to the chest. Created in BioRender. Rogers, J. (2026) https://biorender.com/vk3u5ep. **(D)** Physical movements measured by a wearable sensor placed on the dorsal hand. **(E)** Signals obtained by wireless fluorimeter system positioned on the fingertip during the protocol.

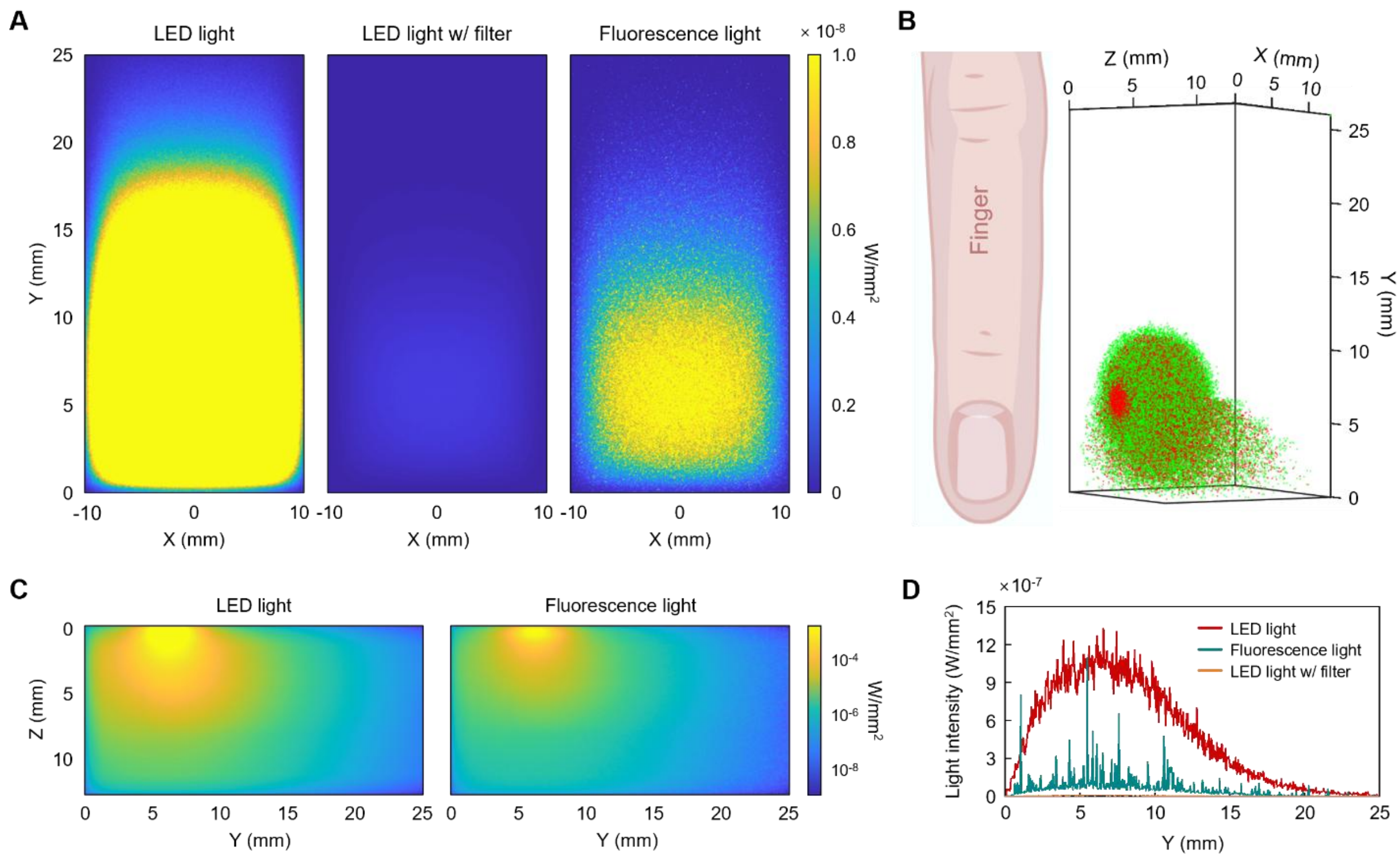

Fig. S52. Monte Carlo simulation assessing the feasibility of fluorescence sensing from a human finger using a finger-clip device. (A) Light intensity distributions at the bottom surface. **(B)** Three-dimensional light intensity distribution. Created in BioRender. Rogers, J. (2026) https://biorender.com/vk3u5ep. **(C)** Light intensity distribution in a cross-sectional plane. **(D)** Light intensity distribution at the bottom surface along $X = 0$.

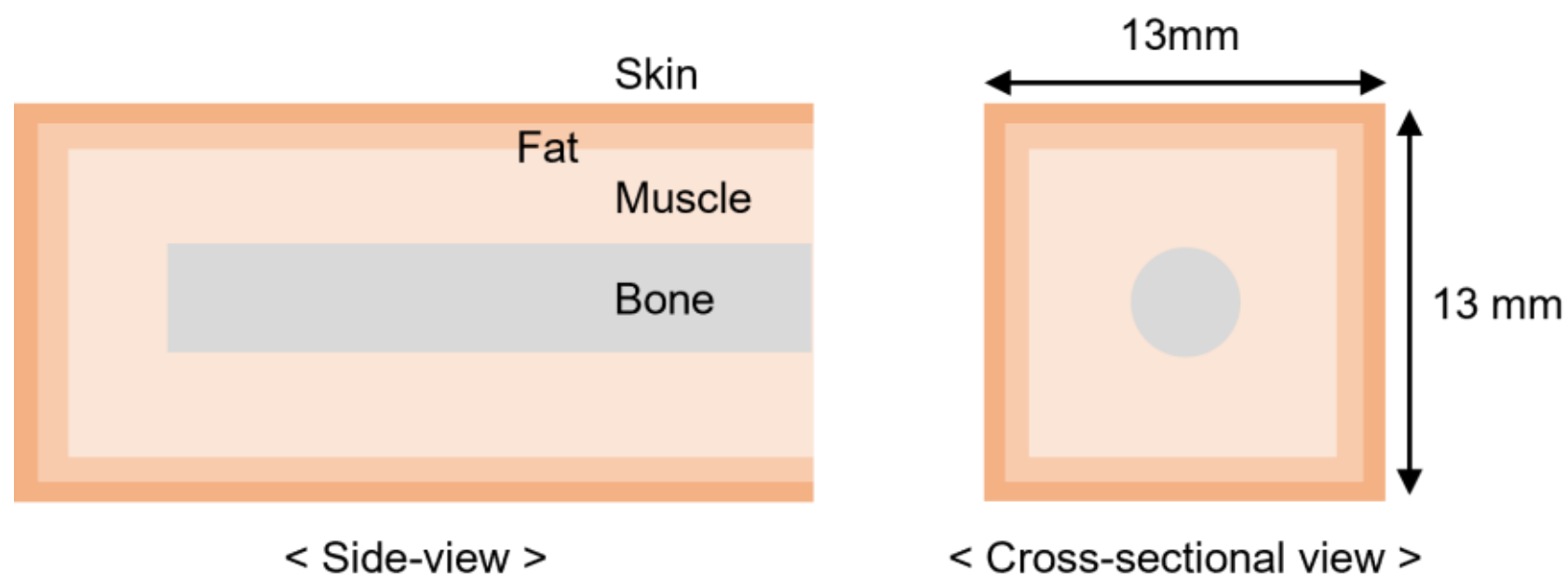

Fig. S53. The structure of the finger used in simulation.

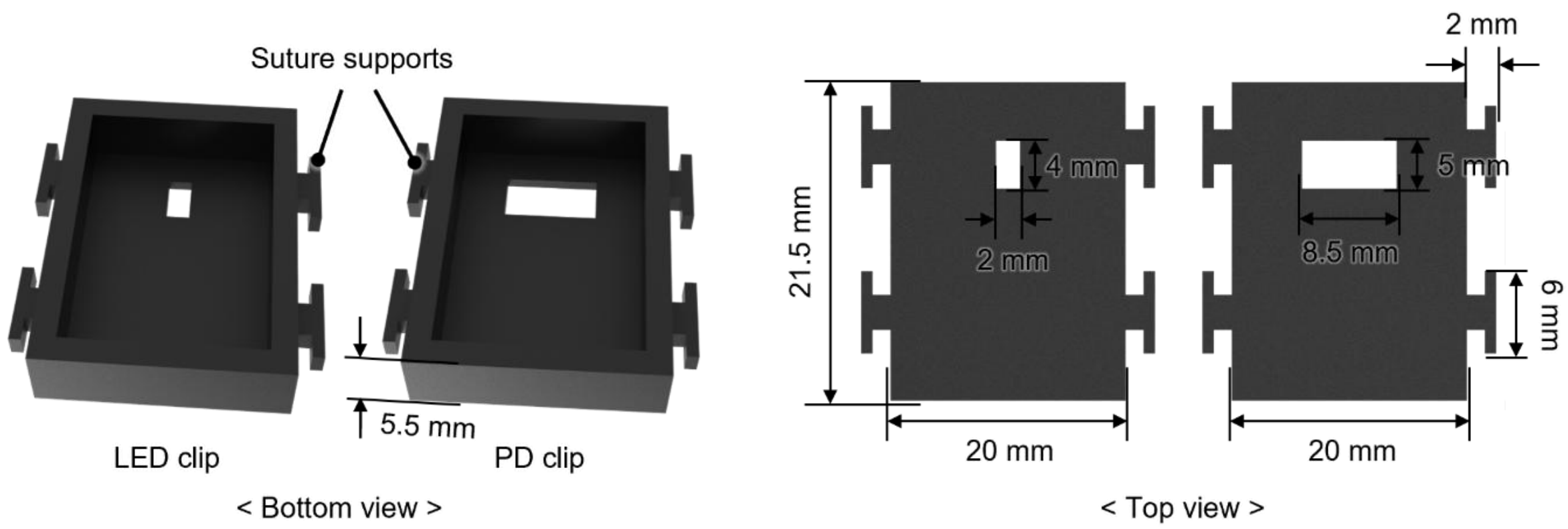

Fig. S54. Tailored device designs for *ex vivo* human heart demonstration. Dimensions of device designed for the placement on the surface of human heart.

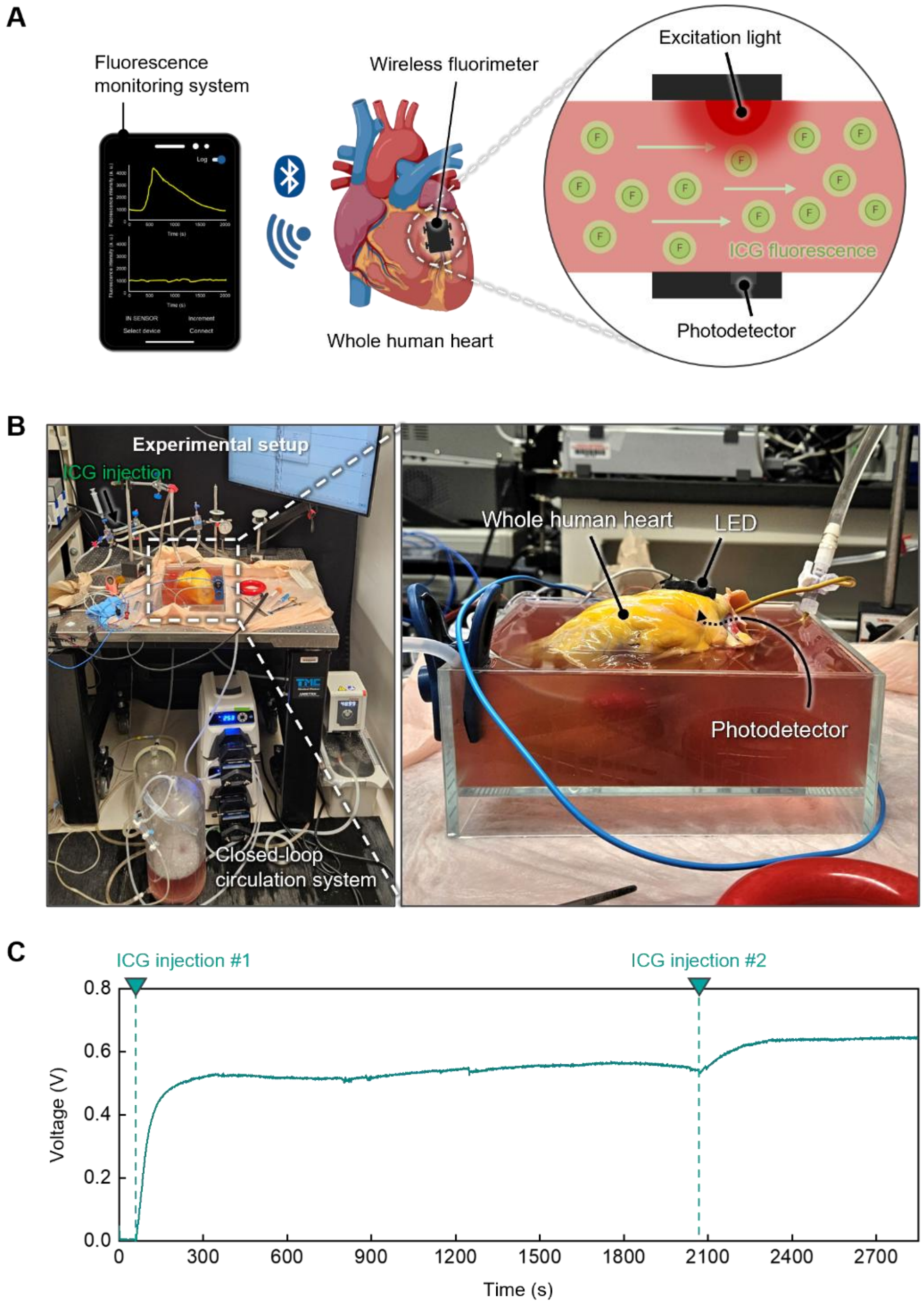

Fig. S55. ***Ex vivo*** **demonstration of fluorescence sensing in whole human heart. (A)** Schematic illustration and **(B)** photograph images of the experimental setup. Created in BioRender. Rogers, J. (2026) https://biorender.com/vk3u5ep. **(C)** Fluorescence response dynamics in the whole human heart.

System (company)	Fluorescence measurement method	Main purpose	Size (weight)	Sensing area	Primary functionality	Image
SPY Elite (Stryker) (*72, 73*)	NIR fluorescence imaging	Surgical visualization of microvascular blood flow and perfusion in tissue intraoperatively	Tower-based cart system (substantial weight)	~ 20 × 20 cm^2	• Ionizing radiation-free<br>• Repeatable technique	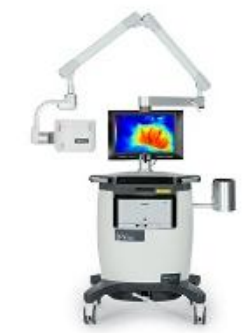
VEQTRON™ ICG (AMNOTEC) (*74*)	NIR fluorescence imaging	Intraoperative visualization of visceral perfusion, biliary structures, and lymph node mapping	Tower-based cart system (substantial weight)	~ 15 × 15 cm^2	• Minimally invasive procedure<br>• 4K imaging system	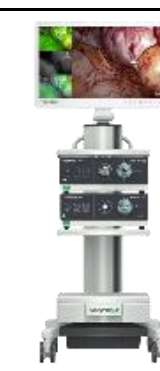
EleVision™ IR Platform (Medtronic) (*75, 76*)	NIR fluorescence imaging	Visualization and assess tissue perfusions	Tower-based cart system (substantial weight)	~ 15 × 15 cm^2	• Fluorescence signal intensity measurements in open and laparoscopic procedures	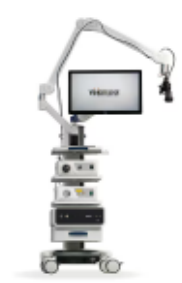
FLUOBEAM LM (GETINGE) (*77-79*)	NIR fluorescence imaging	Visualization of vessels and blood flows for organ transplants and plastic surgery Evaluation of the tissue perfusion	Bulky (non-portable)	~ 8 × 8 cm^2	• Ergonomical system<br>• Portable system	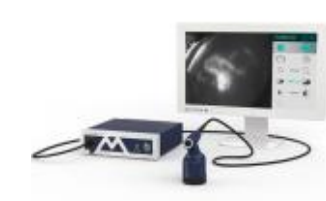
IC-Flow™ Handheld System (Diagnostic Green) (*80, 81*)	NIR fluorescence imaging	Visualization of blood flow and lymphatic flow	Portable (lightweight)	~ 5 × 5 cm^2	• Portable and handheld system	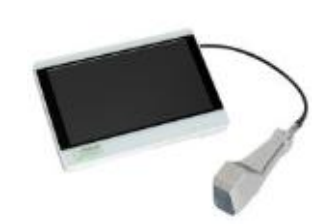
**(This work)**	**Electronics-based measurement**	**Quantitative analysis and continuous monitoring of fluorescence signals**	**20.0 mm × 32.5 mm × 23.0 mm (9.7 g)**	**~ 0.5 × 0.8 cm^2**	• **Fully wireless, ultra-compact, non-invasive system**	

Table S1. Conventional medical systems for ICG fluorescence sensing and comparison to the system reported here.

Sublayer	Thickness (mm)	V_b	V_w	V_m
Stratum corneum	0.02	0	0.05	0
Epidermis	0.25	0	0.2	0.1
Papillary dermis	0.1	0.04	0.5	0
Upper blood net dermis	0.08	0.3	0.6	0
Reticular dermis	0.2	0.04	0.7	0
Deep blood net dermis	0.3	0.1	0.7	0

Table S2. Layers of the skin on the fingers.

Participant No.	Thickness (mm)	Sex	Ethnicity / age	Health conditions
#01	13	Male	Asian / 32	None
#02	13	Female	White / 22	None
#03	13	Female	Asian / 29	None
#04	13	Male	Asian / 39	None
#05	13	Male	Asian / 30	None

Table S3. Participant information for thickness-dependent device calibration on the index finger.

Participant No.	Measurement site	Thickness (mm)	Sex/Ethnicity/Age	Health conditions
#01	Thumb	15	Male/Asian/32	None
	Index finger	13		
	Middle finger	13		
	Ring finger	13		
	Little finger	10		

Table S4. Thickness information for different fingers of a single participant.

REFERENCES

1. V. Ntziachristos, J. Ripoll, L. V. Wang, R. Weissleder, Looking and listening to light: The evolution of whole-body photonic imaging. *Nat. Biotechnol.* **23**, 313–320 (2005).

2. G. Hong, S. Diao, A. L. Antaris, H. Dai, Carbon nanomaterials for biological imaging and nanomedicinal therapy. *Chem. Rev.* **115**, 10816–10906 (2015).

3. G. Hong, A. L. Antaris, H. Dai, Near-infrared fluorophores for biomedical imaging. *Nat. Biomed. Eng.* **1**, 0010 (2017).

4. K. M. Tichauer, K. S. Samkoe, J. R. Gunn, S. C. Kanick, P. J. Hoopes, R. J. Barth, P. A. Kaufman, T. Hasan, B. W. Pogue, Microscopic lymph node tumor burden quantified by macroscopic dual-tracer molecular imaging. *Nat. Med.* **20**, 1348–1353 (2014).

5. Z. Ou, Y.-S. Duh, N. J. Rommelfanger, C. H. Keck, S. Jiang, K. Brinson Jr, S. Zhao, E. L. Schmidt, X. Wu, F. Yang, Achieving optical transparency in live animals with absorbing molecules. *Science* **385**, eadm6869 (2024).

6. H. Fang, Y. Chen, Z. Jiang, W. He, Z. Guo, Fluorescent probes for biological species and microenvironments: From rational design to bioimaging applications. *Accounts Chem. Res.* **56**, 258–269 (2023).

7. D. J. Waterhouse, C. R. Fitzpatrick, B. W. Pogue, J. P. O'Connor, S. E. Bohndiek, A roadmap for the clinical implementation of optical-imaging biomarkers. *Nat. Biomed. Eng.* **3**, 339–353 (2019).

8. Q. Wu, Z. Zhou, L. Xu, H. Zhong, B. Xiong, T. Ren, Z. Li, L. Yuan, X.-B. Zhang, Multivalent supramolecular fluorescent probes for accurate disease imaging. *Sci. Adv.* **10**, eadp8719 (2024).

9. X. Wang, Q. Ding, R. R. Groleau, L. Wu, Y. Mao, F. Che, O. Kotova, E. M. Scanlan, S. E. Lewis, P. Li, Fluorescent probes for disease diagnosis. *Chem. Rev.* **124**, 7106–7164 (2024).

10. M. C. Skala, K. M. Riching, A. Gendron-Fitzpatrick, J. Eickhoff, K. W. Eliceiri, J. G. White, N. Ramanujam, In vivo multiphoton microscopy of NADH and FAD redox states, fluorescence

lifetimes, and cellular morphology in precancerous epithelia. *Proc. Natl. Acad. Sci. U.S.A.* **104**, 19494–19499 (2007).

11. M. Saif, W. J. Kwanten, J. A. Carr, I. X. Chen, J. M. Posada, A. Srivastava, J. Zhang, Y. Zheng, M. Pinter, S. Chatterjee, Non-invasive monitoring of chronic liver disease via near-infrared and shortwave-infrared imaging of endogenous lipofuscin. *Nat. Biomed. Eng.* **4**, 801–813 (2020).

12. C. Stringari, A. Cinquin, O. Cinquin, M. A. Digman, P. J. Donovan, E. Gratton, Phasor approach to fluorescence lifetime microscopy distinguishes different metabolic states of germ cells in a live tissue. *Proc. Natl. Acad. Sci. U.S.A.* **108**, 13582–13587 (2011).

13. Y. Pan, J.-P. Volkmer, K. E. Mach, R. V. Rouse, J.-J. Liu, D. Sahoo, T. C. Chang, T. J. Metzner, L. Kang, M. Van De Rijn, Endoscopic molecular imaging of human bladder cancer using a CD47 antibody. *Sci. Transl. Med.* **6**, 260ra148 (2014).

14. C. S. Morrow, P. Yao, C. A. Vergani-Junior, P. V. Anekal, P. Montero Llopis, J. W. Miller, B. A. Benayoun, W. B. Mair, Endogenous mitochondrial NAD(P)H fluorescence can predict lifespan. *Commun. Biol.* **7**, 1551 (2024).

15. R. Pal, T. M. Lwin, M. Krishnamoorthy, H. R. Collins, C. D. Chan, A. Prilutskiy, M. P. Nasrallah, T. H. Dijkhuis, S. Shukla, A. L. Kendall, Fluorescence lifetime of injected indocyanine green as a universal marker of solid tumours in patients. *Nat. Biomed. Eng.* **7**, 1649–1666 (2023).

16. B. E. Schaafsma, J. S. D. Mieog, M. Hutteman, J. R. Van der Vorst, P. J. Kuppen, C. W. Löwik, J. V. Frangioni, C. J. Van de Velde, A. L. Vahrmeijer, The clinical use of indocyanine green as a near-infrared fluorescent contrast agent for image-guided oncologic surgery. *J. Surg. Oncol.* **104**, 323–332 (2011).

17. A. Matsui, E. Tanaka, H. S. Choi, J. H. Winer, V. Kianzad, S. Gioux, R. G. Laurence, J. V. Frangioni, Real-time intra-operative near-infrared fluorescence identification of the extrahepatic bile ducts using clinically available contrast agents. *Surgery* **148**, 87–95 (2010).

18. S. L. Troyan, V. Kianzad, S. L. Gibbs-Strauss, S. Gioux, A. Matsui, R. Oketokoun, L. Ngo, A. Khamene, F. Azar, J. V. Frangioni, The FLARE™ intraoperative near-infrared fluorescence imaging system: A first-in-human clinical trial in breast cancer sentinel lymph node mapping. *Ann. Surg. Oncol.* **16**, 2943–2952 (2009).

19. J. A. Carr, D. Franke, J. R. Caram, C. F. Perkinson, M. Saif, V. Askoxylakis, M. Datta, D. Fukumura, R. K. Jain, M. G. Bawendi, Shortwave infrared fluorescence imaging with the clinically approved near-infrared dye indocyanine green. *Proc. Natl. Acad. Sci. U.S.A.* **115**, 4465–4470 (2018).

20. A. L. Vahrmeijer, M. Hutteman, J. R. Van Der Vorst, C. J. Van De Velde, J. V. Frangioni, Image-guided cancer surgery using near-infrared fluorescence. *Nat. Rev. Clin. Oncol.* **10**, 507–518 (2013).

21. D. C. Ng, T. Tokuda, A. Yamamoto, M. Matsuo, M. Nunoshita, H. Tamura, Y. Ishikawa, S. Shiosaka, J. Ohta, On-chip biofluorescence imaging inside a brain tissue phantom using a CMOS image sensor for in vivo brain imaging verification. *Sens. Actuator B. Chem.* **119**, 262–274 (2006).

22. F. Wang, Y. Zhong, O. Bruns, Y. Liang, H. Dai, In vivo NIR-II fluorescence imaging for biology and medicine. *Nat. Photonics* **18**, 535–547 (2024).

23. R. Tian, H. Ma, S. Zhu, J. Lau, R. Ma, Y. Liu, L. Lin, S. Chandra, S. Wang, X. Zhu, Multiplexed NIR-II probes for lymph node-invaded cancer detection and imaging-guided surgery. *Adv. Mater.* **32**, e1907365 (2020).

24. R. R. Zhang, A. B. Schroeder, J. J. Grudzinski, E. L. Rosenthal, J. M. Warram, A. N. Pinchuk, K. W. Eliceiri, J. S. Kuo, J. P. Weichert, Beyond the margins: Real-time detection of cancer using targeted fluorophores. *Nat. Rev. Clin. Oncol.* **14**, 347–364 (2017).

25. Z. Hu, C. Fang, B. Li, Z. Zhang, C. Cao, M. Cai, S. Su, X. Sun, X. Shi, C. Li, First-in-human liver-tumour surgery guided by multispectral fluorescence imaging in the visible and near-infrared-I/II windows. *Nat. Biomed. Eng.* **4**, 259–271 (2020).

26. K. Wang, Y. Du, Z. Zhang, K. He, Z. Cheng, L. Yin, D. Dong, C. Li, W. Li, Z. Hu, Fluorescence image-guided tumour surgery. *Nat. Rev. Bioeng.* **1**, 161–179 (2023).

27. P. De Nardi, U. Elmore, G. Maggi, R. Maggiore, L. Boni, E. Cassinotti, U. Fumagalli, M. Gardani, S. De Pascale, P. Parise, Intraoperative angiography with indocyanine green to assess anastomosis perfusion in patients undergoing laparoscopic colorectal resection: Results of a multicenter randomized controlled trial. *Surg. Endosc.* **34**, 53–60 (2020).

28. X. Cai, H. Zhang, P. Wei, Q. Liu, D. Sheng, Z. Li, B. Zhang, G. Tang, W. Zhao, Z. Ye, A wireless optoelectronic probe to monitor oxygenation in deep brain tissue. *Nat. Photonics* **18**, 492–500 (2024).

29. W. Ling, X. Shang, C. Yu, C. Li, K. Xu, L. Feng, Y. Wei, T. Tang, X. Huang, Miniaturized implantable fluorescence probes integrated with metal–organic frameworks for deep brain dopamine sensing. *ACS Nano* **18**, 10596–10608 (2024).

30. L. Lu, P. Gutruf, L. Xia, D. L. Bhatti, X. Wang, A. Vazquez-Guardado, X. Ning, X. Shen, T. Sang, R. Ma, Wireless optoelectronic photometers for monitoring neuronal dynamics in the deep brain. *Proc. Natl. Acad. Sci. U.S.A.* **115**, E1374–E1383 (2018).

31. M. Corsi, E. Maurina, S. Surdo, E. Vandini, E. Daini, A. Vilella, G. Leo, M. Farshchian, G. Grisendi, G. Golinelli, In vivo and in situ monitoring of doxorubicin pharmacokinetics with an implantable bioresorbable optical sensor. *Sci. Adv.* **11**, eads0265 (2025).

32. S. Y. Lee, J. M. Pakela, K. Na, J. Shi, B. J. McKenna, D. M. Simeone, E. Yoon, J. M. Scheiman, M.-A. Mycek, Needle-compatible miniaturized optoelectronic sensor for pancreatic cancer detection. *Sci. Adv.* **6**, eabc1746 (2020).

33. N. T. Quirion, M. Madrid, J. Chang, A. Fehr, E. Rytkin, N. Shields, B. Burke, A. Elekeokwuri, I. R. Efimov, L. Lu, A soft multimodal optoelectronic array interface for multiparametric mapping of heart function in vivo. *Sci. Adv.* **11**, eads8608 (2025).

34. International Electrotechnical Commission, “Medical electrical equipment—Part 1: General requirements for basic safety and essential performance” in *IEC 60601-1:2005*, (International Electrotechnical Commission, 2005); https://webstore.iec.ch/en/publication/2606.

35. A. Bozkurt, A. Rosen, H. Rosen, B. Onaral, A portable near infrared spectroscopy system for bedside monitoring of newborn brain. *Biomed. Eng. Online* **4**, 29 (2005).

36. A. Wunsch, K. Matuschka, A controlled trial to determine the efficacy of red and near-infrared light treatment in patient satisfaction, reduction of fine lines, wrinkles, skin roughness, and intradermal collagen density increase. *Photomed. Laser Surg.* **32**, 93–100 (2014).

37. C. Jonak, H. Skvara, R. Kunstfeld, F. Trautinger, J. A. Schmid, Intradermal indocyanine green for in vivo fluorescence laser scanning microscopy of human skin: A pilot study. *PLOS ONE* **6**, e23972 (2011).

38. G. E. Glass, Photobiomodulation: A systematic review of the oncologic safety of low-level light therapy for aesthetic skin rejuvenation. *Aesthet. Surg. J.* **43**, NP357–NP371 (2023).

39. A. Bozkurt, B. Onaral, Safety assessment of near infrared light emitting diodes for diffuse optical measurements. *Biomed. Eng. Online* **3**, 9 (2004).

40. D. Hu, M. Zha, H. Zheng, D. Gao, Z. Sheng, Recent advances in indocyanine green-based probes for second near-infrared fluorescence imaging and therapy. *Research* **8**, 0583 (2025).

41. S. A. Prahl, Tabulated molar extinction coefficient for hemoglobin in water (1999); http://omlc.ogi.edu/spectra/hemoglobin/summary.html.

42. E. D. Cosco, I. Lim, E. M. Sletten, Photophysical properties of indocyanine green in the shortwave infrared region. *ChemPhotoChem* **5**, 727–734 (2021).

43. X. Li, C. Patrick Yue, 3 GHz, 1 mW inverter-based TIA with capacitive feedback for enhanced gain and sensitivity in VLC applications. *Electron. Lett.* **55**, 469–471 (2019).

44. T. Desmettre, J. Devoisselle, S. Mordon, Fluorescence properties and metabolic features of indocyanine green (ICG) as related to angiography. *Surv. Ophthalmol.* **45**, 15–27 (2000).

45. R. F. Spaide, J. M. Klancnik, M. J. Cooney, Retinal vascular layers imaged by fluorescein angiography and optical coherence tomography angiography. *JAMA Ophthalmol.* **133**, 45–50 (2015).

46. L. van Manen, H. J. Handgraaf, M. Diana, J. Dijkstra, T. Ishizawa, A. L. Vahrmeijer, J. S. D. Mieog, A practical guide for the use of indocyanine green and methylene blue in fluorescence-guided abdominal surgery. *J. Surg. Oncol.* **118**, 283–300 (2018).

47. F. W. Blaisdell, The pathophysiology of skeletal muscle ischemia and the reperfusion syndrome: A review. *Cardiovasc. Surg.* **10**, 620–630 (2002).

48. J. K. Lunney, A. Van Goor, K. E. Walker, T. Hailstock, J. Franklin, C. Dai, Importance of the pig as a human biomedical model. *Sci. Transl. Med.* **13**, eabd5758 (2021).

49. A. De Gasperi, E. Mazza, M. Prosperi, Indocyanine green kinetics to assess liver function: Ready for a clinical dynamic assessment in major liver surgery? *World J. Hepatol.* **8**, 355–367 (2016).

50. C. P. Sabino, A. M. Deana, T. M. Yoshimura, D. F. Da Silva, C. M. França, M. R. Hamblin, M. S. Ribeiro, The optical properties of mouse skin in the visible and near infrared spectral regions. *J. Photochem. Photobiol. B. Biol.* **160**, 72–78 (2016).

51. E. Salomatina, A. Yaroslavsky, Evaluation of the in vivo and ex vivo optical properties in a mouse ear model. *Phys. Med. Biol.* **53**, 2797–2807 (2008).

52. C. A. Haque, S. Hossain, T.-H. Kwon, K.-D. Kim, Noninvasive in vivo estimation of blood-glucose concentration by Monte Carlo Simulation. *Sensors* **21**, 4918 (2021).

53. S. Chatterjee, P. A. Kyriacou, Monte Carlo analysis of optical interactions in reflectance and transmittance finger photoplethysmography. *Sensors* **19**, 789 (2019).

54. D. J. Segelstein, “The complex refractive index of water,” thesis, University of Missouri-Kansas City (1981).

55. S. L. Jacques, “Skin optics summary,” *Oregon Medical Laser Center News* (1998).

56. S. L. Jacques, Optical properties of biological tissues: A review. *Phys. Med. Biol.* **58**, R37–R61 (2013).

57. R. L. Van Veen, H. J. Sterenborg, A. Pifferi, A. Torricelli, E. Chikoidze, R. Cubeddu, Determination of visible near-IR absorption coefficients of mammalian fat using time-and spatially resolved diffuse reflectance and transmission spectroscopy. *J. Biomed. Opt.* **10**, 054004 (2005).

58. A. N. Bashkatov, E. A. Genina, V. I. Kochubey, V. V. Tuchin, in *Saratov Fall Meeting 2005: Optical Technologies in Biophysics and Medicine VII* (SPIE, 2006), vol. 6163, pp. 306–316.

59. C. R. Simpson, M. Kohl, M. Essenpreis, M. Cope, Near-infrared optical properties of ex vivo human skin and subcutaneous tissues measured using the Monte Carlo inversion technique. *Phys. Med. Biol.* **43**, 2465–2478 (1998).

60. M. Mariello, J. D. Rosenthal, F. Cecchetti, M. Gao, A. K. Skrivervik, Y. Leterrier, S. P. Lacour, Wireless, battery-free, and real-time monitoring of water permeation across thin-film encapsulation. *Nat. Commun.* **15**, 7443 (2024).

61. H. Zou, Z. Zhou, M. Huang, W. Li, B. Yang, X. Zhao, T. Li, L. Xu, T. Wang, L. Wang, NFC/RFID-enabled wearables and implants for biomedical applications. *Microsyst. Nanoeng.* **11**, 191 (2025).

62. S. Mordon, J. M. Devoisselle, S. Soulie-Begu, T. Desmettre, Indocyanine green: Physicochemical factors affecting its fluorescence in vivo. *Microvasc. Res.* **55**, 146–152 (1998).

63. R. Benson, H. Kues, Fluorescence properties of indocyanine green as related to angiography. *Phys. Med. Biol.* **23**, 159–163 (1978).

64. M. D. Mokrousov, M. V. Novoselova, J. Nolan, W. Harrington, P. Rudakovskaya, D. N. Bratashov, E. I. Galanzha, J. P. Fuenzalida-Werner, B. P. Yakimov, G. Nazarikov, Amplification of photoacoustic effect in bimodal polymer particles by self-quenching of indocyanine green. *Biomed. Opt. Express* **10**, 4775–4789 (2019).

65. N. Argent, J. Liles, D. Rodham, C. Clayton, R. Wilkinson, P. Baylis, A new method for measuring the blood volume of the rat using ^{113m}Indium as a tracer. *Lab. Anim* **28**, 172–175 (1994).

66. R. J. Probst, J. M. Lim, D. N. Bird, G. L. Pole, A. K. Sato, J. R. Claybaugh, Gender differences in the blood volume of conscious Sprague-Dawley rats. *J. Amer. Assoc. Lab. Anim. Sci.* **45**, 49–52 (2006).

67. H. Lee, M. Blaufox, Blood volume in the rat. *J. Nucl. Med.* **26**, 72–76 (1985).

68. J. P. Hannon, C. A. Bossone, W. G. Rodkey, Splenic red cell sequestration and blood volume measurements in conscious pigs. *Am. J. Physiol. Regul. Integr. Comp. Physiol.* **248**, R293–R301 (1985).

69. A. Chalkias, E. Laou, N. Papagiannakis, V. Spyropoulos, E. Kouskouni, K. Theodoraki, T. Xanthos, Assessment of dynamic changes in stressed volume and venous return during hyperdynamic septic shock. *J. Pers. Med.* **12**, 724 (2022).

70. A. Lervik, S. Forr Toverud, J. Bohlin, H. A. Haga, Macrocirculatory parameters and oxygen debt indices in pigs during propofol or alfaxalone anesthesia when subjected to experimental stepwise hemorrhage. *Front. Vet. Sci.* **8**, 664112 (2021).

71. C. Sapsanis, M. Sophocleous, A. G. Andreou, J. Georgiou, Trade-offs in sensor systems design: A tutorial. *IEEE Sensors J.* **22**, 10040–10061 (2022).

72. M. Sood, P. Glat, Potential of the SPY intraoperative perfusion assessment system to reduce ischemic complications in immediate postmastectomy breast reconstruction. *Ann. Surg. Innov. Res.* **7**, 9 (2013).

73. B. Popiel, D. Gupta, S. Misra, Value of an intraoperative real time tissue perfusion assessment system following a nipple-sparing radical mastectomy for advanced breast cancer. *Int. J. Surg. Case Rep.* **5**, 30–33 (2014).

74. A. Preziosi, C. Cirelli, D. Waterhouse, L. Privitera, P. De Coppi, S. Giuliani, State of the art medical devices for fluorescence-guided surgery (FGS): Technical review and future developments. *Surg. Endosc.* **38**, 6227–6236 (2024).

75. A. K. Bigdeli, E. Gazyakan, V. J. Schmidt, F. J. Hernekamp, L. Harhaus, T. Henzler, T. Kremer, U. Kneser, C. Hirche, Indocyanine green fluorescence for free-flap perfusion imaging revisited: Advanced decision making by virtual perfusion reality in visionsense fusion imaging angiography. *Surg. Innov.* **23**, 249–260 (2016).

76. A. V. DSouza, H. Lin, E. R. Henderson, K. S. Samkoe, B. W. Pogue, Review of fluorescence guided surgery systems: Identification of key performance capabilities beyond indocyanine green imaging. *J. Biomed. Opt.* **21**, 080901 (2016).

77. J. Kim, M. W. Han, K. Y. Hong, Prospective clinical trial for predicting mastectomy skin flap necrosis with indocyanine green angiography in implant-based prepectoral breast reconstruction. *Aesthet. Plast. Surg.* **48**, 4937–4944 (2024).

78. C. Ngô, S. Sharifzadehgan, C. Lecurieux-Lafayette, H. Belhouari, D. Rousseau, H. Bonsang-Kitzis, L. Crouillebois, V. Balaya, S. Oudard, F. Lécuru, Indocyanine green for sentinel lymph node detection in early breast cancer: Prospective evaluation of detection rate and toxicity—The FLUOBREAST trial. *Breast J.* **26**, 2357–2363 (2020).

79. M. Salgarello, V. Pino, D. M. Pagliara, G. Visconti, Preventing soft tissue complications in secondary aesthetic breast surgery using indocyanin green angiography. *Aesthet. Surg. J.* **43**, 665–672 (2023).

80. V. Brebant, N. Heine, P. Lamby, P. Heidekrueger, A. Forte, L. Prantl, T. Aung, Augmented reality of indocyanine green fluorescence in simplified lymphovenous anastomosis in lymphatic surgery. *Clin. Hemorheol. Microcirc.* **73**, 125–133 (2019).

81. B. Ponikowska, J. Biegus, M. Fudim, G. Iwanek, M. Guzik, R. Przybylski, A. Szuba, A. Chachaj, R. Zymliński, Lower extremity lymphatic flow/drainage assessment by indocyanine green fluorescent lymphography in heart failure patients. *JACC Basic Transl. Sci.* **9**, 906–917 (2024).
